# Beyond Common Energy Transfer: Intramolecular Electron Transfer Cascade Controls Triplet Population of a Long-Lived Iron-Anthracene Molecular Dyad

**DOI:** 10.1021/acscentsci.5c01040

**Published:** 2025-08-04

**Authors:** Felix Glaser, Giovanni M. Beneventi, Alejandro Cadranel, Ludovic Troian-Gautier

**Affiliations:** ‡ 83415 UCLouvain, Institut de la Matière Condensée et des Nanosciences (IMCN), Molecular Chemistry, Materials and Catalysis (MOST), Place Louis Pasteur 1/L4.01.02, B-1348 Louvain-la-Neuve, Belgium; δ 9171 Friedrich-Alexander-Universität Erlangen-Nürnberg (FAU), Physical Chemistry I, Egerlandstr. 3, 91058 Erlangen, Germany; ± Friedrich-Alexander-Universität Erlangen-Nürnberg (FAU), Interdisciplinary Center for Molecular Materials, Egerlandstr. 3, 91058 Erlangen, Germany; Σ Universidad de Buenos Aires, Facultad de Ciencias Exactas y Naturales, Departamento de Química Inorgánica, Analítica y Química Física, Pabellón 2, Ciudad Universitaria, C1428EHA Buenos Aires, Argentina; ⊥ CONICET−Universidad de Buenos Aires, Instituto de Química-Física de Materiales, Medio Ambiente y Energía (INQUIMAE), Pabellón 2, Ciudad Universitaria, C1428EHA Buenos Aires, Argentina; ϕ Wel Research Institute, Avenue Pasteur 6, 1300 Wavre, Belgium

## Abstract

An iron-anthracene dyad was recently used to populate a microsecond-lived nonluminescent (dark) triplet state, but with a surprisingly low triplet population yield. In-depth spectroscopic experiments highlight that direct energy transfer does not take place, but rather an intramolecular electron-transfer occurs, generating the corresponding reduced iron center and oxidized anthracene moiety, and the final triplet dark state is populated following charge recombination. This electron-transfer cascade reaction mechanism provided the unique opportunity to control the energy level of the charge-separated state relative to the energy of the triplet state by changing the solvent polarity. As such, the triplet formation yield increased from 5% in acetonitrile to 75% in dichloromethane. This outlines an unreported mechanistic pathway for first-row transition metal complexes to populate long-lived excited states and provides design guidelines that differ between d^5^ and prototypical d^6^ photosensitizers. The d^5^ electronic configuration enables population of the final triplet energy acceptor via a cascade of electron transfer that does not formally require intersystem crossing or spin-flip transitions, thus also minimizing energy loss channels. Although the energy of the final triplet state is important, our findings highlight that the redox potentials of the excited photosensitizer and final energy acceptor moiety are pivotal to efficiently populate dark triplet states.

## Graphical abstract

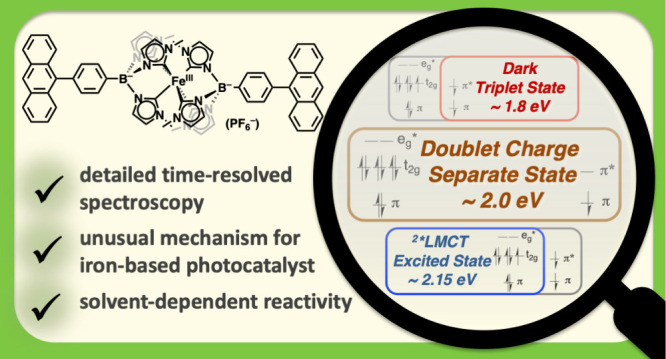

## Introduction

Ru^II^, Ir^III^ and Os^II^ represent the archetypical metal centers for the development of active photosensitizers for a plethora of light-induced transformations. These photosensitizers present attractive properties, such as long excited-state lifetimes, tunable ground- and excited-state redox properties, and appreciable visible light absorption. However, the scarcity of these metal poses a challenge and thus, alternatives must be found.^1–5^ Within the possible earth-abundant substitutes, iron is the most obvious candidate, but it turned out to be one of the most challenging ones to use as Fe^II^ photosensitizers usually suffer from extremely short excited-state lifetimes and redox potentials that are less versatile than their Ru^II^, Ir^III^ and Os^II^ counterparts.^6^ Several strategies have been developed over the years to increase the excited-state lifetime toward the nanosecond time scale.^7–14^

Recently, combining iron complexes with triplet energy acceptors received some attention,^15–17^ and specifically we reported a molecular dyad ([Fe­(L^PhAn^)_2_]^+^) consisting of a Fe^III^ photosensitizer connected to an anthracene moiety that exhibited a dark excited-state lifetime of ∼11.5 μs under argon and 165 ns under air in acetonitrile.^15^ The key concept to reach these extraordinary lifetimes relies on the reasonably fast depopulation of the Fe-centered excited state to populate the nonluminescent triplet excited state of the attached polyaromatic hydrocarbon, presumably via an irreversible doublet-triplet energy transfer (DTET). Photophysical analysis of the dyad’s excited state exhibited signatures of the two expected states: the doublet ligand-to-metal charge transfer state (^2^LMCT) of the photosensitizer, detectable by the fluorescence of this initial state, and the triplet excited state of the 9-phenylanthracene moiety (^3*^PhAn), detectable by nanosecond transient absorption (ns-TA) spectroscopy.^15^ Compared to the unsubstituted reference photosensitizer, this dyad not only led to an increased excited-state lifetime, but also allowed to target ^1^O_2_ photosensitization and drastically increased the cage escape yield for oxidative quenching by almost one order of magnitude (42% vs 4.5%).^15^

Thus, this concept could potentially drastically extend the possibilities of iron photosensitizers for photocatalytic applications.^18–20^ However, the major challenge with this novel approach was the incomplete population of ^3*^PhAn within the dyad, which was initially estimated to be only ∼10%. In fact, several loss channels next to the thermodynamically feasible doublet-triplet energy transfer (ΔG ≈ −0.35 eV in acetonitrile) are possible, such as (i) direct deactivation from the iron-based ^2^LMCT to the ground state as well as (ii) an alternative intramolecular electron transfer (ΔG ≈ −0.1 eV) quenching pathway to yield the reduced iron center and the oxidized PhAn moiety, [(L^PhAn^)­Fe^II^(L^PhAn•+^)]^+^. Hence, spectroscopic measurements with higher temporal resolution are needed to distinguish the different pathways and further improve the ^3*^PhAn population.

Here we report the femtosecond transient-absorption (fs-TA) spectroscopy study of [Fe­(L^PhAn^)_2_]^+^ in several solvents to disentangle the mechanistic pathway leading to the population of the final long-lived triplet excited state, ^3*^PhAn (Figure 1). In all investigated solvents, the population of the long-lived triplet excited state is downhill from the initial ^2^LMCT. Contrary to expectations, the fs-TA results highlight that ultrafast intramolecular electron transfer through reductive quenching of the ^2^LMCT by PhAn affording [(L^PhAn^)­Fe^II^(L^PhAn•+^)]^+^ is operative. The triplet state is formed upon charge recombination only, with no evidence for direct doublet-triplet energy transfer. This key pathway stands in contrast with the recently reported bimolecular quenching between PhAn and the unsubstituted Fe^III^ photosensitizer, where clear evidence of DTET was observed in DMSO.^17^ Here, a closer analysis of this diffusional bimolecular quenching pathway confirmed these results but also revealed minor contributions from an electron transfer cascade as observed as the predominant pathway within the dyad.

**Figure 1:**
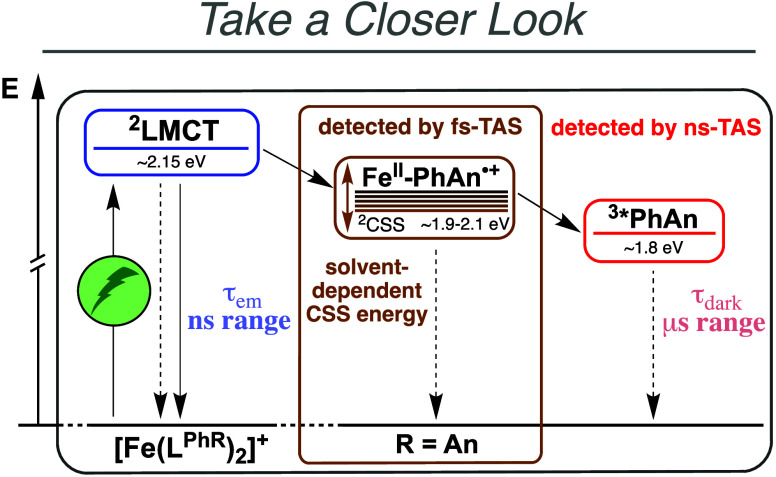
Energy scheme for the excited-state deactivation of [Fe­(L^PhAn^)_2_]^+^. PhAn = 9-phenylanthracene. TAS = transient absorption spectroscopy. LMCT = ligand-to-metal charge transfer. CSS = charge-separated state. Solid and dotted vertical arrows indicate radiative and nonradiative decay processes, respectively.

Additionally, the ultrafast spectroscopic investigation was carried out in acetonitrile and dichloromethane, i.e., two solvents largely separated by their dielectric constant, and indicated that similar pathways are operative but with significantly different rate constants for each individual step. Tetrahydrofuran, butyronitrile and acetone were also used to investigate the trend of ^3*^PhAn population. Significant differences in rate constants and ^3*^PhAn population yields were found and a good qualitative agreement between the calculated energy of the intermediate charge-separated state and the quenching efficiency of the iron-based ^2^LMCT excited state was found, thereby rationalizing the observed solvent-dependent behavior. While the mechanism, at first glance, resembles a spin–orbit charge-transfer intersystem crossing (SOCT ISC) known for (neutral) donor–acceptor dyads, the overall spin multiplicity remains a doublet throughout the excited-state decay cascade, from the (local) ^2^LMCT via the [(L^PhAn^)­Fe^II^(L^PhAn•+^)]^+^ charge-separated state to the final (local) ^3*^PhAn. This uncommon mechanism represents a novel pathway to populate spin states with different local multiplicity and is relevant for other dyads consisting of metal complexes with an electronic configuration different from the prototypical d^6^.

## Results and Discussion

The synthesis of [Fe­(L^Ph^)_2_]^+^ and [Fe­(L^PhAn^)_2_]^+^ was previously reported.^15^ We first investigated the ground state absorption and steady-state and time-resolved emission in five solvents (Figure 2), i.e., dichloromethane (DCM), tetrahydrofuran (THF), butyronitrile (BuCN), acetone, and acetonitrile (MeCN). For both [Fe­(L^Ph^)_2_]^+^ and [Fe­(L^PhAn^)_2_]^+^, the ground state absorption spectra were unaffected by the change in solvent (Figure 2a,b) and the dyad is essentially a linear combination of the absorption spectra of the unmodified complex and two PhAn moieties. The steady-state emission was identical for all solvents, with the exception of a slight blue-shift recorded in DCM, as reported in the literature for [Fe­(L^Ph^)_2_]^+^ (Figure 2a).^21^ While the emission lifetime from excited ^2^*­[Fe­(L^Ph^)_2_]^+^ was around 2 ns, with the exception of DCM (2.45 ns, Figure 2c), the emission lifetimes of the dyad presented a high solvent-dependence. In this case, the fluorescence lifetimes ranged between 1.35 ns in MeCN and 0.35 ns in DCM (Figure 2c). In addition, the emission quantum yields of [Fe­(L^PhAn^)_2_]^+^ decreased from 1.75% to 1.17% in MeCN and from 2.22% to 0.50% in DCM, respectively (Tables S1 and S2 in the Supporting Information). The origin of these changes was assessed by fs-TA spectroscopy. Note that in DCM, a biexponential decay was observed with a small relative intensity exhibiting an excited-state lifetime of ∼2 ns. This is attributed to the presence of an impurity (∼5%) consisting of the singly functionalized dyad that bears no immediate impact on the results presented herein (see the Supporting Information for additional details and discussion).

**Figure 2:**
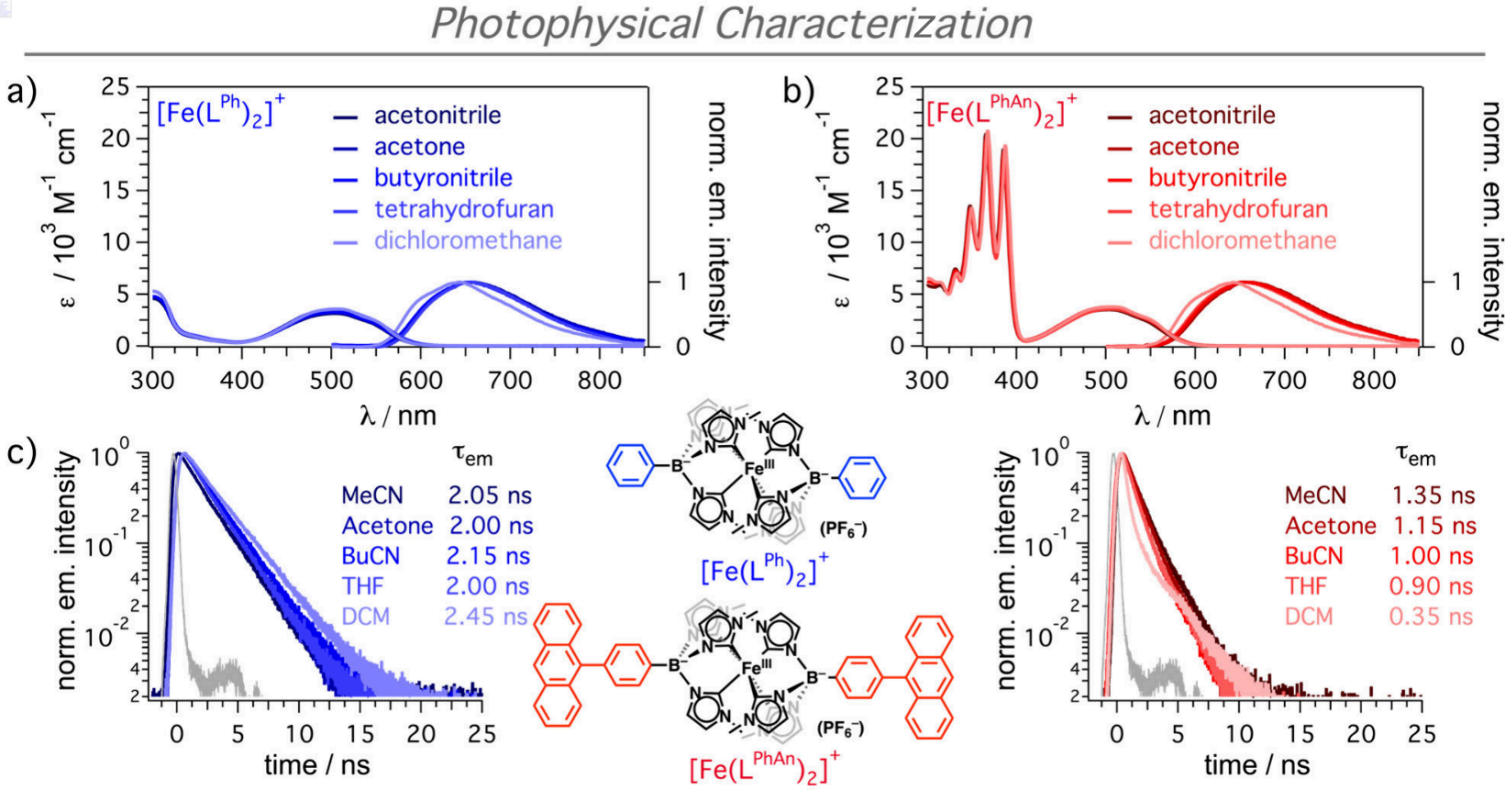
UV–vis absorption spectra and steady-state emission spectra of [Fe­(L^Ph^)_2_]^+^ (a, blue) and [Fe­(L^PhAn^)_2_]^+^ (b, red) in different solvents. (c) Time-resolved emission measured by time-correlated single photon counting (TCSPC) upon excitation at 510 nm, monitored at the corresponding emission maximum in the respective solvent. The color-code for the photocatalysts in different solvents is indicated in (a) and (b). The instrumental response function is presented in gray. Further details are presented in section 2.1 of the Supporting Information (SI).

### Fs-TA Spectroscopy using 500 nm Excitation

The ultrafast excited-state behavior of [Fe­(L^Ph^)_2_]^+^ and [Fe­(L^PhAn^)_2_]^+^ was first studied in MeCN, under excitation at 500 nm, and analyzed by target analysis (Figure 3).^22,23^ For [Fe­(L^Ph^)_2_]^+^ two main excited-state absorption (ESA) bands were detected with maxima at 360 and 575 nm (SI section 3.2.3). They decayed with an excited-state lifetime of 2.3 ns, which matches the 2.05 ns obtained via TCSPC. No other significant spectral changes were observed. This is in line with previous reports by Yartsev, Lomoth, Persson, Wärnmark and co-workers and is assigned to the ^2^LMCT.^9,24^ The expected ground-state bleach around 500 nm is not visible due to the intense ESA. For [Fe­(L^PhAn^)_2_]^+^, spectral evolution included one exponential decay and an infinite offset, which were fitted by using a sequential model (see SI section 3.2). Target analysis afforded an initial excited state with a lifetime of 1.4 ns in MeCN (Figure 3a and Figure S14) and a species-associated differential spectrum (SADS) corresponding to ^2^LMCT, in line with the 1.35 ns lifetime obtained from time-correlated single photon counting experiments.^15^ A novel ESA assigned to the ^3*^PhAn was observed at 430 nm (Figure 3a and SI section 3.2.3), which appeared hand in hand with the decay of ^2^LMCT (inset of Figure 3a) and persisted for the whole time scale of our fs-TA experiment. At first glance, this observation is in agreement with a direct doublet-triplet energy transfer between the iron photosensitizer and the attached triplet acceptor. No other deactivation pathways, such as electron transfer products of the reduced iron center and oxidized anthracene ([(L^PhAn^)­Fe^II^(L^PhAn•+^)]^+^), were detectable in MeCN upon excitation with green light.

**Figure 3:**
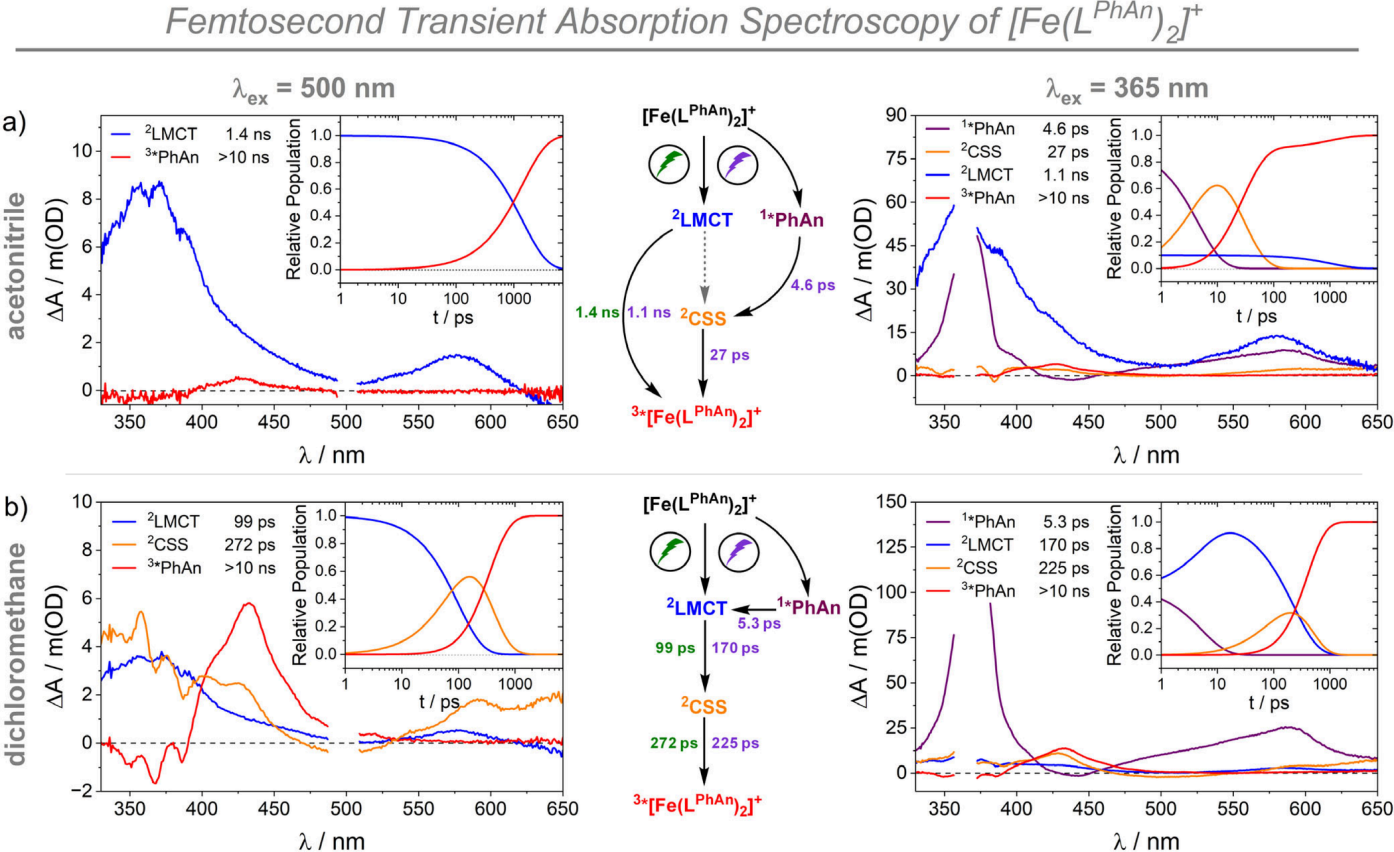
Species-associated differential spectra obtained by target analysis of [Fe­(L^PhAn^)_2_]^+^ in (a) MeCN and (b) DCM upon excitation at 500 nm (left column) and 365 nm (right column). The corresponding relative populations of the individual excited states are shown as the inset of the respective figure section. The color-code for the individual excited states is presented as an inset and matches the scheme in the middle. Electronic configurations of the individual steps are shown later (*vide infra*). The final triplet excited state which is considered as an anthracene-centered triplet state is abbreviated as ^3*^PhAn in (a) and (b) instead of ^3*^[Fe­(L^PhAn^)_2_]^+^. Further spectroscopic details, figures, and models for global fitting are presented in the SI (section 3.2).

As described before, in DCM, the lifetime of the ^2^LMCT decreased to 0.35 ns and the photoluminescence quantum yield decreased to 0.50% (Table S2). This is significantly smaller than those of the unmodified complex (2.45 ns, 2.22%) and the dyad in MeCN (1.35 ns, 1.33%). We thus performed fs-TA experiments in DCM to get further insights, as excited-state energies of charge-separated states and quenching pathways are sensitive to the solvent.^25–31^ Inspection of the kinetic traces (Figure S15) revealed spectral changes consistent with two exponential decays and an infinite offset. An all-sequential model was used to fit the data. The initially populated state, with a 99 ps lifetime, featured ESAs at 360 and 575 nm, hence corresponding again to the ^2^LMCT (blue traces in Figure 3b). Only at much longer time-scales, the ^3*^PhAn populated in [Fe­(L^PhAn^)_2_]^+^ was clearly detectable at 430 nm (red trace in Figure 3b), in line with the characteristic spectroscopic features also visible for ^3*^PhAn generated by triplet–triplet energy transfer via bimolecular quenching of [Ru­(bpy)_3_]^2+^ (an assignment of the observed species is presented in Figure S12). This state persisted for over 10 ns, in line with the long-lived dark excited state observed by ns-TAS (SI section 3.4.1). Surprisingly, next to these expected spectroscopic features, additional peaks at 592 and 650 nm were observed at intermediate time scales (orange traces in Figure 3b and Figure S15). Remarkably, the formation of this short-lived intermediate state was correlated to the decay of ^2^LMCT, and its decay on a longer time scale went hand in hand with the formation of the triplet excited state (inset of Figure 3b). This revealed that, in DCM, the dyad does not populate the triplet excited state of the polyaromatic hydrocarbonby direct DTET, but rather via an intermediate state (Figure 3). Our interpretation based on a sequential model is chemically meaningful, as comparison with spectroelectrochemical reference spectra (SI section 3.2.1) allows for a confident assignment of the intermediate state as the intramolecular charge-separated state (^2^CSS, {[(L^PhAn^)­Fe^II^(L^PhAn•+^)]}^+^) occurring through intramolecular reductive quenching of the excited iron complex (orange traces in Figure 3b). This quenching step leads to the formation of the oxidized PhAn moiety with absorption maxima at 400 nm, 430 and 590 nm and a reduced iron center with a maximum centered around 360 nm and a weak GSB at 500 nm. Hence, based on these spectroscopic findings we propose a mechanism of ^2^LMCT → ^2^CSS → ^3*^PhAn for the population of the desired triplet state (Figure 3b). Similar mechanisms consisting of excited-state electron-transfer quenching followed by charge-recombination to populate a state with a different local spin multiplicity compared to the initial excited state are known in the literature.^32–34^ Details about the spin states of the different intermediates are presented later (*vide infra*). From an energetic viewpoint, this sequence is downhill in DCM as the ^2^CSS (∼1.90 eV, *vide infra*, solvent influence section) is energetically in between the ^2^LMCT (2.15 eV, Table S1) and ^3*^PhAn (∼1.8 eV).^17,35^

A drastic change from a direct DTET mechanism in MeCN, to an electron-transfer and charge-recombination mechanism in DCM seems quite surprising, as the latter is thermodynamically also feasible in MeCN. Thus, an alternative mechanism involving the population of an intermediate ^2^CSS in MeCN was also considered and investigated. As the charge-recombination step to populate ^3*^PhAn in DCM occurs with a time constant of 272 ps (Figure 3b), we speculated that a similar electron-transfer cascade was also operative in MeCN, but the slow ^2^LMCT quenching prevented the detection of the short-lived intermediate. To elucidate this hypothesis, we performed fs-TA experiments under 365 nm excitation.

### Fs-TA Spectroscopy under 365 nm Excitation

The anthracene-dominated absorption features in the UV range of the absorption spectra (Figure 2b) were excited by using a 365 nm pump. The contribution of PhAn ground-state absorptions between 350 and 390 nm (Figure 2a and **b**) allowed an almost exclusive excitation (>90%) of the polyaromatic hydrocarbon.^15^ In line with these expectations, excitation at 365 nm in MeCN led predominantly to the spectroscopic signature of ^1*^PhAn around 370 nm directly observed after the laser pulse, together with minor contributions from the ^2^LMCT (Figure 3a and Figure S16). Hence, a target model involving two parallel branches (Figure 3) was employed to fit the data. In the first branch, initially populated ^2^LMCT feeds ^3*^PhAn with a lifetime of 1.1 ns, consistent with the value obtained upon 500 nm excitation. In the second branch, remarkably, we were able to resolve the intermediate ^2^CSS. In fact, the initially populated ^1*^PhAn is converting to the intermediate ^2^CSS with a lifetime of 4.6 ps. Here, ^2^CSS is short-lived and leads to the formation of ^3*^PhAn with a lifetime of 27 ps and this ^3*^PhAn then persists for more than 10 ns. Importantly, no ISC from ^1*^PhAn to ^3*^PhAn was detectable. While ISC is between 25% and 40% for PhAn alone (Figures S18 and S19), the comparably slow process (τ ≈ 5 ns) is orders of magnitude slower than the processes observed by fs spectroscopy. As a consequence, electron transfer processes outcompete the ISC, assuming no change between the PhAn unit in the dyad and free PhAn (SI section 2.1.3). Hence, the population of ^3*^PhAn via ISC from ^1*^PhAn can be considered as negligible. Also, there is no increase in the ^2^LMCT population at early time scales, confirming that Förster resonance or Dexter energy transfer mechanisms are negligible processes under these conditions. Instead, electron transfer deactivation pathways from ^1*^PhAn largely dominate after excitation of anthracene-based absorption. This is consistent with a highly exergonic (ΔG ≈ –1.16 eV) oxidative quenching of ^1*^PhAn (E*_ox_ ≈ −1.9 V vs SCE) by Fe^III^ (E_red_ = −0.74 V vs SCE). Interestingly, upon predominantly exciting the PhAn moiety within the dyad in DCM at 365 nm (Figure 3b and Figure S17), a fast FRET from ^1*^PhAn to ^2^LMCT dominated the decay of the singlet excited state. ^2^LMCT then generated ^2^CSS in 170 ps, which fed ^3*^PhAn in 225 ps, matching our observations under 500 nm excitation.

Notably, ^2^CSS recombination into ^3*^PhAn in 27 ps in MeCN is 1 order of magnitude faster compared to DCM. Thus, in our fs-TA experiments upon 500 nm excitation, formation of ^2^CSS from ^2^LMCT in around 1 ns is 2 orders of magnitude slower than ^2^CSS decay, impeding the detection of ^2^CSS. We therefore postulate that the ^2^LMCT → ^2^CSS → ^3*^PhAn mechanism is operative in both MeCN and DCM.

### Solvent Influence on Ultrafast Excited-State Dynamics

To understand the origin of the difference observed between solvents, we focused on the energy-level of ^2^CSS as it is expected to have an important impact. Unfortunately, electrochemical measurements were frustrated by irreversible redox chemistry in some solvents (SI section 2.1.1), which prevented the determination of precise redox potentials.^38–43^ As such, only a qualitative rather than quantitative analysis is possible here, with respect to the energy of the charge-separated state estimated from the oxidation potential of the donor and reduction potential of the acceptor. Based on the fs-TA results, it is rational to postulate that the energy of the ^2^CSS is one of the crucial parameters to influence the efficiency of the triplet state population. In fact, similar to our observations, other chromophores that populate a triplet state via charge-recombination-induced ISC exhibit a highly solvent-dependent behavior,^32–34,44–49^ which is mainly caused by a change of the Coulombic energy in the CSS of the donor and/or acceptor moiety of the chromophore and a subsequent shift of the relative energy of the CSS.^32,50^ Qualitatively, a clear correlation between the emission lifetime from the ^2^LMCT state and the amount of ^3*^PhAn in different solvents is detectable (Table 1). Of course, the competition between charge-recombination to the ground state and charge-recombination to populate the triplet excited state will significantly contribute to the overall efficiency, as discussed later in further detail. We expect that dyads with more reversible oxidation of the polyaromatic compounds might allow, in the future, further insights into the role of the relative energies and rate constants of electron transfer processes within the cascade.

**Table 1:** Fluorescence Lifetimes, CSS Energies, and Gibbs Free Energies for Electron Transfer to Populate CSS and ^3*^PhAn Population Yields of [Fe­(L^Ph^)_2_]^+^ and [Fe­(L^PhAn^)_2_]^+^ in Different Solvents

solvent	ε_r_	τ([Fe(L^Ph^)_2_]^+^)/ns	τ([Fe(L^PhAn^)_2_]^+^)/ns	Φ_ET, 2LMCT_ ^a^	*E*_*CSS*_/eV^b^	ΔG_ET_/eV^c^	Φ(^3*^Fe(L^PhAn^)_2_]^+^)^d^
Acetonitrile	35.9 ^35^	2.05	1.35	0.34	2.09	–0.06	0.05
Acetone	20.7 ^35^	2.00	1.15	0.43	2.04	–0.11	0.20
Butyronitrile	20.3 ^36^	2.15	1.00	0.53	2.04	–0.11	0.25
Tetrahydrofuran	7.58 ^35^	2.00	0.90	0.55	1.86	–0.29	0.50
Dichloromethane	8.93 ^35^	2.45	0.35	0.86	1.90	–0.25	0.75

a Φ_ET,2LCMT_ = 1 – τ­([Fe­(L^PhAn^)_2_]^+^)/τ­([Fe­(^LPh^)_2_]^+^).

b Calculated using eq 1 with *n* = 1; *z*_*A*_ = 1; *z*_*D*_ = 0; *r*_*A*_ *=* 3.2 Å; *r*_*D*_ = 5.4 Å, e = 1.602 × 10^–19^ C; ε_0_ = 8.854 × 10^–12^ CJ^–1^m^–1^; *E*_red,Fe_ = –0.74 vs SCE; *E*_ox,PhAn_= 1.35 V vs SCE and ε_*D*_ = ε_*A*_ = 35.9 in MeCN^35^ (see text for additional details).

c Calculated using eq 4 (see text for additional details).

d Value determined for [Fe­(L^PhAn^)_2_]^+^ by relative actinometry against the bleach of [Ru­(bpy)_3_]^2+^ at 455 nm in MeCN (ε_455 nm_ = 10100 M^–1^ cm^–1^).^37^ The determined values are rounded to values of 0.05. Further details are presented in section 3.4.2 of the SI.

Nevertheless, further insights into the relative energies of the CSS in different solvents can be obtained by using established models. For uncharged donor–acceptor dyads with a charge-transfer-state mediated triplet population, the energy of the CSS normally decreases with larger relative permittivity (ε_r_).^50–52^ This trend is opposite to the trend observable with our dyad (Table 1). The energy of the CSS (*E*_CSS_) in these dyads can be estimated based on electrochemical potentials measured in a given solvent and applying correction factors, termed Born correction (ΔG_s_) and Coulombic workterm (ω) for contributions that occur upon changing the solvent dielectric constant (eqs 1–3).^53^

1 $$E_{CSS}=e\left[E_{ox}-E_{red}\right]+\Delta G_{S}+\omega$$

2 $$\Delta G_{S}=\frac{n\cdot e^{2}}{8\pi \varepsilon_{0}}\left(\frac{2z_{D}+n}{r_{D}}\left(\frac{1}{\varepsilon_{r}}-\frac{1}{\varepsilon_{D}}\right)-\frac{2z_{A}+n}{r_{A}}\left(\frac{1}{\varepsilon_{r}}-\frac{1}{\varepsilon_{A}}\right)\right)$$

3 $$\omega =\frac{n\cdot e^{2}\left(z_{A}-z_{D}-n\right)}{4\pi \varepsilon_{0}\varepsilon_{r}R_{DA}}$$

Thus, to estimate *E*_CSS_,^54–56^ the oxidation potential of PhAn (*E*_ox_) and the reduction potential of the iron center (*E*_red_) were used, in combination with ΔG_S_ that were evaluated in different solvents using eq 2.^57^ As parameters, the number of transferred electrons *n*, the elementary charge *e*, the initial charge of donor *z*_*D*_ and acceptor *z*_*A*_, the dielectric constant ε_r_ of the medium where excited-state electron transfer occurs, the dielectric constant of the solvent where the oxidation ε_D_ and reduction ε_A_ potentials were measured, and the radii of the donor *r*_*D*_ and acceptor *r*_*A*_ are relevant. With the reduction potential (*E*_red,Fe_ = –0.74 V vs SCE) and the oxidation potential (*E*_ox,PhAn_= 1.35 V vs SCE) determined in acetonitrile, the energy of the charge separated state and the Born correction ΔG_S_ can be calculated. In our dyad, this workterm is null and hence not relevant (*vide infra*). The driving force to deactivate the excited state via electron transfer can thus be calculated by using eq 4.

4 $$\Delta G_{ET}=e\left[E_{ox}-E_{red}\right]-E_{0,0}+\Delta G_{S}+\omega$$

With a constant energy *E*_*0,0*_ for the ^2^LMCT of ∼2.15 eV in all solvents, a favorable driving force ranging from –0.06 eV to –0.29 eV for electron transfer is predicted depending on the solvent (Table 1). While the absolute values of *E*_*CSS*_ might not be perfectly accurate, the overall trend between shorter emission lifetimes of ^2^LMCT and a larger driving force for the electron transfer to generate [(L^PhAn^)­Fe^II^(L^PhAn•+^)]^+^ is clearly visible (Table 1). It is worth mentioning that, for simplicity, the three-state model assumes a constant triplet state energy for the 9-phenylanthracene-centered long-lived dark state, which might not be perfectly accurate for all solvents.

Furthermore, dielectric continuum theory (eq 5, where ε_op_ is the squared refractive index) predicts an increase in reorganization energy (λ_o_) as the solvent static dielectric constant is increased.^58,59^ Using this equation, we could estimate reorganization energies that ranged from ∼0.7 eV to ∼1 eV (see SI section 4). Feeding these values into the classical Marcus equation (eq 6), and using typical pre-exponential factor (A) values that ranged from 10^11^ to 10^13^ s^–1^, we were able to determine that the predicted rate for electron transfer was 2 orders of magnitude larger in dichloromethane and tetrahydrofuran than in acetonitrile. These results are in line with the observed quenching and improvement of the triplet formation yield (*vide infra*).

5 $$\lambda_{o}=\frac{e^{2}}{4\pi \varepsilon_{o}}\left(\frac{1}{2r_{D}}+\frac{1}{2r_{A}}-\frac{1}{R_{DA}}\right)\left(\frac{1}{\varepsilon_{op}}-\frac{1}{\varepsilon_{r}}\right)$$

$$k_{et}=A\;\exp\left(-\frac{{\left(\lambda +\Delta G^{{}^{\circ}}\right)}^{2}}{4\lambda k_{b}T}\right)$$ 6

The essentially opposite trend in solvent-dependence for the CSS energies and subsequently the driving forces for electron transfer between neutral dyads and our iron-anthracene dyad can also be understood from eq 1 and 4. A notable difference between the present dyad and neutral organic donor–acceptor molecules with a CSS-mediated triplet state formation lies in the correction factors.^52^ Indeed, in neutral organic donor–acceptor molecules, the Born correction is zero, as both acceptor and donor are neutral (z_D_ = z_A_ = 0), but they become charged after photoinduced electron transfer, which implies a Coulombic workterm that must be considered.^57,60^ This leads to stabilization of the CSS in polar solvents. In contrast, the photoinduced electron transfer in the dyad studied herein is considered a charge-shift reaction, and as such the workterm contribution is zero, but the Born correction must be used. As a consequence, the ^2^CSS within the dyad is stabilized in apolar solvents, as highlighted in Table 1. Following this trend of a solvent-dependent energy of the ^2^CSS, charge recombination is expected to be faster in MeCN compared to DCM, when a similar triplet energy of the PhAn moiety is assumed in both solvents. This matches the experimental results obtained by ultrafast transient absorption spectroscopy where the lifetime of the CSS in acetonitrile is only 27 ps, while it is 1 order of magnitude slower (225–272 ps) in DCM (Figure 3).

### Triplet Formation Yield

Focusing again on the nanosecond time scale, several microseconds of excited-state lifetime are detectable for the dyad in all investigated solvents (SI section 3.3.1). The amplitude of the kinetic trace, or in other words, the concentration of generated triplet, was highly solvent-dependent, ranging from a minimal intensity in MeCN to a large one in DCM (Figure 3). This is unsurprising when comparing the excited-state lifetime of the dyad with the parent [Fe­(L^Ph^)_2_]^+^ (Table 1). A quantification of the triplet yield Φ­(^3*^Fe­(L^PhAn^)_2_]^+^) was performed by relative actinometry using an isoabsorptive solution of [Ru­(bpy)_3_]^2+^ in MeCN.^37^ Triplet formation efficiency only reached 0.05 in a MeCN, whereas it increased to 0.20 in acetone, 0.25 in butyronitrile, and 0.50 in tetrahydrofuran and reached an impressive value of 0.75 in DCM (Figure 4). This quantitative analysis emphasizes again the importance of the relative energy of the LMCT, CSS and final triplet state to allow an efficient population of the long-lived ^3*^[Fe­(L^PhAn^)_2_]^+^ and highlights how the yield can be tuned over 1 order of magnitude. THF represents an obvious outlier, as triplet formation efficiency was lower than anticipated from the *E*_*CSS*_ value. This probably originates from the energy of the ^2^CSS with respect to the ^3*^PhAn energy, which is probably also affected by the change in the solvent and will be investigated in the future.

**Figure 4:**
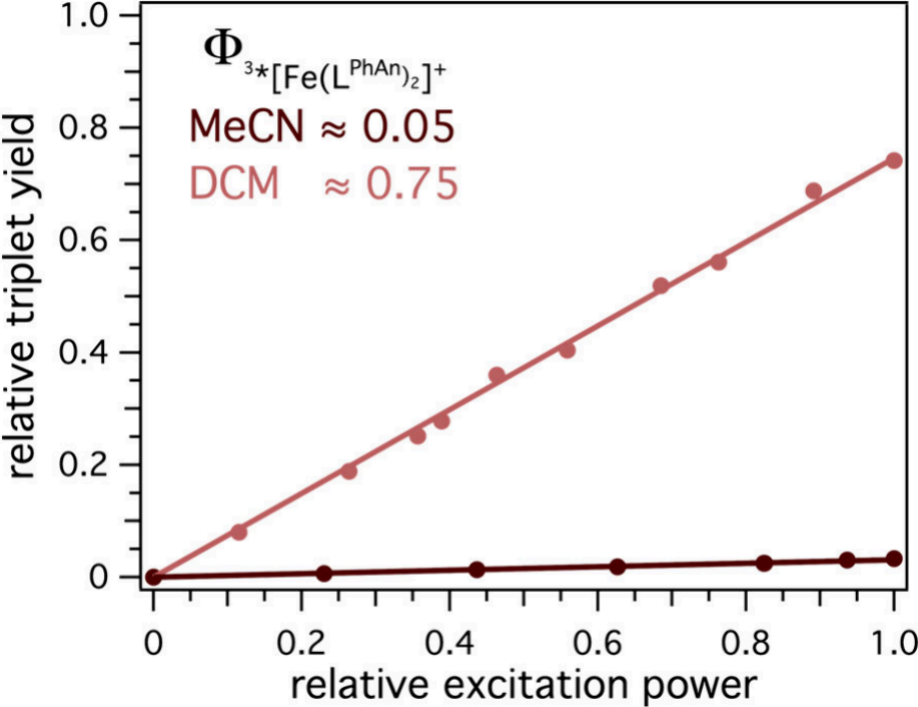
Relative triplet yield Φ­(^3*^Fe­(L^PhAn^)_2_]^+^) with visible light excitation using different excitation power determined by relative actinometry against [Ru­(bpy)_3_]^2+^ in MeCN. Further details are presented in section 3.4.2 of the SI.

An interesting aspect of this analysis is the fact that, in DCM, essentially all of the ∼80% ^2^LMCT excited-state quenching (2.45 ns vs 0.35 ns) results in a long-lived triplet state, while in MeCN, the one-third shorter excited-state lifetime is not reflected in the final triplet yield (0.05). Possible explanations could be (i) an intrinsically different (natural) excited-state lifetime between [Fe­(L^Ph^)_2_]^+^ and [Fe­(L^PhAn^)_2_]^+^, (ii) loss channels that are undetectable in the fs-TAS measurements due to the comparably long lifetime of 1.35 ns of the ^2^LMCT or (iii) an unproductive charge recombination of the ^2^CSS to return to the ground state without population of the triplet state. Gratefully, from the fs-TA analysis in both solvents using 365 nm excitation, where the photons are largely absorbed by the anthracene moiety, the populated singlet state is present for only around 5 ps. This enables a pathway for the formation of the CSS within the dyad different from the one undertaken under 500 nm excitation, providing further insights in both solvents. Thermodynamically, population of ^1*^PhAn (*E*_ox_* = −1.85 V vs SCE) enables an oxidative quenching pathway where ^1*^PhAn is oxidized by the Fe^III^ center (*E*_*1/2,red*_ = −0.74 V vs SCE), leading to the identical ^2^CSS. Interestingly, by relative actinometry in DCM under 355 nm excitation, a triplet yield of 0.72 is determined, which is in great agreement with the triplet yield determined using visible light excitation (0.75). In MeCN only 0.18 of triplet were formed using 360 nm irradiation, pointing toward a significant contribution of unproductive charge recombination in MeCN.

Finally, we were also interested in investigating if bimolecular quenching of the unsubstituted photosensitizer by 9-phenylanthracene could involve a CSS. Recently, Wang and co-workers reported a DTET between [Fe­(L^Ph^)_2_]^+^ and different anthracenes in DMSO for triplet–triplet annihilation upconversion applications.^17^ Bimolecular quenching constants of 10^10^–10^11^ M^–1^s^–1^ exceeding the diffusion limit were reported and interpreted in terms of preassociation. However, in their femtosecond transient absorption data, a charge-separated state was not observed. We hypothesized that the limited solubility of the polyaromatic hydrocarbon in polar solvents would complicate the detection of a potentially short-lived intermediate. Our results, presented in section 3.3 of the SI suggest the possibility that a ^2^CSS is formed upon preassociation and arguably also via diffusional quenching that also mediates, at least partially, the DTET in the bimolecular system, but a detection of the short-lived intermediate is justifiably not possible. Indirect identification upon changing the relative permittivity by the addition of salt (SI section 3.4.3) provided results in line with a ^2^CSS although several inaccuracies complicate an unambiguous interpretation. Thus, at this stage, we are currently unable to definitely ascertain if the diffusional excited-state reaction is occurring via DTET or mediated by the CSS, as small changes within redox potentials, for instance due to a change of the Coulomb interaction between charges to influence the energy of the CSS,^53^ or changes in geometry might be paramount.

### ISC Mechanisms and Spin Considerations

To strengthen our understanding of our dyad and put it into the context of other systems that involve CSS for the population of triplet states, we focused on the observed formal ISC via consecutive steps of electron transfer. When considering the excited-state dynamics of traditionally studied molecular dyads, the initial excited state is usually a singlet state, for instance, in organic donor–acceptor dyads (Figure 5). This is exemplified by a hypothetical dyad based on a metal complex with an electronic configuration of a singlet ground state and a ^1^MLCT excited state (Figure 5a). The overall spin states, electron transfer and intersystem crossing steps, and also the general sequence are similar to organic electron acceptors with closed shell electron configuration in the ground state. In this mechanism, in addition to the overall driving force for each individual step, the ISC rate within the CSS plays a crucial role for the efficient population of the final triplet state.^32,34^ ISC from overall singlet to triplet multiplicity occurs there via spin-flip. Organic donor–acceptor dyads and triads with various combinations of donors and acceptors have been found to combine such electron transfer cascades together with ISC for the population of dark triplet excited states,^51,66–73^ which is sometimes also referred to as radical pair intersystem crossing (RP ISC) or spin–orbit charge-transfer intersystem crossing (SOCT ISC).^34,47,74^ While the latter is especially relevant for small dyads with large electron spin–spin exchange integrals, the RP ISC is observed mainly for systems with large donor–acceptor distances.^54,75,76^ More specifically, donor–acceptor dyads involving anthracene derivatives as electron donors have been reported,^61–65,77^ and usually involve a SOCT ISC mechanism to populate the triplet state. In contrast, examples including dyads of transition metal complexes, where an electron transfer cascade to populate a triplet state is operational, are less common, and mostly reported on ferrocene,^78^ phthalocyanine or porphyrin structures.^44,45,48,79–81^ This type of cascade closely resembles a pathway that plays a key role in biological systems.^82–88^ There, an initially populated singlet state undergoes ISC via intermediate population of a CSS.^32,89^

**Figure 5:**
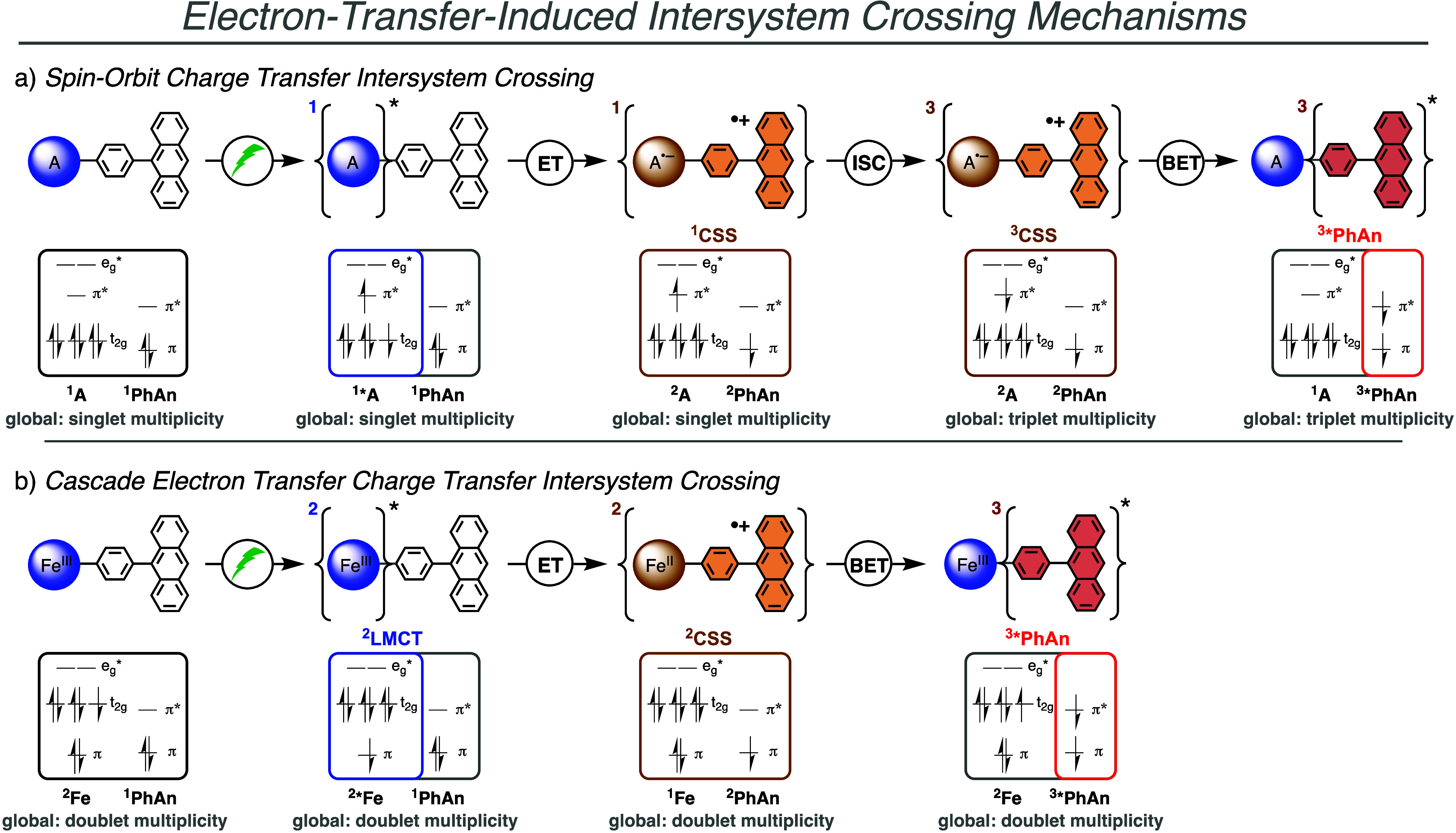
Elementary steps and spin states to populate (local) triplet excited states starting from (a) singlet and (b) doublet charge transfer excited state. Electron configurations are given for O_h_ symmetry for simplicity. A complex with a d^6^ metal configuration and metal-to-ligand charge transfer excited singlet state is assumed in (a) as initial excited state, while common dyads with attached anthracene donors usually rely on organic acceptors attached.^61–65^ For this case, the HOMO would change to a fully filled orbital instead of the t_2g_ configuration from the metal complex, but the main steps, considerations, and spin states of the individual elementary steps remain similar as presented here. The local spin states of the individual electron acceptor (A or Fe) and donor (PhAn) are indicated in bold below the spin configuration while the overall global spin state of the dyad is indicated in gray below the spin configuration. The spin states storing the energy (^2^LMCT, ^1/2/3^CSS and ^3^*PhAn) are indicated above the spin configuration.

However, when the sequence starts from a ground state with an unpaired electron, such as a doublet or quartet state, the situation is different. Starting from a single unpaired electron in the excited state (Figure 5b), as it is the case for the ^2^LMCT of the present dyad, the triplet population on the attached chromophore, possessing a singlet ground state (e.g., ^1^PhAn in Figure 5b), can be accomplished via two consecutive electron transfer steps without any changes in the overall spin multiplicity. While the individual components undergo ISC, the global spin state of the dyad remains in a doublet state throughout the decay cascade. Specifically, the longest-lived state involves a doublet ground-state of the Fe^III^ ion on the one hand, and a (antiferromagnetically coupled) ^3*^PhAn on the other hand (Figure 5b). As a consequence, the oxidation and reduction potentials of the donor and acceptor moieties are paramount parameters that determine the excited-state dynamics of the dyad, while ISC via spin-flip within one electronic state is not relevant. As the overall spin state with respect to the excited state still changes from a (local) doublet state (^2^LMCT) to a (local) triplet state (^3^*PhAn), the overall process is described as a cascade of electron transfer events that do not require ISC. Note that the singlet excited state (^1^*PhAn) will never be formed, as it would be highly endergonic.

Due to the fact that similar electron transfer cascade proceeding though a CSS for the population of (dark) triplet states were not observed in other dyads based on Fe^III^ or doublet excited states,^90–92^ the main factors that govern the mechanism of triplet state population are currently challenging to conclusively rationalize. Presumably, a (mis)­match of redox potentials is one of the major parameters to enable or prevent the electron transfer cascade from populating the triplet state, but further investigations are needed to provide a deeper and unifying understanding of these processes. Nevertheless, our study unambiguously demonstrates an unusual pathway to populate a (dark) triplet excited state and highlights that an in-depth (spectroscopic) analysis allows us to provide valuable insights critical for a rational design of the next generation of iron-based dyads.

## Conclusions

In conclusion, fs-TA spectroscopy revealed a previously unreported mechanism to populate a polyaromatic hydrocarbon’s triplet state in earth abundant photosensitizers. Direct DTET is not occurring, and the triplet state is exclusively populated from a charge-separated state generated via intramolecular electron transfer. This mechanism opened a straightforward way to control the final triplet yield by tuning the solvent polarity. As such, triplet yield could be tuned from 5% in MeCN to 75% in DCM, an observation that was directly correlated with the relative energy of the charge-separated state.

This report bears immediate and clear impact for the design of molecular dyads based on earth abundant photosensitizers, where both the energy states of the photosensitizer/energy acceptor pair and their respective redox potentials must be taken into consideration to enable the successful population of the desired long-lived dark triplet state. Whereas typical dyad based on d^6^ electronic configuration often proceed via intersystem crossing and Dexter energy transfer processes, the d^5^ configuration of the iron center channels this novel pathway that proceeds exclusively via a cascade of electron transfer, without the need for a formal overall spin change, thus rendering the triplet population path more efficient. We believe that such processes will also be discovered in the coming future, with photosensitizers exhibiting an electronic configuration different from the classical d^6^ one.

Furthermore, an unexpected clear difference between the excited-state deactivation of the iron-based ^2^LMCT state, within the dyad and upon bimolecular quenching by PhAn, was detected, which presumably arises from geometric and electronic aspects or short-lived undetectable intermediates. This might also play a role for other dyads where electron and energy transfer processes are feasible, and thus, changes between intramolecular and intermolecular pathways should be investigated. A more detailed study to understand these aspects and develop a rational design of the dyads will follow.

In the bigger context, our work complements other results of photophysical studies of first-row transition metals that have uncovered unique pathways that were not visible with precious metal-based analogues.^8,93–98^ The observed mechanism investigated here might be more relevant for other complexes based on earth-abundant (first-row) transition metals to utilize these new pathways for the formation of long-lived exited states. The discovery of this unique pathway compared to the breadth of reports on direct energy transfer is most probably a consequence of: (i) the efficient decoupling between the iron core and the anthracene moiety, (ii) the energy level of the ^2^CSS that is located between the energy level of the excited photosensitizer and ^3*^PhAn and (iii) the orientation/geometry of the final energy acceptor compared to the iron center. With these aspects in mind, it is now possible to judiciously design dyads where electron transfer and energy transfer processes could be straightforwardly “switched on/off” based on the relative energy of the involved states.

## Supplementary Material





## Notes

Experimental details, spectroelectrochemical characterization, additional spectroscopic characterization (PDF)
Transparent Peer Review report available (PDF)
