# Supplementary material for: Beyond Common Energy Transfer: Intramolecular Electron Transfer Cascade Controls Triplet Population of a Long-Lived Iron-Anthracene Molecular Dyad
Source: ACS Cent Sci. 2025 Aug 4;11(10):1870–81. doi: 10.1021/acscentsci.5c01040 (PMC12550628; doi:10.1021/acscentsci.5c01040)
Supplement: Supplementary file 1 [file oc5c01040_si_001.pdf]

Beyond Common Energy Transfer: Intramolecular Electron Transfer Cascade Controls Triplet Population of a Long-Lived Iron-Anthracene Molecular Dyad

Felix Glaser,[‡,*] Giovanni M. Beneventi,[δ, ±] Alejandro Cadranel,[δ, ±, Σ, ⊥*] and Ludovic Troian-Gautier[‡,ϕ*]

[‡] UCLouvain, Institut de la Matière Condensée et des Nanosciences (IMCN), Molecular Chemistry, Materials and Catalysis (MOST), Place Louis Pasteur 1/L4.01.02, B-1348 Louvain-la-Neuve, Belgium

[δ] Friedrich-Alexander-Universität Erlangen-Nürnberg (FAU), Physical Chemistry I, Egerlandstr. 3, 91058, Erlangen, Germany.

[±] Friedrich-Alexander-Universität Erlangen-Nürnberg (FAU), Interdisciplinary Center for Molecular Materials, Egerlandstr. 3, 91058, Erlangen, Germany.

[Σ] Universidad de Buenos Aires, Facultad de Ciencias Exactas y Naturales, Departamento de Química Inorgánica, Analítica y Química Física, Pabellón 2, Ciudad Universitaria, C1428EHA, Buenos Aires, Argentina.

[⊥] CONICET – Universidad de Buenos Aires, Instituto de Química-Física de Materiales, Medio Ambiente y Energía (INQUIMAE), Pabellón 2, Ciudad Universitaria, C1428EHA, Buenos Aires, Argentina.

[ϕ]Wel Research Institute, Avenue Pasteur 6, 1300 Wavre, Belgium

Correspondence to: Ludovic.Troian@uclouvain.be, Felix.Glaser@uclouvain.be, ale.cadranel@fau.de

Table of Contents

1.	*General Experimental Details*	*S3*
2.	*Additional Characterization*	*S7*
2.1.	*Photophysical Characterization*	*S7*
2.1.1.	*(Spectro)electrochemical Characterisation*	*S10*
2.1.2.	*Molar Absorption Coefficient of ^{3*}PhAn in Different Solvents*	*S15*
2.1.3.	*Intersystem Crossing Quantum Yield of ^{1*}PhAn in Different Solvents*	*S17*
3.	*Transient-Absorption Spectroscopy*	*S18*
3.1.	*Spin States and Overview of Possible Mechanistic Scenarios*	*S18*
3.2.	*Femtosecond Transient-Absorption Spectroscopy*	*S19*
3.2.1.	*Spectral Assignment of Species-Associated Differential Spectra*	*S19*
3.2.2.	*Fit Model for Target Analysis of fs-Transient Absorption Datasets*	*S20*
3.2.3.	*fs-TA-Spectroscopy of $[Fe(L^{PhAn})_2]^+$ Excited at 500 nm*	*S21*
3.2.4.	*fs-TA-Spectroscopy of $[Fe(L^{PhAn})_2]^+$ Excited at 365 nm*	*S22*
3.2.5.	*fs-TA-Spectroscopy of PhAn Excited at 365 nm*	*S23*
3.3.	*Bimolecular Quenching of $[Fe(L^{Ph})_2]^+$ by PhAn*	*S24*
3.3.1.	*fs-TA-Spectroscopy of $[Fe(L^{Ph})_2]^+$ in the Presence of PhAn Excited at 500 nm*	*S26*
3.3.2.	*fs-TA-Spectroscopy of $[Fe(L^{Ph})_2]^+$ Excited at 500 nm*	*S28*
3.4.	*Nanosecond Transient-Absorption Spectroscopy*	*S29*
3.4.1.	*ns-TA-Spectroscopy: $^{3*}[Fe(L^{PhAn})_2]^+$ Lifetimes*	*S29*
3.4.2.	*ns-TA-Spectroscopy: $^{3*}[Fe(L^{PhAn})_2]^+$ Formation Quantum Yields*	*S31*
3.4.3.	*ns-TA-Spectroscopy: Salt-Dependent ^{3*}PhAn for Bimolecular Quenching*	*S35*
4.	*Additional Calculations*	*S36*
5.	*Bibliography*	*S37*

1. General Experimental Details

Solvents and Chemicals. Dry acetonitrile 99.8% (Sigma-Aldrich), acetone ≥99.8% (VWR Chemicals), dichloromethane 99% (VWR), 9-phenylanthracene 98% (BLDPharm), 9,10-diphenylanthracene 98% (Ambeed), potassium nitrate 99% (Acros Organics), were purchased from commercial suppliers and used as received. Water was purified by a Millipore Milli-Q system. Anhydrous tetrahydrofuran (stabilised with BHT, >99.5%, TCI) and butyronitrile (>99%, TCI) were distilled prior to use.

Synthesis. The dyad and undecorated iron complex were synthesized following published procedures.[1] While ^{1}H-NMR and elemental analysis indicate a pure dyad $[Fe(L^{PhAn})_2](PF_6)$, kinetic emission measurements in dichloromethane, where the excited state is quenched with ~80% efficiency based on the remaining ^{2}LMCT emission, led to the observation of a longer-lived component (~2 ns, see section 2). Different additional purification approaches including column chromatography and size exclusion chromatography were not successful to remove the iron-based impurity, which is estimated to account for less than 5% and is attributed to a mono-functionalized complex with only one anthracene moiety attached based on analytical HPLC measurements. We hypothesize that the LMCT excitation of that photosensitizer is split between 50% from the ligand bearing the anthracene moiety (probably leading to the desired ^{3*}PhAn unit) and 50% from the unfunctionalized ligand, leading to the standard 2 ns photoluminescent lifetime of the photosensitizer. Taking all spectroscopic data and analysis into account, the impurity is not expected to have a noticeable influence on the findings and conclusions of our study. Noteworthy, for the analysis of the emission lifetime with reconvolution fit and a biexponential decay, the shorter component was assigned to the dyad, which is in line with the lifetimes obtained by global analysis of the femtosecond data, and the longer component was assigned to mono-functionalized complex. An excited-state equilibrium to repopulate the emissive state to allow delayed emission cannot be ruled out,[2–8] but this is expected to be less relevant due to the energy difference between the triplet state and ^{2}LMCT.

UV-Visible Absorption. UV−vis absorption spectra were recorded on an Agilent Cary 60 spectrophotometer in a quartz cuvette with a 1 cm path length. Molar absorption coefficients were determined in at least three independent measurements with a less than 5% standard deviation.

Electrochemistry. Cyclic voltammetry was performed with an Autolab PGSTAT 100 potentiostat using a standard three-electrode-cell, i.e., a glassy carbon disk working electrode

(approximate area = 3 mm^2), a platinum wire counter electrode, and an aqueous Ag/AgCl reference electrode (salt bridge: 3 M KCl/saturated AgCl). Experiments were performed in dry acetonitrile or dry DMF with 0.1 M $TBAPF_6$ as electrolyte at a scan rate of 100 mV s^{-1}, unless otherwise stated. The sample, with a complex concentration of 0.5 mM, was purged with argon before the measurement. For comparison purposes, the electrochemical potentials were converted to SCE by subtracting 0.045 V.

Spectroelectrochemistry. Spectroelectrochemistry measurements were carried out in an OTTLE cell. The cell consists of a demountable cell body equipped with a set of CaF_2 optical windows (41×23×6 mm) allowing to perform UV-Vis-NIR-IR (down to 1100 cm^{-1}) spectroelectrochemical experiments. The windows are separated with a modified polyethylene spacer (optical path of ~0.2 mm) with a melt-sealed three-electrode system consisting of a Pt minigrid working electrode (32 wires/cm), a Pt minigrid auxiliary electrode and an Ag wire pseudo-reference electrode. The electrode set is housed in a solvent-resistant polyoxomethylene (POM) frame. The wire contacts are stable against air oxidation and shielded from electronic noise. Electrochemical potential was controlled using an EmStat4S LR potentiostat from Palmsens. UV-Visible spectra at different applied potentials were recorded on the previously described Agilent setup.

Time-Resolved and Steady-State Photoluminescence. Time-resolved and steady-state photoluminescence spectra were recorded on an Edinburgh Instruments FS5 Spectrofluorometer equipped with a time-correlated single photon counting module. The steady-state photoluminescence spectra were recorded using a 150 W Xenon arc lamp as the excitation source. The photoluminescence was detected at a right angle to the excitation beam using a single photon counting PMT-900 in a temperature stabilized housing. The spectra were integrated at 0.2 s and three spectra were averaged. Steady-state photoluminescence spectra were corrected for the instrument's spectral response. All room-temperature spectra were obtained from Argon-purged solutions unless otherwise mentioned.

Time-resolved photoluminescence data were collected on the FS5 using the time-correlated single photon counting technique (TCSPC). Excitation was achieved with a 510 ± 5 nm diode laser (Edinburgh Instruments EPL-510, 90 ps pulse width at 10 MHz). Photons reaching the detector were usually accumulated to reach a count of 10000. The kinetic data were analysed with the software of the FS5 using a reconvolution fit taking the instrumental response function (IRF) into account.

Nanosecond UV-Visible Transient Absorption Spectroscopy. Nanosecond transient absorption (ns-TA) measurements were recorded on a LP980-K spectrometer from Edinburgh

Instruments equipped with an iCCD detector from Andor (DH320T). The excitation source was a tunable Nd:YAG Laser NT342 Series from EKSPLA. The third harmonic (355 nm) at 150 mJ was directed into an optical parametric oscillator (OPO) to enable wavelength tuning starting from 410 nm. The laser power was then attenuated to reach appreciable signal/noise and the integrity of the samples was verified by UV-vis measurements. The LP980-K is equipped with a symmetrical Czerny-Turner monochromator. For single wavelength absorption changes, a 1800 g mm^{-1} grating, blazed at 500 nm is used, which affords wavelength coverage from 200 to 900 nm. For spectral mode (iCCD), a 150 g mm^{-1} grating, blazed at 500 nm is used, offering a wavelength coverage of 540 nm over the full wavelength range extending from 250 to 900 nm. Single wavelength absorption changes were monitored using a PMT LP detector (Hamamatsu R928) which covers the spectral range from 185 to 870 nm. The probe was a 150 W ozone-free xenon short arc lamp (OSRAM XBO 150W/CR OFR) that was pulsed at the same frequency of the laser. All measurements were performed in argon-purged solution at room temperature. An average of 10 to 50 scans per measurement was used. All solutions were purged with argon for 10-15 minutes unless otherwise stated.

Femtosecond UV-Visible Transient Absorption Spectroscopy. Transient absorption experiments were conducted using an Astrella-F-1K amplified Ti:sapphire femtosecond laser system from Coherent, operating at a repetition rate 1kHz, 5.5 W power (5 mJ pulse energy), with a pulse duration of 80 fs. TA pump/probe Helios detection system from Ultrafast Systems was utilized. In the fsTA experiment, an optical delay line placed in the probe beam pathway allowed for time delays up to 7.2 ns. White light in the UV and visible regions of the optical spectrum (~350-650 nm) was generated focusing a fraction of the fundamental 800 nm output onto a 3 mm CaF_2 crystal mounted on a motorized stage. A 1.2 mJ fraction of the fundamental was used for pump beam generation by a TOPAS Prime from Light Conversion with standard NirUVis extension. Pump energy was typically 1000 nJ. A depolarizer was placed in the pump beam to avoid rotational dynamics. Bandpass filters with ± 5 or ± 10 nm were used to ensure low spectral width and to exclude 800 nm photons. Typical excitation spot diameters are around 150-200 μm and ensured to be larger than those of the probe beam. All measurements were conducted in a 2 mm quartz cuvette under argon atmosphere, using solutions with room temperature absorbances of 0.5-0.7. To analyze transient absorption data, we used a suggested procedure.[9] We start with SVD and global analysis, using an all-sequential decay model that provides evolution associated spectra of potentially intervening species, to determine the number of decaying species that participate in the decay cascade. However, this doesn't necessary yield differential spectra with genuine physicochemical meaning. Afterwards, a

target analysis is applied, using specific target models that result in species associated spectra with true physicochemical meaning. Obtained data were treated by SVD, global and target analyses using the R- package TIMP and GloTarAn.[9–11] The instrument response function (IRF) and dispersion (chirp of the white light pulse) were modelled and taken into account during the fitting procedure. Note that the relative populations does not account for unproductive return to the ground state from intermediates i.e. the overall sum of relative population always equals 1. Fit models for target analysis are shown in section 3.2.2.

2. Additional Characterization

2.1. Photophysical Characterization

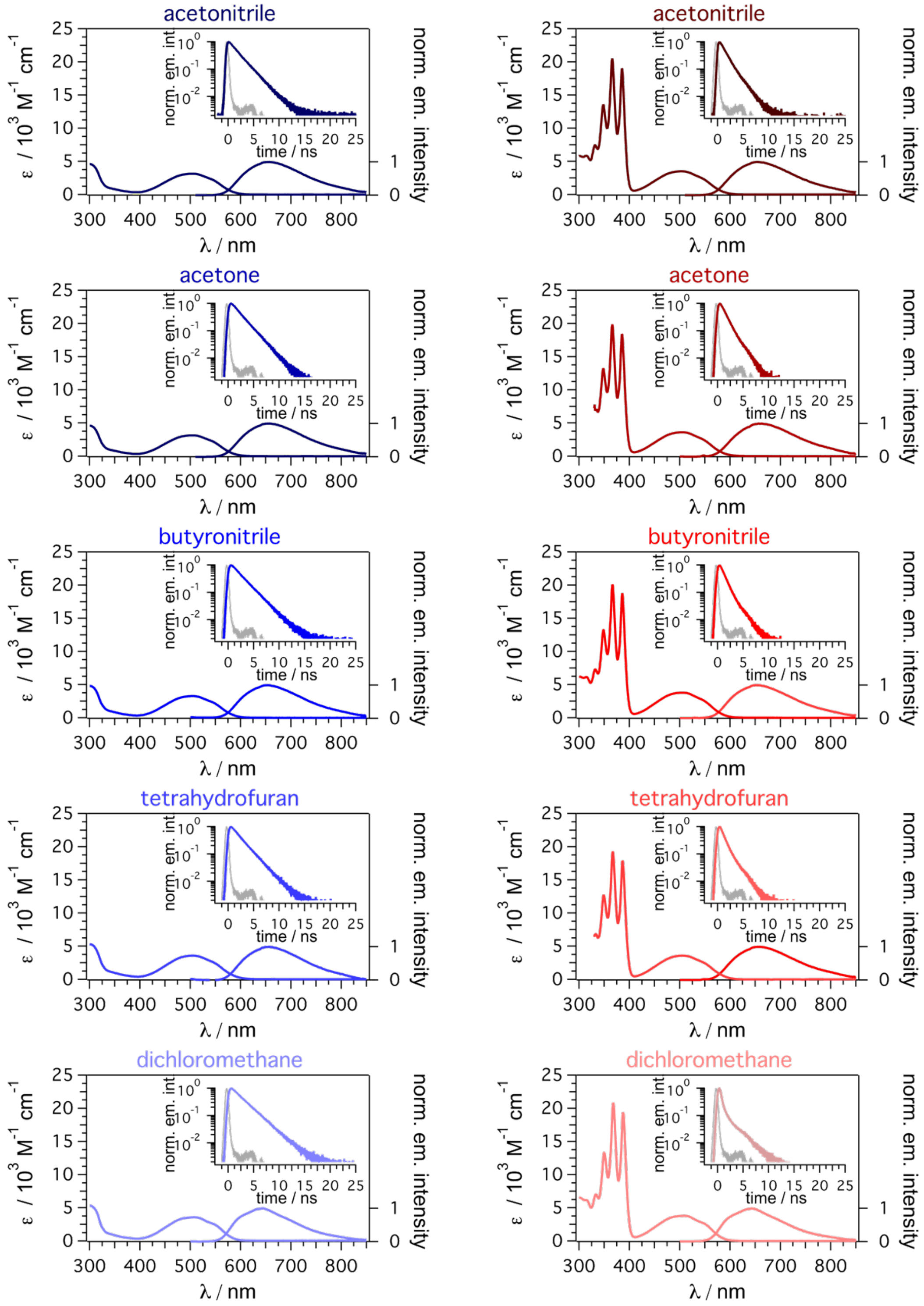

Figure S1. UV-vis absorption and emission spectra as well as emission lifetimes of $[Fe(L^{Ph})_2]^+$ (blue, left column) and $[Fe(L^{PhAn})_2]^+$ (red, right column) recorded in different solvents. The same data is presented in Figure 2 of the main manuscript in one stacked graph. Emission lifetimes were recorded at the emission maximum and the instrumental response function is provided in grey.

Table S1. Overview of the photophysical properties of **[Fe(L^{Ph})$_2$]$^+$** recorded in different solvents.

**solvent**	$\lambda_{em,max}$ / **nm**	$\Phi_{em,air}$ / % [a]	$\Phi_{em,Ar}$ / % [a]	$\tau_{em,air}$ / **ns**	$\tau_{em,Ar}$ / **ns**	$E_{0.0}$ / **eV** [b]
Acetonitrile [c]	656	1.66 ±0.04	1.75 ±0.07	2.05	2.05	2.14
Acetone	655	1.87 ±0.01	1.83 ±0.02	2.00	2.00	2.14
Butyronitrile	655	1.87 ±0.05	1.81 ±0.08	2.00	2.00	2.14
Tetrahydrofuran	656	1.92 ±0.03	1.92 ±0.03	2.15	2.15	2.14
Dichloro-methane	643	2.21±0.08	2.22±0.03	2.45	2.45	2.15

Emission lifetimes were rounded to 50 ps. [a] Photoluminescence quantum yields of [Fe(L^{PhAn})$_2$]$^+$ were determined relative to an aerated solution of [Ru(bpy)$_3$](PF$_6$)$_2$ in acetonitrile (ϕ = 0.018).[12] Changes between air and Argon are within the general estimated of error of 5-10% within these measurements. [b] Emission excited-state energies $E_{0.0}$ were estimated based on the energy at the intersection of the normalized emission and absorption spectra. [c] These values were reported in another study.[1]

Table S2. Overview of the photophysical properties of **[Fe(L^{PhAn})$_2$]$^+$** recorded in different solvents.

**solvent**	$\lambda_{em,max}$ / **nm**	$\Phi_{em,air}$ / % [a]	$\Phi_{em,Ar}$ / % [a]	$\tau_{em,air}$ / **ns** [b]	$\tau_{em,Ar}$ / **ns** [b]	$E_{0.0}$ / **eV** [c]
Acetonitrile [d]	655	1.14 ±0.02	1.17 ±0.01	1.35	1.35	2.14
Acetone	658	1.10 ±0.03	1.12 ±0.02	1.15	1.15	2.14
Butyronitrile	650	0.90±0.06	0.92±0.04	1.05	1.00	2.14
Tetrahydrofuran	655	0.86±0.07	0.86±0.06	0.90	0.90	2.14
Dichloro-methane	642	0.49±0.03	0.50±0.03	0.35	0.35	2.15

Emission lifetimes were rounded to values of 50 ps. [a] Photoluminescence quantum yields of [Fe(L^{PhAn})$_2$]$^+$ were determined relative to an aerated solution of [Ru(bpy)$_3$](PF$_6$)$_2$ in acetonitrile (ϕ = 0.018).[12] Changes between air and Argon are within the general estimated of error of 5% within these measurements. [b] For solvents with significant intramolecular excited-state quenching and consequently short emission lifetimes a biexponential emission decay is observed, of which the shorter emission lifetimes is assigned to the [Fe(L^{PhAn})$_2$]$^+$ and the long-lived component with lifetime of ~2 ns is assigned to an iron-based impurity. Further details are provided in the General Experimental Details. [c] Emission excited-state energies $E_{0.0}$ were estimated based on the energy at the intersection of the normalized emission and absorption spectra. [d] These values were reported in another study.[1]

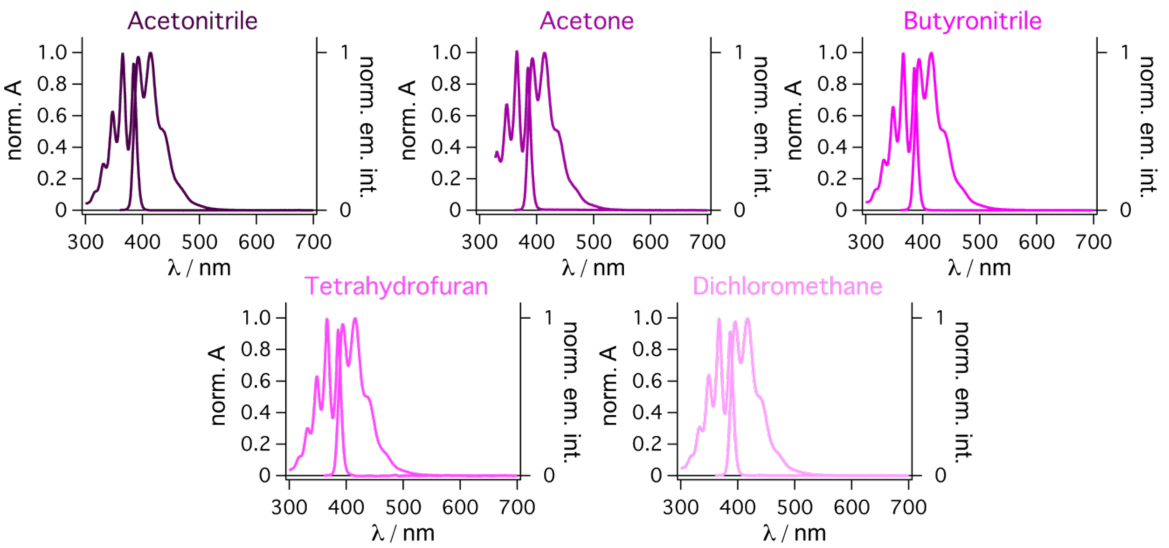

Figure S2. Normalized UV-vis absorption and emission spectra of PhAn in different solvents.

Table S3. Overview of the absorption properties recorded at room temperature in different solvents.

solvent	λ_{abs} (ε) / nm (10^3 M^{-1} s^{-1})		
	**$[Fe(L^{Ph})_2](PF_6)$**	**$[Fe(L^{PhAn})_2](PF_6)$**	**PhAn**
Acetonitrile [a]	299 (4.6), 504 (3.1)	314 (5.9), 332 (7.5), 348 (13.6), 366 (20.5), 385 (19.0), 503 (3.5)	347 (-), 365 (-), 385 (-)
Acetone	505 (3.5)	335 (7.1), 348 (13.2), 366 (19.9), 386 (18.5), 505 (3.6)	347 (-), 365 (-), 385 (-)
Butyronitrile	299 (4.8), 504 (3.3)	315 (6.2), 332 (7.3), 349 (13.3), 367 (20.1), 386 (18.8), 506 (3.8)	348 (-), 366 (-), 385 (-)
Tetrahydrofuran	300 (5.3), 506 (3.6)	314 (5.8), 333 (6.9), 349 (12.7), 367 (19.3), 387 (17.9), 507 (3.6)	348 (-), 366 (-), 386 (-)
Dichloro-methane	300 (5.3), 508 (3.6)	315 (5.7), 334 (7.0), 350 (13.4), 368 (20.8), 388 (19.4), 506 (3.7)	349 (-), 367 (-), 387 (-)

[a] These values were reported in another study.[1]

Table S4. Overview of the intersystem crossing quantum yields of ^{3*}PhAn in different solvents.

**solvent**	**$\lambda_{em,max}$ / nm**	**$E_{0.0}$ / eV** [a]	**$\tau_{1*PhAn,air}$ / ns** [b]	**$\Phi_{ISC,PhAn}$** [c]
Acetonitrile	414	3.20	5.3	0.35
Acetone	414	3.20	- [c]	0.40
Butyronitrile	415	3.20	- [c]	0.35
Tetrahydrofuran	415	3.19	- [c]	0.25
Dichloromethane	417	3.18	4.8	0.30

[a] Estimated from the energy at the crossing point between normalized absorption and emission spectra. [b] Details on excited-state lifetime of ^{1*}PhAn are presented in section 3.2.5, [c] Details on intersystem crossing quantum yield determination are presented in section 2.1.3.

Excitation spectra confirmed that the remaining emission in our dyads are caused by the iron-based emission from the ^{2}LMCT.

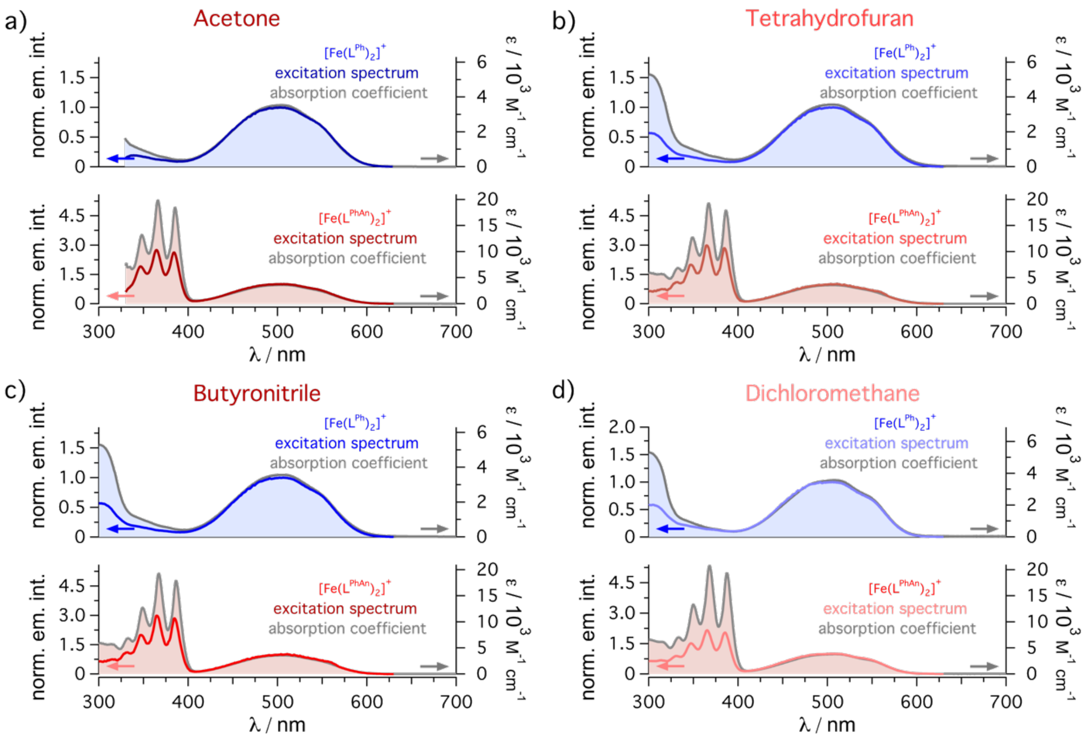

Figure S3. Excitation spectra (dark color) of $[Fe(L^{Ph})_2]^+$ (top, blue) and $[Fe(L^{PhAn})_2]^+$ (bottom, red) in (a) acetone, (b) tetrahydrofuran, (c) butyronitrile and (d) dichloromethane for the excitation-dependent emission intensity were monitored at the respective emission maximum (Table S1, Table S2). The scaled molar absorption coefficients of both complexes are shown for comparison in light grey color.

2.1.1. (Spectro)electrochemical Characterisation

Electrochemical characterisation in acetonitrile of $[Fe(L^{Ph})_2](PF_6)$, $Fe(L^{PhAn})_2](PF_6)$ and PhAn was published in another study.[1]

In all investigated solvents the potentials for $Fe(L^{PhAn})_2](PF_6)$ essentially consist of a combination of the peaks based on the iron complex and the PhAn (**Figure S4** to **Figure S7**). A summary of the redox potentials is presented in **Table S5**. In all investigated solvents expect for dichloromethane (**Figure S7**) the anthracene-based oxidation is non-reversible and the peak maximum is shifting partly with the measurement scan rate. This hampers a proper determination of the oxidation potential and an accurate calculation to estimate the CSS based on electrochemical data, which would be needed to explain the observed photophysical differences (see discussion in the main manuscript). As a consequence, an estimation of the CSS was calculated to explain the observed trend between different solvents.

Electrochemistry in Acetone

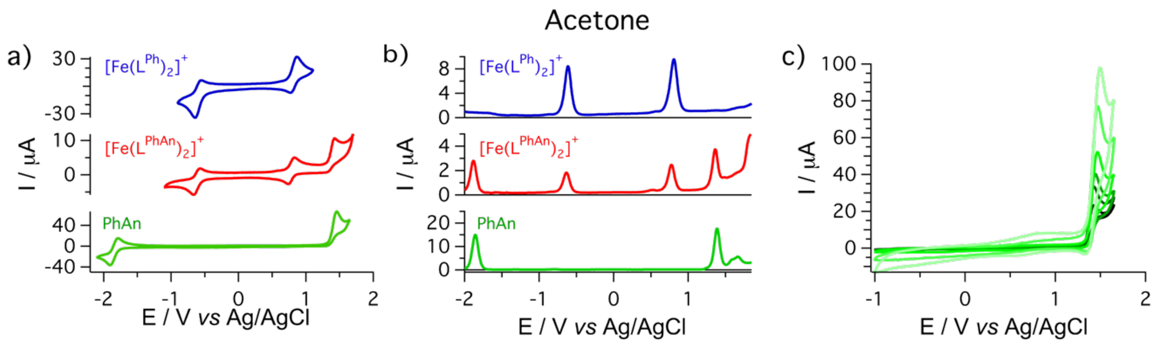

Figure S4. Cyclic voltammetry (a) and differential pulse voltammetry (b) measurements of $[Fe(L^{Ph})_2](PF_6)$ (blue), $Fe(L^{PhAn})_2](PF_6)$ (red) and PhAn (green) in de-aerated **acetone** *vs* Ag/AgCl as reference electrode. c) 9-Phenylanthracene with scan rate of 25 mV s^{-1} to 1000 mV s^{-1}. All solutions contained 0.5-2 mM of redox active species and 100 mM of $TBAPF_6$ as supporting electrolyte. Voltammograms are generally recorded with scan rates of 100 mV s^{-1}. Differential pulse voltammograms were recorded with a scan speed of 20 mV s^{-1}.

Electrochemistry in Butyronitrile

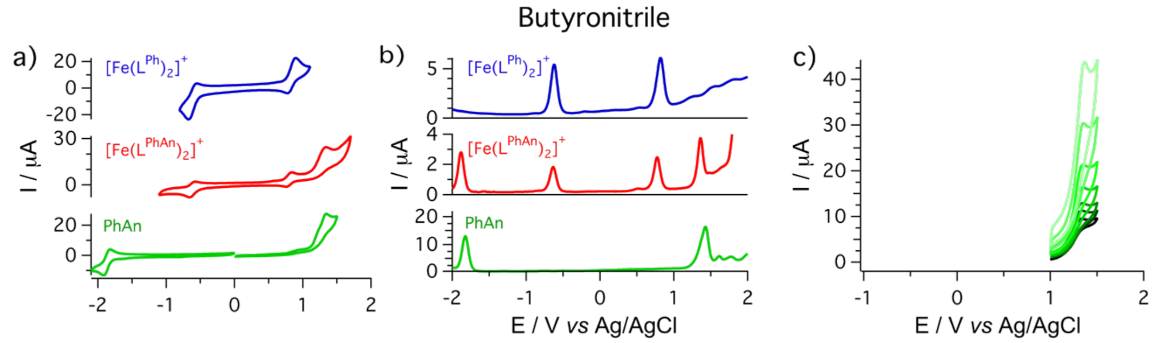

Figure S5. Cyclic voltammetry (a) and differential pulse voltammetry (b) measurements of $[Fe(L^{Ph})_2](PF_6)$ (blue), $Fe(L^{PhAn})_2](PF_6)$ (red) and PhAn (green) in de-aerated **butyronitrile** *vs* Ag/AgCl as reference electrode. c) 9-Phenylanthracene with scan rate of 10 mV s^{-1} to 500 mV s^{-1}. All solutions contained 0.5-2 mM of redox active species and 100 mM of $TBAPF_6$ as supporting electrolyte. Voltammograms are generally recorded with scan rates of 100 mV s^{-1}. Differential pulse voltammograms were recorded with a scan speed of 20 mV s^{-1}.

Electrochemistry in Tetrahydrofuran

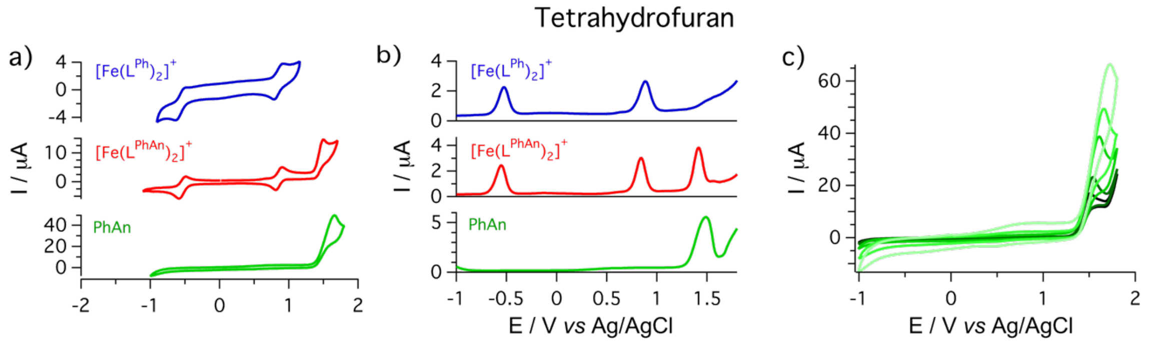

Figure S6. Cyclic voltammetry (a) and differential pulse voltammetry (b) measurements of $[Fe(L^{Ph})_2](PF_6)$ (blue), $Fe(L^{PhAn})_2](PF_6)$ (red) and PhAn (green) in de-aerated **tetrahydrofuran** *vs* Ag/AgCl as reference electrode. c) 9-Phenylanthracene with scan rate of 10 mV s^{-1} to 500 mV s^{-1}. All solutions contained 0.5-2 mM of redox active species and 100 mM of $TBAPF_6$ as supporting electrolyte. Voltammograms are generally recorded with scan rates of 100 mV s^{-1}. Differential pulse voltammograms were recorded with a scan speed of 20 mV s^{-1}.

Electrochemistry in Dichloromethane

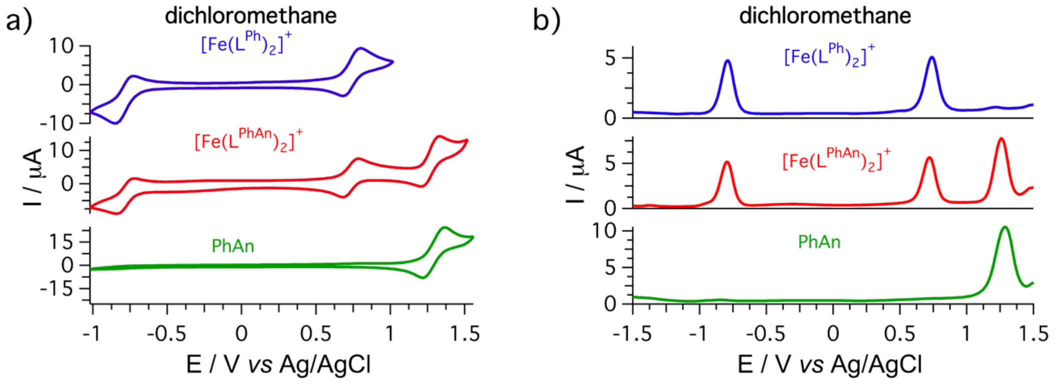

Figure S7. Cyclic voltammetry (a) and differential pulse voltammetry (b) measurements of $[Fe(L^{Ph})_2](PF_6)$ (blue), $Fe(L^{PhAn})_2](PF_6)$ (red) and PhAn (green) in de-aerated **dichloromethane** *vs* Ag/AgCl as reference electrode. All solutions contained 0.5-2 mM of redox active species and 100 mM of $TBAPF_6$ as supporting electrolyte. Voltammograms are generally recorded with scan rates of 100 mV s^{-1}. Differential pulse voltammograms were recorded with a scan speed of 20 mV s^{-1}.

Spectroelectrochemistry in Dichloromethane

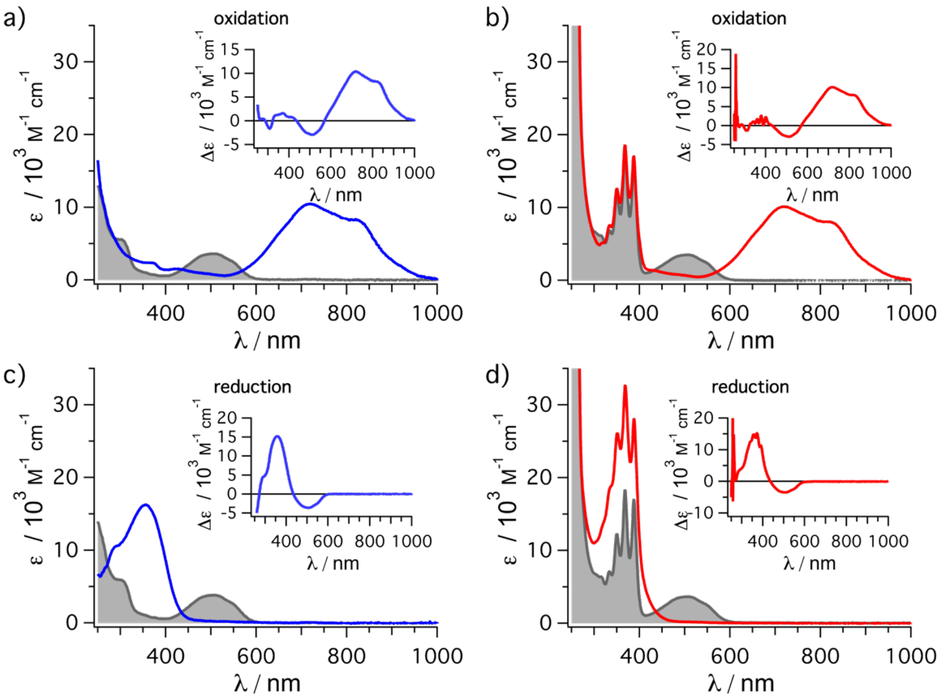

Figure S8. Spectroelectrochemistry for the oxidation (top row) and reduction (bottom row) of $[Fe(L^{Ph})_2]^+$ (blue) and $[Fe(L^{PhAn})_2]^+$ (red) in dichloromethane. The complexes without applied potential (grey) were used as internal reference to calculate the change in molar absorption coefficients. The measurements were performed in the presence of 100 mM tetrabutylammonium hexafluorophosphate and the (a+b) oxidized and (c+d) reduced complexes were obtained with a constant potential of +1.0 V *vs* Ag/AgCl and –1.1 V *vs* Ag/AgCl, respectively. The insets represent the difference spectra between the oxidized or reduced state and the ground state.

Summary of the Electrochemical and Spectroelectrochemical Properties

Table S5. Overview of properties obtained by electrochemical measurements in different solvents.

**Oxidation**	**Solvent**	$E_{ox}(PS^{n+/(n+1)+})$ **/ V *vs* SCE** [a]	λ_{abs} **(Δε) / nm (**10^3 M^{-1} s^{-1}**)** [b]
$[Fe(L^{Ph})_2]^+$	Acetonitrile [c]	+0.68	716 (9.0) [1]
	Acetone	+0.76	
	Butyronitrile	+0.78	
	Tetrahydrofuran	+0.85	
	Dichloromethane	+0.70	719 (10.4)
$[Fe(L^{PhAn})_2]^+$	Acetonitrile [c]	+0.70, +1.35	717 (10.8) [1]
	Acetone	+0.73, +1.32	
	Butyronitrile	+0.75, +1.18	
	Tetrahydrofuran	+0.80, +1.38	
	Dichloromethane	+0.68, +1.22	721 (10.1)
PhAn	Acetonitrile [c]	+1.33	
	Acetone	+1.35	
	Butyronitrile	+1.39	
	Tetrahydrofuran	+1.44	
	Dichloromethane	+1.29	358 (13.0), 584 (4.0), 646 (4.8), 720 (6.0) [1]
**Reduction**	**Solvent**	$E_{red}(PS^{n+/(n-1)+})$ **/ V *vs* SCE** [a]	λ_{abs} **(Δε) / nm (**10^3 M^{-1} s^{-1}**)** [b]
$[Fe(L^{Ph})_2]^+$	Acetonitrile [c]	–0.72	357 (12.3)
	Acetone	–0.66	
	Butyronitrile	–0.67	
	Tetrahydrofuran	–0.57	
	Dichloromethane	–0.84	357 (16.3)
$[Fe(L^{PhAn})_2]^+$	Acetonitrile [c]	–0.74, –2.00	358 (15.0) [1]
	Acetone	–0.69, –1.93	
	Butyronitrile	–0.69, –1.93	
	Tetrahydrofuran	–0.60, –1.90	
	Dichloromethane	–0.84	356 (14.9)
PhAn	Acetonitrile	–1.98	
	Acetone	–1.91	
	Butyronitrile	–1.85	
	Tetrahydrofuran	-	
	Dichloromethane	-	

Values estimated from the peak maximum in the DPV [a] Measured values are reported *vs* Ag/AgCl and converted *vs* SCE by subtracting 0.045 V based on the cyclic voltammograms in **Figure S4** to **Figure S7**. [b] Molar absorption coefficients of oxidized and reduced species are reported in acetonitrile, all molar absorption coefficients (ε) are calculated relative to the ground state molar absorption coefficients for the first oxidation or reduction. [c] These values were reported in another study.[1]

2.1.2. Molar Absorption Coefficient of ^{3*}PhAn in Different Solvents

The molar absorption coefficient of ^{3*}PhAn was determined with $[Ru(bpy)_3]^{2+}$ as actinometer ($\Delta\varepsilon_{3Ru,455nm} = -10100\ M^{-1}\ cm^{-1}$ at 455 nm).[13,14] The procedure has been described in more detail recently.[1] In summary, the molar absorption coefficient of ^{3*}PhAn ($\Delta\varepsilon_{3PhAn,430nm}$) can be obtained using equation 1.

$$\Delta\varepsilon_{3PhAn,430nm} = \frac{\Delta A_{3Ru,455nm,t=0}}{\Delta A_{3PhAn,430nm}} \cdot \Delta\varepsilon_{3Ru,455nm} \cdot \frac{1}{\Phi_{TTET}} \qquad \text{Eq. 1}$$

The determined molar absorption coefficient of ^{3*}PhAn ($\Delta\varepsilon_{3PhAn,430nm}$) at 430 nm in dichloromethane is $21300 \pm 900\ M^{-1}\ cm^{-1}$ and the full spectrum is obtained by rescaling the transient absorption spectrum accordingly (Figure S9).

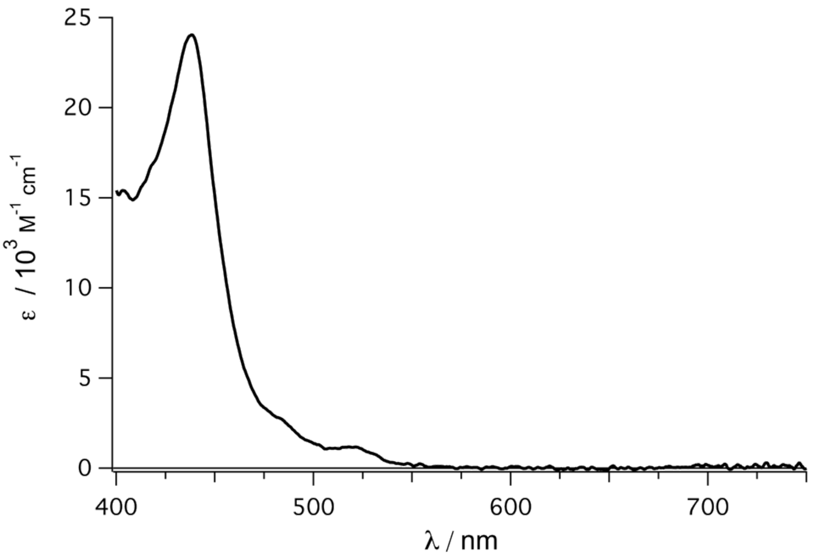

Figure S9. Calibrated transient absorption spectra of ^{3*}PhAn in dichloromethane based on the molar absorption coefficient determined at 430 nm (see Table S6) and the transient absorption spectrum obtained by sensitized formation with $[Ru(bpy)_3]^{2+}$.

Table S6. Overview of the relevant measured values determined by transient absorption spectroscopy with different laser intensities for excitation and the calculated molar absorption coefficient of ^{3*}PhAn.

average laser intensity per pulse / mJ	$\Delta A_{3Ru,455nm,t=0}$	$\Delta A_{3PhAn,430nm}$	Φ_{TTET} / %	$\Delta\varepsilon_{3PhAn,430nm}$ / $M^{-1}cm^{-1}$ [a]
2	–0.0108	0.0223	87	22486
4	–0.0165	0.0337	87	22332
6	–0.0222	0.0440	87	21568
8	–0.0271	0.0523	87	20993
10	–0.0298	0.0572	87	20910
12	–0.0333	0.0622	87	20382
15	–0.0367	0.0678	87	20143
			Average:	21300 ± 900

[a] The molar absorption coefficient of ^{3*}PhAn is calculated for each line according to equation 1.

Repeating the same measurements in different solvents enabled the determination of ^{3*}PhAn in tetrahydrofuran, butyronitrile and acetone (see Figure S10 and Table S7). It is worth mentioning that in THF, $[Ir(ppy)_3]$ was used as sensitizer for the formation of ^{3*}PhAn due to the limited solubility of $[Ru(bpy)_3]^{2+}$.

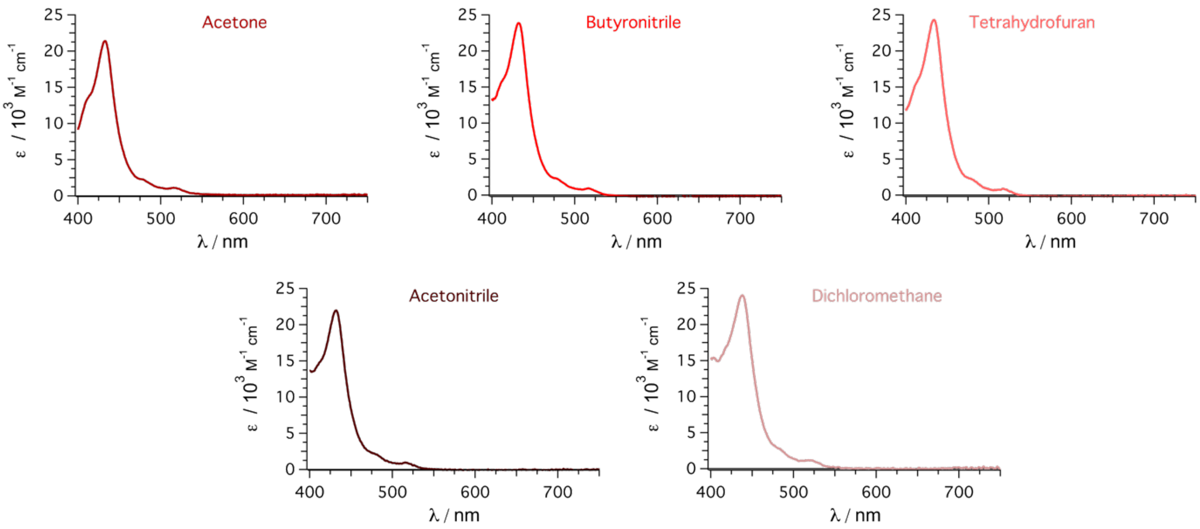

Figure S10. Calibrated transient absorption spectra of ^{3*}PhAn in different solvents based on the molar absorption coefficient determined at 430 nm. The molar absorption coefficients at the peak maximum are reported in Table S7.

Table S7. Overview of the molar absorption coefficient of ^{3*}PhAn in different solvents.

solvent	$\lambda_{3PhAn,max}$	$\Delta\varepsilon_{3PhAn,\lambda max}$ / $M^{-1}cm^{-1}$
Acetonitrile	432	21900 ± 500 [1]
Acetone	432	21400 ± 500
Tetrahydrofuran	434	24300 ± 500
Butyronitrile	432	23900 ± 600
Dichloromethane	438	24000 ± 1000

2.1.3. Intersystem Crossing Quantum Yield of ^{1*}PhAn in Different Solvents

The intersystem corssing quantum yield of substitued anthracenes can vary significanly depending on the substitution.[8,15] To elucidate if a direct intersystem crossing (ISC) from ^{1*}PhAn to ^{3*}PhAn could potentially play a role upon excitation around 360 nm, where the absorption of the dyad is predominately caused by the anthracene-based absorption, intersystem crossing quantum yields $\Phi_{ISC,PhAn}$ were evaluated. In the literature, different values between ~0.2 and ~0.5 were reported depending on the respective solvent, specifically ~0.5 (ethanol)[16,17] and 0.35-0.37 (liquid paraffin)[16,18,19] and 0.24 (ethylene glycol)[18].

Intersystem-crossing quantum yields of PhAn in different solvents were determined by relative actinometry against the bleach of excited $[Ru(bpy)_3]^{2+}$ ($\Delta\varepsilon_{3Ru,455nm}$ = –10100 M^{-1} cm^{-1} at 455 nm)[13,14] in acetonitrile for isoabsorptive solutions at the excitation wavelength of 355 nm. The results are summarised in Table S8 and indicate indeed potentially non-neglitable $\Phi_{ISC,PhAn}$.

Table S8. Overview of the intersystem crossing quantum yields of ^{3*}PhAn and ^{1*}PhAn lifetime in different solvents.

**solvent**	$\Phi_{ISC,PhAn}$ [a]	τ_{1*PhAn} / **ns** [b]
Acetonitrile	0.35	5.3
Acetone	0.40	- [c]
Butyronitrile	0.35	- [c]
Tetrahydrofuran	0.25	- [c]
Dichloromethane	0.30	4.8

[a] Intersystem crossing quantum yields determined by relative actinometry with $[Ru(bpy)_3]^{2+}$ in acetonitrile as actinometer ($\Delta\square_{3Ru,455nm}$ = –10100 M^{-1} cm^{-1} at 455 nm).[13,14] Molar absorption coefficients of PhAn in different solvents are described in section 2.1.2. Values rounded to 0.05. [b] Lifetime of ^{1*}PhAn measured by fs-TAS, data provided in section 3.2.5. [c] Not measured within this study.

Gratefully, due to the ultrafast oxidative quenching of ^{1*}PhAn, resulting in a lifetime of around 5 ps ($k_r \approx 2\cdot10^{11}$ s^{-1}) within the $[Fe(L^{PhAn})_2]^+$ dyad in acetonitrile and dichloromethane, the process is several orders of magnitudes faster than an estimated rate constant for the intersystem crossing ($k_{ISC} = k_r\cdot\Phi_{ISC}/(1-\Phi_{ISC})$) on the anthracene moiety. The rate constant of intersystem crossing in PhAn is estimated as $k_{ISC} \sim 10^7$-10^8 s^{-1} from a simplified model with the assumption of radiative deactivation and intersystem crossing as the only deactivation pathways. Hence, assuming similar intersystem-crossing rate constants in free PhAn and PhAn within the dyad, the intramolecular electron transfer is about three orders of magnitude faster than the intersystem crossing, and as a consequence the contribution of ISC on our analysis of the dyad is negligible.

3. Transient-Absorption Spectroscopy

3.1. Spin States and Overview of Possible Mechanistic Scenarios

In addition to the comparison of different intersystem crossing pathways starting from a dyad in a singlet or doublet state, a comparison of the mechanistic pathway and spin states of the involved states by electron transfer intersystem crossing upon excitation of the iron complex (**Figure S11a**), the PhAn (**Figure S11b**) or a possible doublet-triplet energy transfer (**Figure S11c**) are presented here.

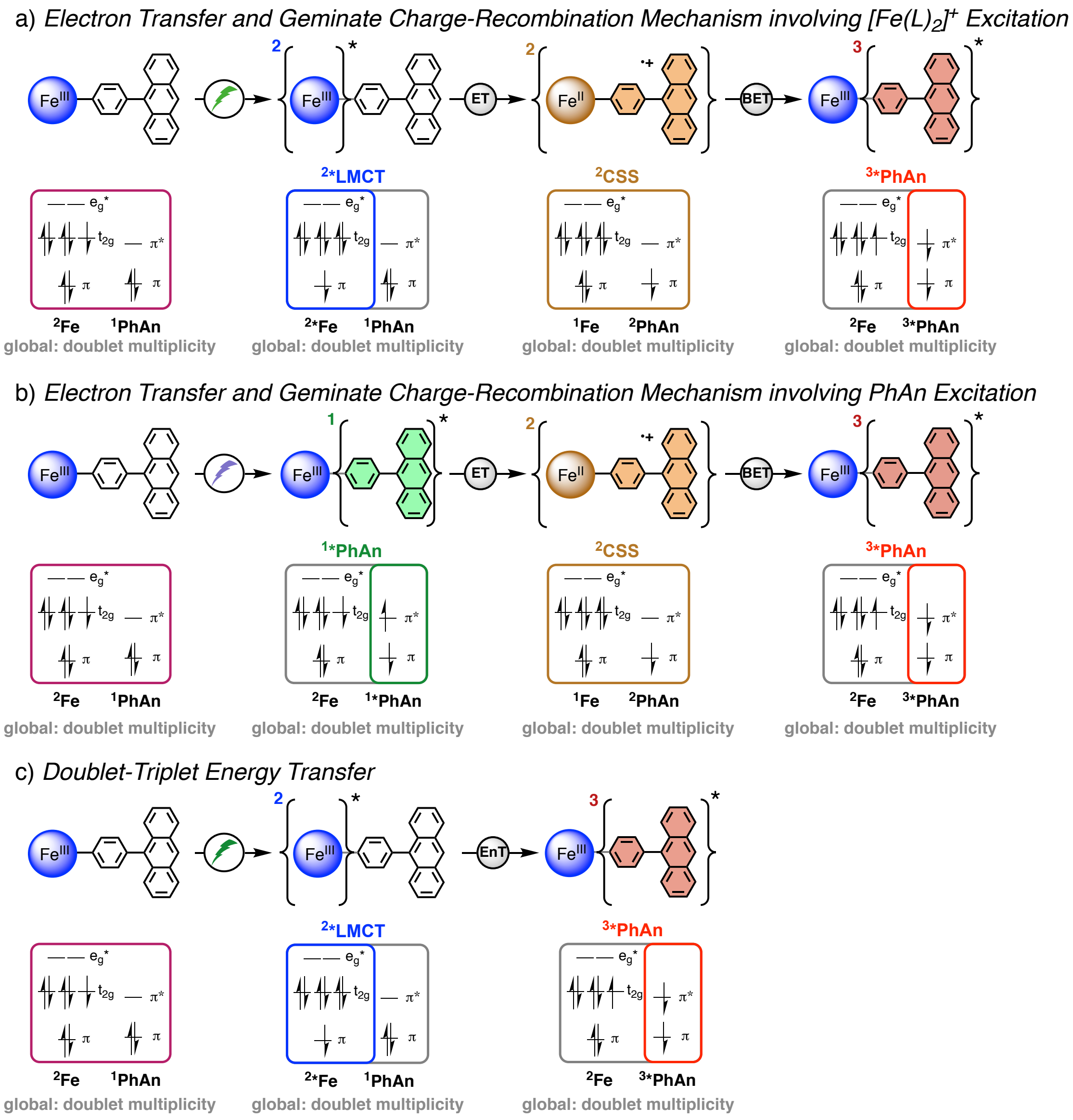

Figure S11. Elementary steps and spin states to populate a (local) triplet excited states by different pathways: a) *via* excitation of the iron complex; or b) *via* excitation of the PhAn moiety of the dyad followed by several electron transfer steps; or c) a doublet-triplet energy transfer pathway.

3.2. Femtosecond Transient-Absorption Spectroscopy

3.2.1. Spectral Assignment of Species-Associated Differential Spectra

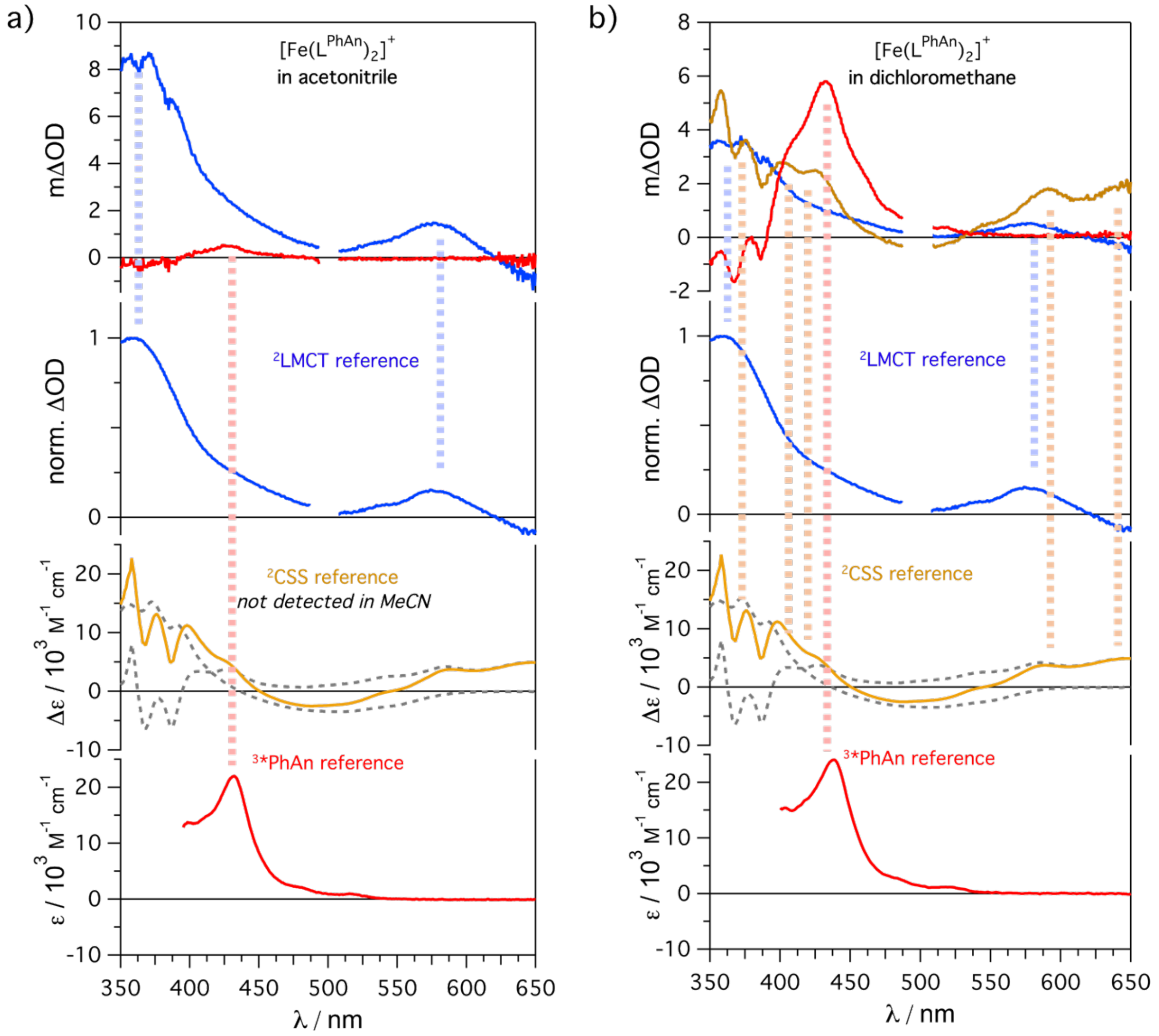

Figure S12. Assignment of the species-associated differential spectra (SADS, top panel) in (a) acetonitrile and (b) dichloromethane. Reference spectra for ^{2}LMCT (second panel, fs-TAS, Figure S24), ^{2}CSS (third panel, electrochemistry, section 2.1.1) and ^{3}*PhAn (fourth panel, ns-TAS, section 2.1.2) gathered trough complementary methods. Small differences between the simulated ^{2}CSS, consiting of a linear combination of the electrochemically generated spectra of $[Fe^{II}(L^{Ph})_2]$ and $PhAn^{\bullet+}$ (dotted lines in third panel), are attributed to imprecise values of the molar absorption coefficients due to the limited stability of the oxidized 9-phenylanthracene.[1]

3.2.2. Fit Model for Target Analysis of fs-Transient Absorption Datasets

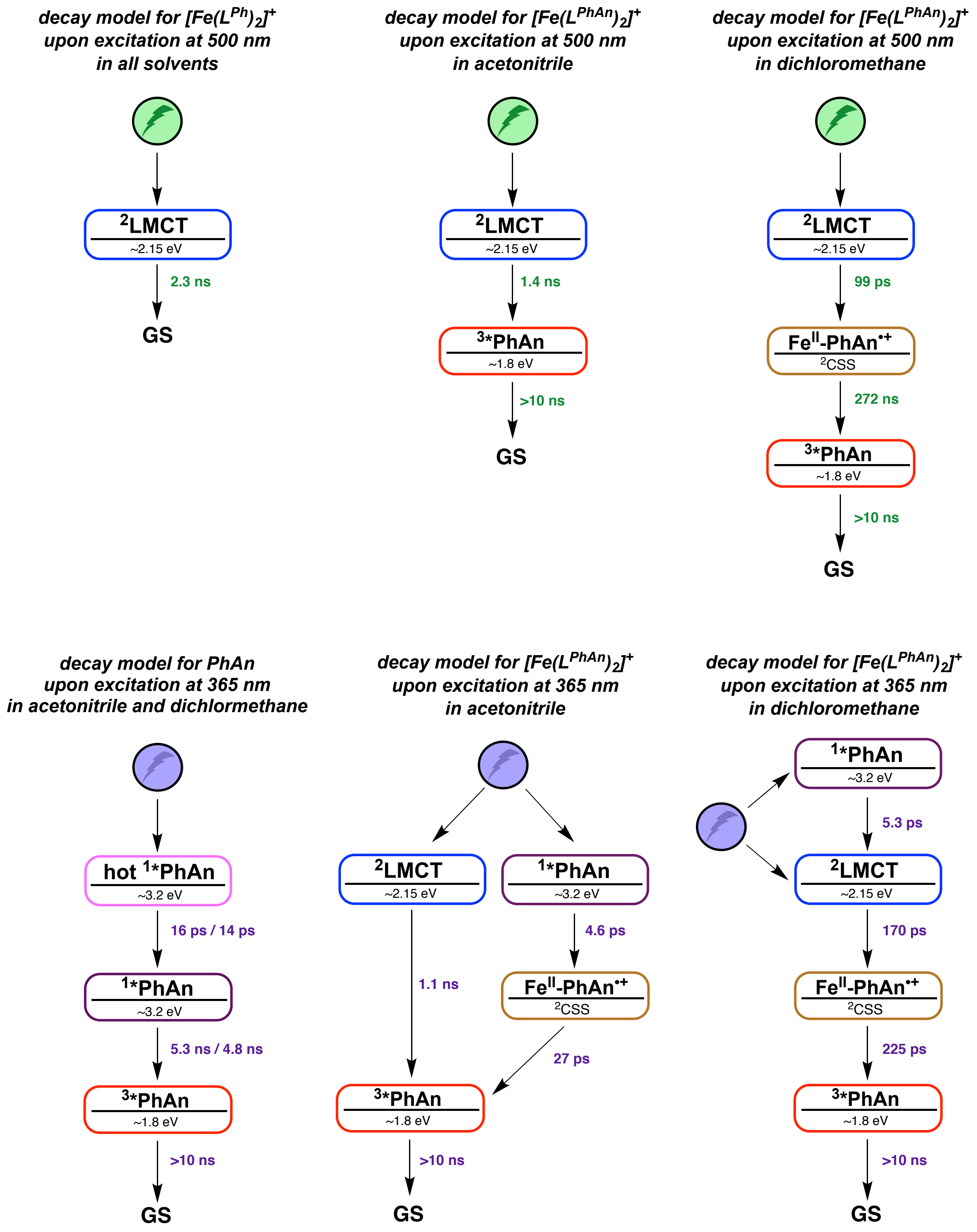

Figure S13. Different models employed for target analysis of fs-TAS measurements.

The models for target analysis to fit the fs-TAS are shown in Figure S13 and the results are provided in figures of the main manuscript and together with the differential absorption 3D maps in section 3.2.3 to section 3.4.2.

3.2.3. fs-TA-Spectroscopy of $[Fe(L^{PhAn})_2]^+$ Excited at 500 nm

fs-TA-Spectroscopy of $[Fe(L^{PhAn})_2]^+$ in Acetonitrile

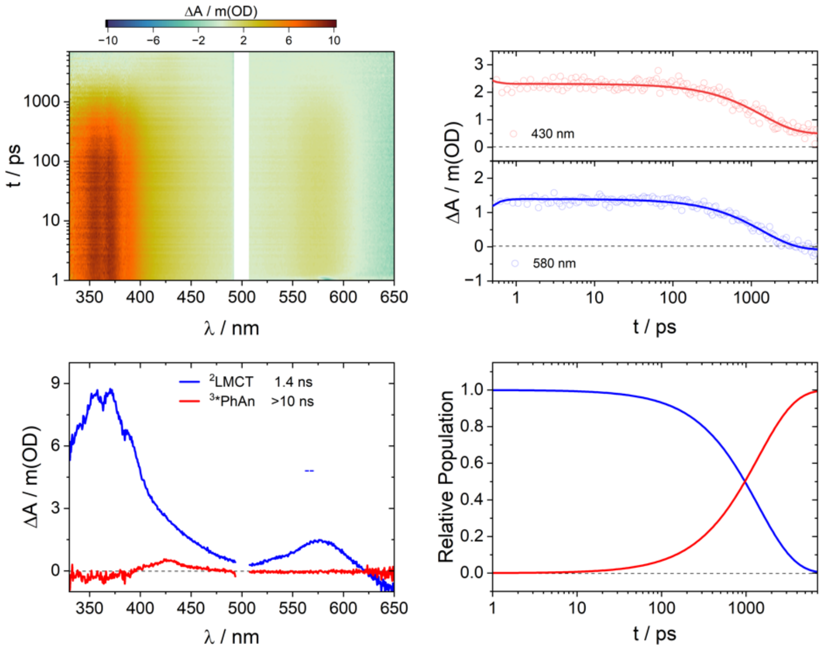

Figure S14. Top left: Differential absorption 3D map obtained from fsTAS for $[Fe(L^{PhAn})_2]^+$ in acetonitrile upon excitation at 500 nm. Top right: time absorption profiles (open circles) and corresponding fittings from target analysis (solid lines) using a model described in **Figure S13**. Bottom left: species-associated differential spectra obtained by target analysis. Bottom right: relative concentration evolution over time.

fs-TA-Spectroscopy of $[Fe(L^{PhAn})_2]^+$ in Dichloromethane

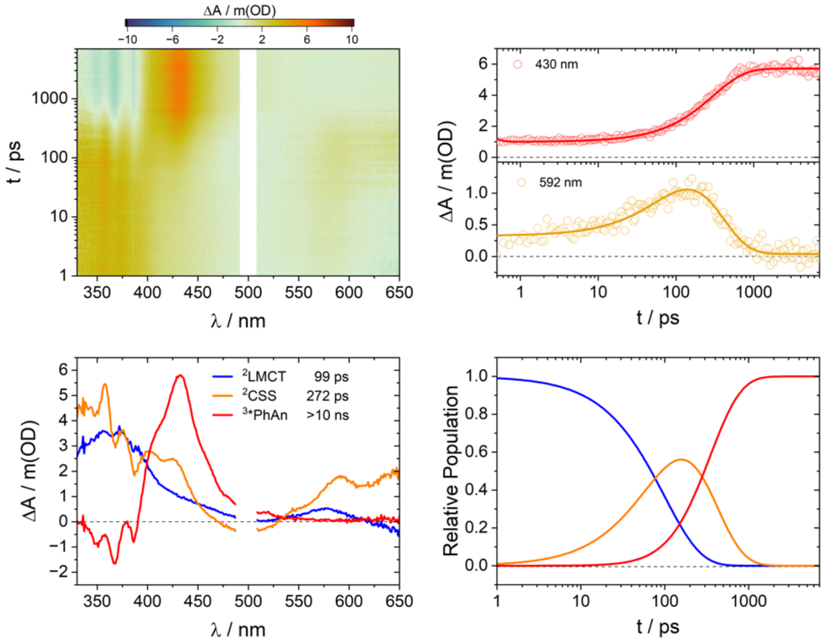

Figure S15. Top left: Differential absorption 3D map obtained from fsTAS for $[Fe(L^{PhAn})_2]^+$ in dichloromethane upon excitation at 500 nm. Top right: time absorption profiles (open circles) and corresponding fittings from target analysis (solid lines) using a model described in **Figure S13**. Bottom left: species-associated differential spectra obtained by target analysis. Bottom right: relative concentration evolution over time.

3.2.4. fs-TA-Spectroscopy of [Fe(L^{PhAn})$_2$]$^+$ Excited at 365 nm

fs-TA-Spectroscopy of [Fe(L^{PhAn})$_2$]$^+$ in Acetonitrile

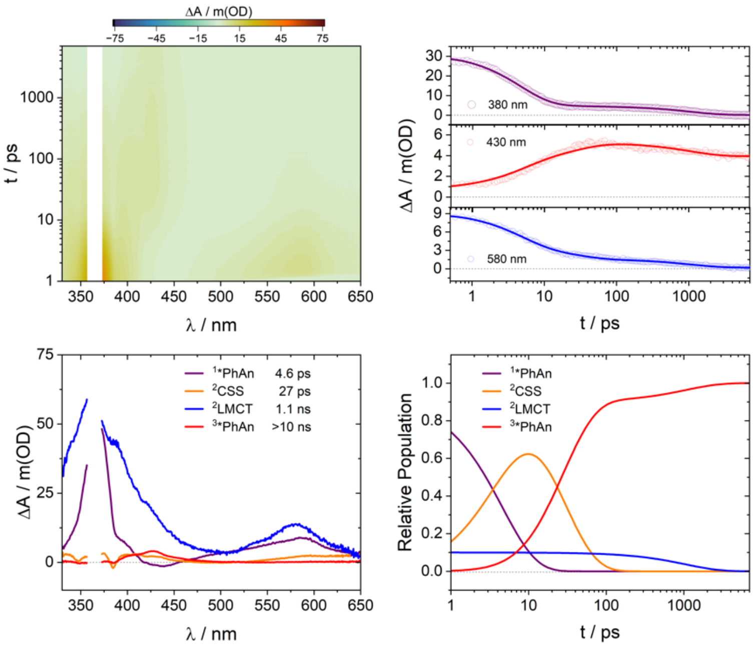

Figure S16. Top left: Differential absorption 3D map obtained from fsTAS for [Fe(L^{PhAn})$_2$]$^+$ in acetonitrile upon excitation at 365 nm. Top right: time absorption profiles (open circles) and corresponding fittings from target analysis (solid lines) using a model described in **Figure S13**. Bottom left: species-associated differential spectra obtained by target analysis. Bottom right: relative concentration evolution over time.

fs-TA-Spectroscopy of [Fe(L^{PhAn})$_2$]$^+$ in Dichloromethane

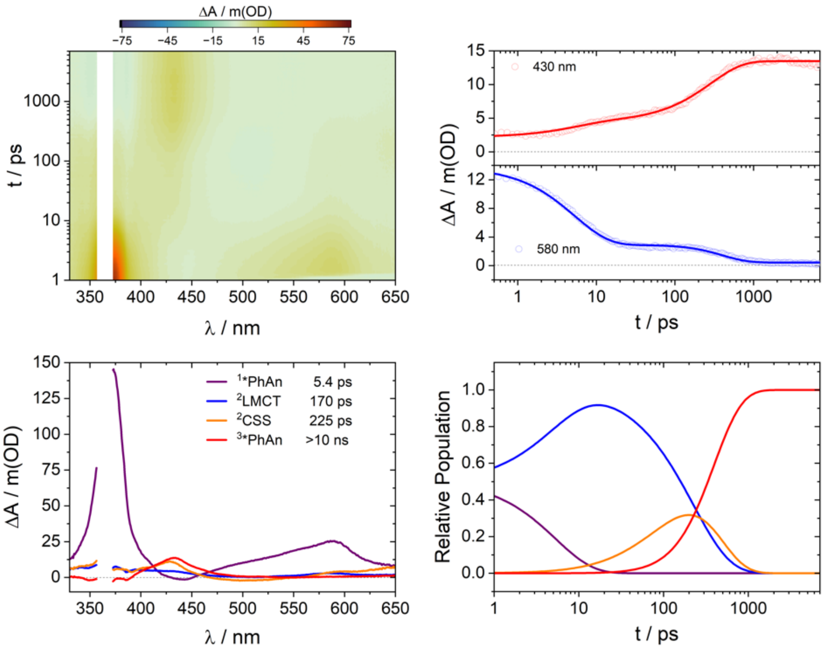

Figure S17. Top left: Differential absorption 3D map obtained from fsTAS for [Fe(L^{PhAn})$_2$]$^+$ in dichloromethane upon excitation at 365 nm. Top right: time absorption profiles (open circles) and corresponding fittings from target analysis (solid lines) using a model described in **Figure S13**. Bottom left: species-associated differential spectra obtained by target analysis. Bottom right: relative concentration evolution over time.

3.2.5. fs-TA-Spectroscopy of PhAn Excited at 365 nm

fs-TA-Spectroscopy of PhAn in Acetonitrile

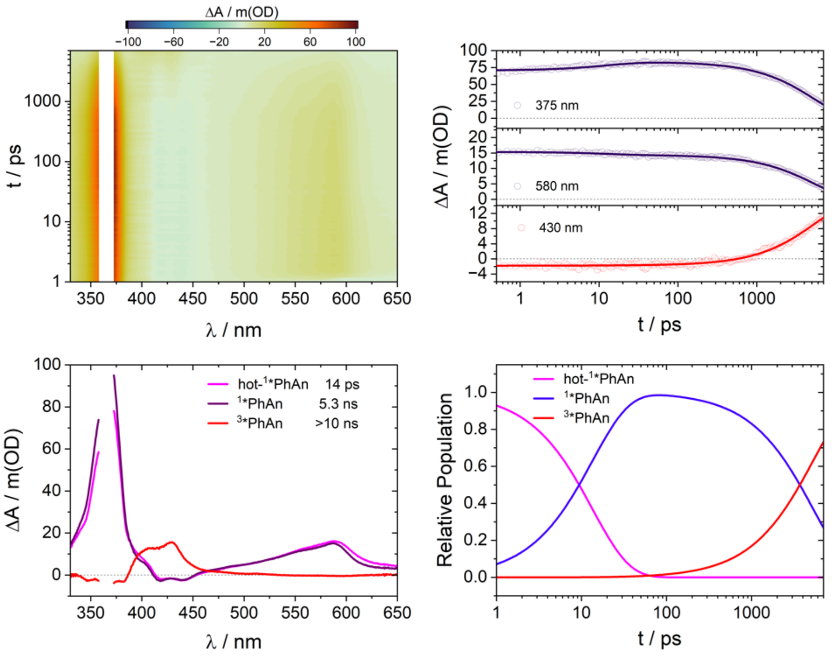

Figure S18. Top left: Differential absorption 3D map obtained from fsTAS for PhAn in acetonitrile upon excitation at 365 nm. Top right: time absorption profiles (open circles) and corresponding fittings from target analysis (solid lines) using a model described in **Figure S13**. Bottom left: species-associated differential spectra obtained by target analysis. Bottom right: relative concentration evolution over time.

fs-TA-Spectroscopy of PhAn in Dichloromethane

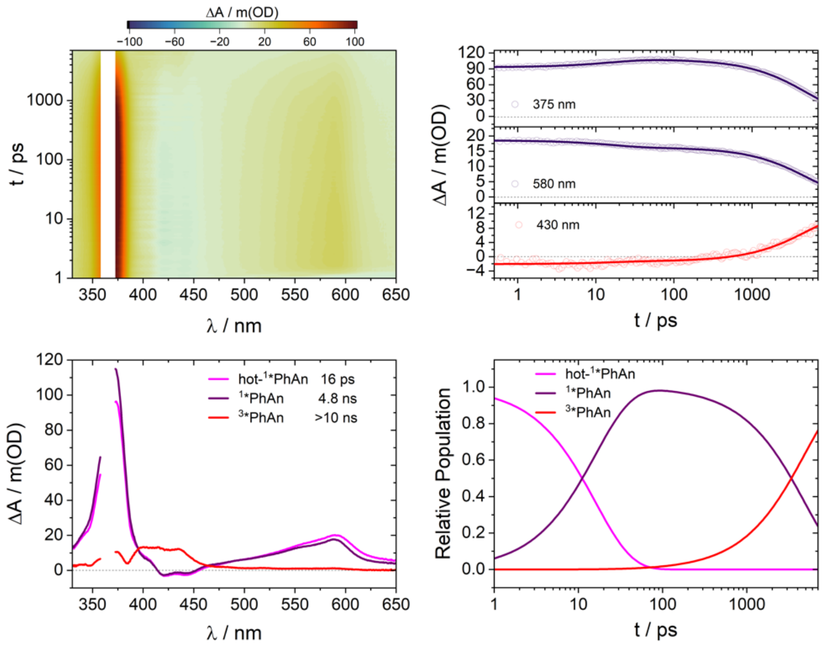

Figure S19. Top left: Differential absorption 3D map obtained from fsTAS for PhAn in dichloromethane upon excitation at 365 nm. Top right: time absorption profiles (open circles) and corresponding fittings from target analysis (solid lines) using a model described in **Figure S13**. Bottom left: species-associated differential spectra obtained by target analysis. Bottom right: relative concentration evolution over time.

3.3. Bimolecular Quenching of $[Fe(L^{Ph})_2]^+$ by PhAn

We were also interested in investigating if bimolecular quenching of the unsubstituted photosensitizer by 9-phenylanthracene could also potentially involve a CSS. Recently, Wang and coworkers reported a DTET between $[Fe(L^{Ph})_2]^+$ and different anthracenes in DMSO to establish a sensitized triplet-triplet annihilation upconversion system.[20] Bimolecular quenching constants of 10^{10}-10^{11} $M^{-1}s^{-1}$ exceeding the diffusion limit were reported based on steady-state emission-based Stern-Volmer analysis, and interpreted in terms of pre-association according to NMR titration experiments which indicated association constants of around 10^2 M^{-1}. However, in their femtosecond transient absorption data, a charge-separated state was not observed. We hypothesized that the limited solubility of the polyaromatic hydrocarbon in polar solvent would complicate the detection of a potentially short-lived intermediate.

Hence, fs-TA measurements upon 500 nm excitation, with 58 mM of PhAn in DCM were used to investigate the pathways for bimolecular quenching (Figures S21 and S22). Notably, inspection of the trace at 430 nm revealed at least two exponential processes in the sub-ns timescale, which suggested that also here a ^{2}CSS participated in the decay cascade. An all-sequential model failed to afford physically meaningful differential spectra. Therefore, the target model depicted in Figure S22 was employed to fit the data. In our model, 20 % of the initially excited ^{2}LMCT underwent an ultrafast charge separation forming an intermolecular ^{2}CSS $\{[Fe^{II}(L^{Ph})_2];PhAn^{\bullet+}\}$ in less than 1 ps, *via* static quenching within pre-associated pairs, in agreement with previous findings.[20] In this context, our observations started with 80% of the excited-state population in ^{2}LMCT, and 20% in an intermolecular ^{2}CSS. Subsequently, the remaining ^{2}LMCT population feeds ^{3*}PhAn in 830 ps, while 80% of ^{2}CSS recombines into ^{3*}PhAn and the remaining 20% of ^{2}CSS recombines to the ground state in 19 ps. Variations on the branching fractions were found to afford worse differential spectra without physical meaning. Nevertheless, since branching ratios were fixed during the fitting process, their exact values should be taken with care. Interestingly, target analysis of the dataset revealed a direct DTET as main pathway responsible for an 83% of the total triplet-state population yield, while at least a contribution of 17 % of the total triplet yield proceeds *via* the formation of ^{2}CSS due to static quenching (SI section 3.2.2). It is noteworthy that the ^{2}LMCT lifetime is much longer than the intermolecular ^{2}CSS lifetime. Therefore, it is likely that ^{2}CSS formed upon diffusional quenching also mediates, at least partially, the DTET in the bimolecular system, but a detection of the short-lived intermediate is justifiably not possible. With 8.8 mM of PhAn in MeCN, a similar behavior was observed (Figures S20 and S22). Support for our interpretation came from

Stern-Volmer fs-TA experiments, which afforded a linear dependence and essentially identical quenching constants of 2.2 and 1.4 x 10^{10} $M^{-1}s^{-1}$, in MeCN and DCM, respectively. Additionally, in both cases the lifetime of the intermolecular ^{2}CSS remain unchanged (Figure S22). The fact that only 20 % of the excited-state population visits an intermolecular ^{2}CSS state might have contributed to its elusiveness in previous studies, but our data clearly unambiguously demonstrates its participation in the bimolecular quenching process and this correlates with the behavior of the dyad.

In MeCN, the intermolecular ^{2}CSS has a similar lifetime than the intramolecular ^{2}CSS in the dyad, with 74 ps and 27 ps respectively. In DCM, however, the intermolecular ^{2}CSS is one order of magnitude shorter-lived than the intramolecular ^{2}CSS in the dyad, with 19 ps and over 200 ps respectively. This suggests that, since electron and energy transfer are both distance dependent, differences between the relative orientation of the redox species within the encounter complex formed *via* diffusion of freely moving molecules and the one from the dyad with a fix geometry and distance could play a major role. Thus, it appears that the results obtained with the dyad represent a delicate balance between the energy levels of the different states as well as the geometric arrangement between the photosensitizer and the final energy acceptor, as indicated by the dependence on the radii of the electron donor and acceptor moieties in Eq. 1 of the main manuscript. At this stage, we are currently unable to ascertain if the diffusional excited-state reaction is occurring via DTET or mediated by the CSS, as small changes within redox potentials, for instance due to a change of the Coulomb interaction between charges to influence the energy of the CSS,[21] or changes is geometry might be paramount.

3.3.1. fs-TA-Spectroscopy of $[Fe(L^{Ph})_2]^+$ in the Presence of PhAn Excited at 500 nm

fs-TA-Spectroscopy of $[Fe(L^{Ph})_2]^+$ in the Presence of PhAn in Acetonitrile

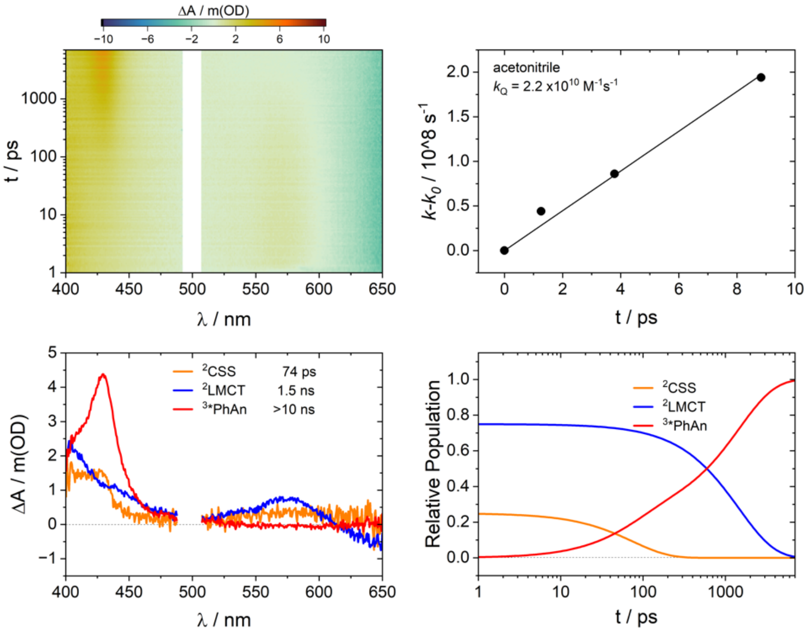

Figure S20. Top left: Differential absorption 3D map obtained from fsTAS for $[Fe(L^{Ph})_2]^+$ in the presence of 8.8 mM PhAn in acetonitrile upon excitation at 500 nm. Top right: Stern-Volmer plot based on the ^{2}LMCT lifetime with different concentrations of PhAn. Bottom left: species-associated differential spectra obtained by target analysis in the presence of 8.8 mM PhAn in acetonitrile. Bottom right: relative concentration evolution over time in the presence of 8.8 mM PhAn in acetonitrile.

fs-TA-Spectroscopy of $[Fe(L^{Ph})_2]^+$ in the Presence of PhAn in Dichloromethane

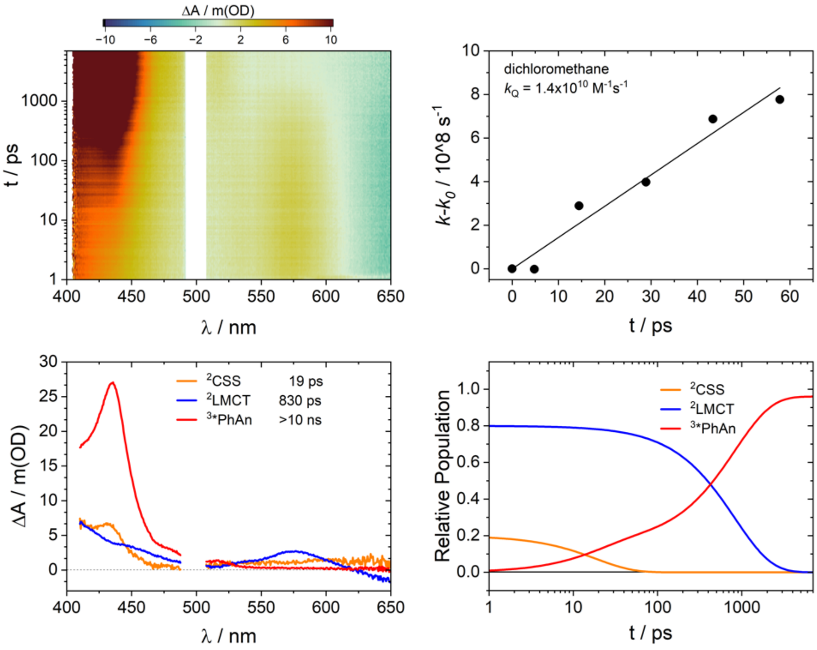

Figure S21. Top left: Differential absorption 3D map obtained from fsTAS for $[Fe(L^{Ph})_2]^+$ in the presence of 58 mM PhAn in dichloromethane upon excitation at 500 nm. Top right: Stern-Volmer plot based on the ^{2}LMCT lifetime with different concentrations of PhAn. Bottom left: species-associated differential spectra

obtained by target analysis in the presence of 58 mM PhAn in dichloromethane. Bottom right: relative concentration evolution over time in the presence of 58 mM PhAn in dichloromethane.

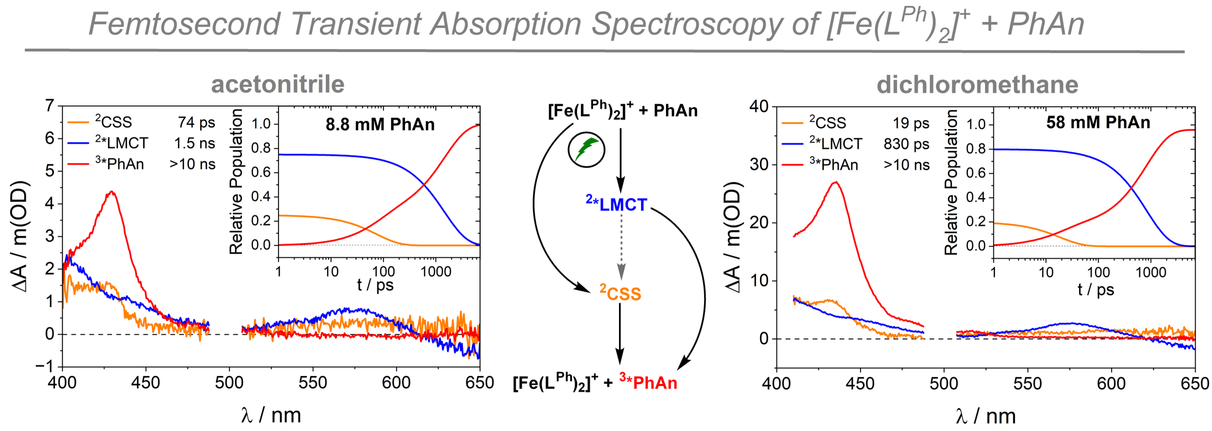

Figure S22. Species-associated differential spectra obtained by target analysis of $[Fe(L^{Ph})_2]^+$ in the presence of 8.8 mM and 58 mM of PhAn in MeCN (left) and DCM (right) respectively upon excitation at 500 nm. The corresponding relative populations of the individual excited states are shown as inset of the respective figure section. The color-code for the individual excited states is presented as inset and matches the scheme in the middle. Electronic configurations of the individual steps are shown in detail in the SI (section 3.1). Further spectroscopic details, figures and models for global fitting are presented in the Supporting Information (SI section 3.1).

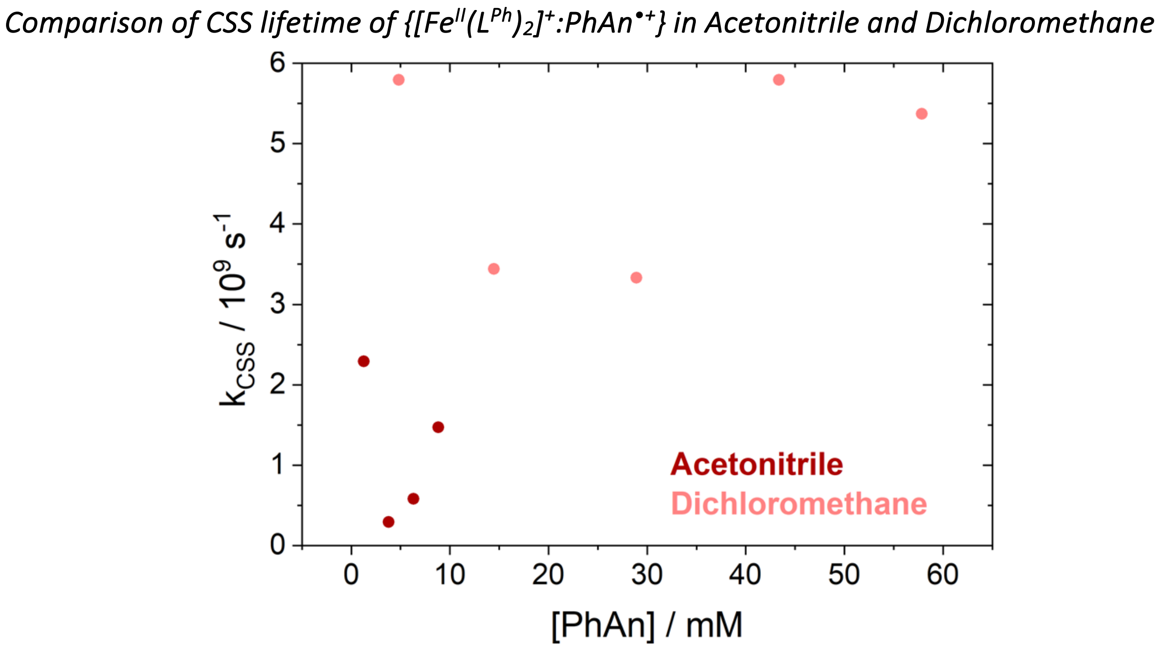

Figure S23. Decay constant for ^{2}CSS derived from target analysis of the fsTAS data for $[Fe(L^{Ph})_2]^+$ in the presence of different concentrations of PhAn in acetonitrile and dichloromethane. Exemplified datasets are presented in Figure S20 and Figure S21.

3.3.2. fs-TA-Spectroscopy of [Fe(L^{Ph})$_2$]$^+$ Excited at 500 nm

fs-TA-Spectroscopy of [Fe(L^{Ph})$_2$]$^+$ in Dichloromethane

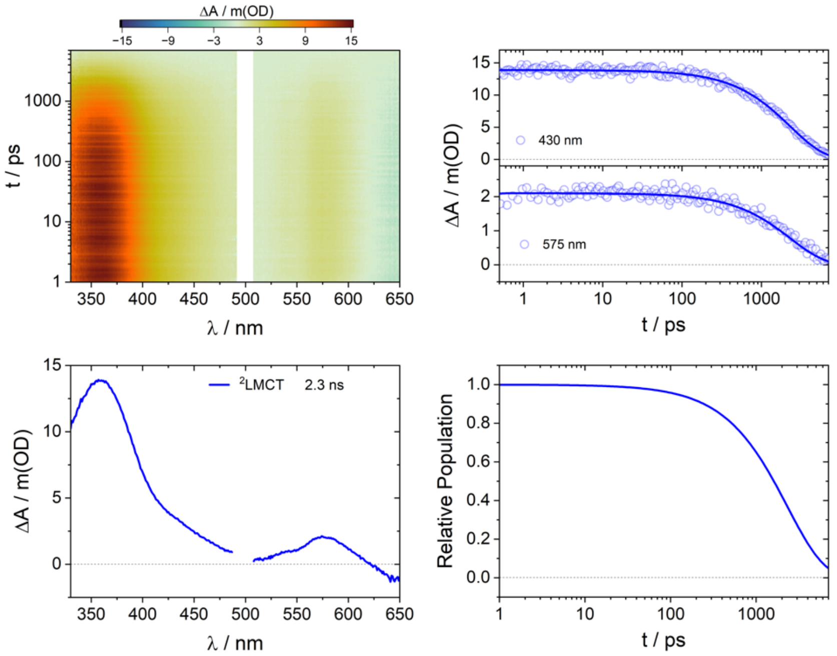

Figure S24. Top left: Differential absorption 3D map obtained from fsTAS for [Fe(L^{Ph})$_2$]$^+$ in dichloromethane upon excitation at 500 nm. Top right: time absorption profiles (open circles) and corresponding fittings from target analysis (solid lines) using as described in **Figure S13**. Bottom left: species-associated differential spectra obtained by global analysis. Bottom right: relative concentration evolution over time.

3.4. Nanosecond Transient-Absorption Spectroscopy

3.4.1. ns-TA-Spectroscopy: $^{3*}[Fe(L^{PhAn})_2]^+$ Lifetimes

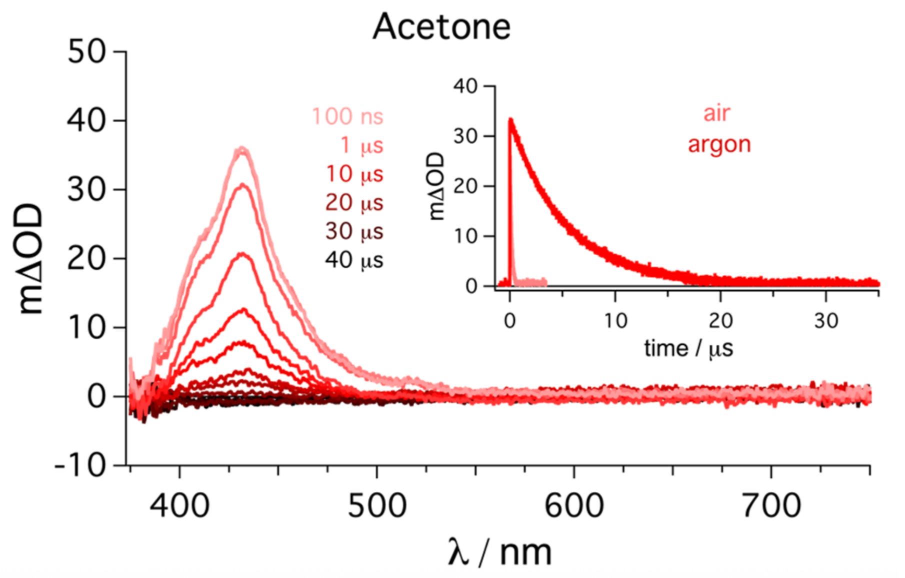

Figure S25. Time-resolved transient absorption spectroscopy of $[Fe(L^{PhAn})_2]^+$ (100 µM) in de-aerated acetone measured with different time delays after the laser excitation at 500 nm, time-integrated over 200 ns. The inset presents the kinetic decay of $^{3*}[Fe(L^{PhAn})_2]^+$ monitored at 430 nm under aerated and deaerated conditions. A 375 nm longpass filter (Newport) was installed for all measurements between the flashlamp and the quartz cuvette.

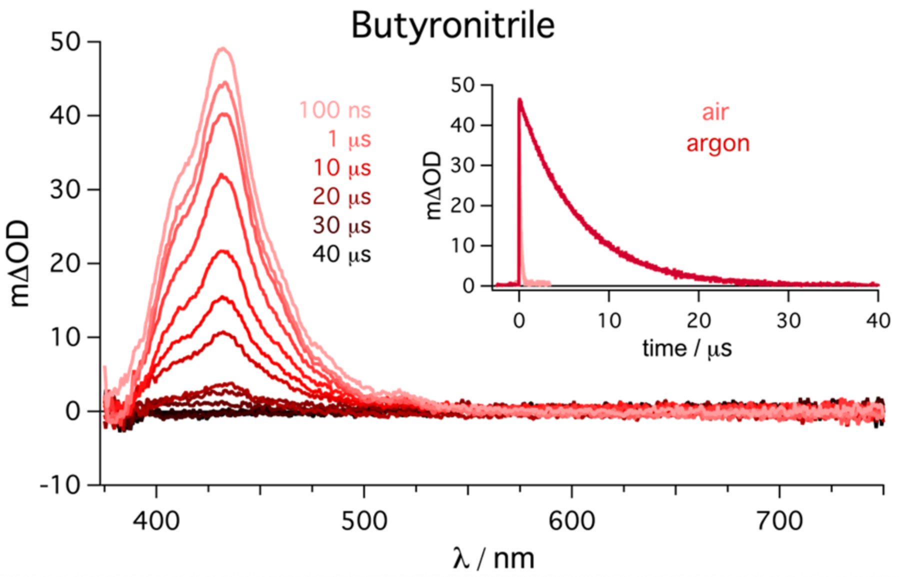

Figure S26. Time-resolved transient absorption spectroscopy of $[Fe(L^{PhAn})_2]^+$ (100 µM) in de-aerated butyronitrile measured with different time delays after the laser excitation at 500 nm, time-integrated over 200 ns. The inset presents the kinetic decay of $^{3*}[Fe(L^{PhAn})_2]^+$ monitored at 430 nm under aerated and deaerated conditions. A 375 nm longpass filter (Newport) was installed for all measurements between the flashlamp and the quartz cuvette.

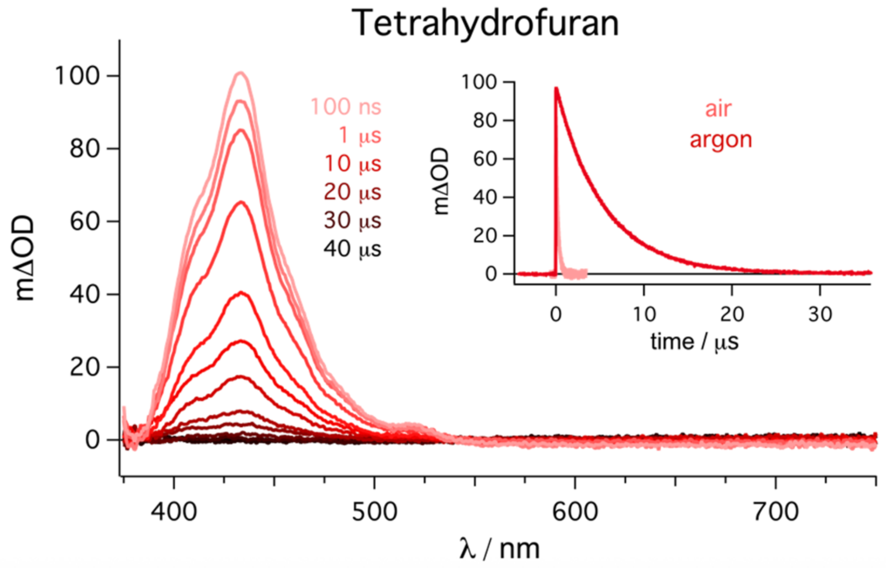

Figure S27. Time-resolved transient absorption spectroscopy of $[Fe(L^{PhAn})_2]^+$ (100 μM) in de-aerated tetrahydrofuran measured with different time delays after the laser excitation at 500 nm, time-integrated over 200 ns. The inset presents the kinetic decay of $^{3*}[Fe(L^{PhAn})_2]^+$ monitored at 430 nm under aerated and deaerated conditions. A 375 nm longpass filter (Newport) was installed for all measurements between the flashlamp and the quartz cuvette.

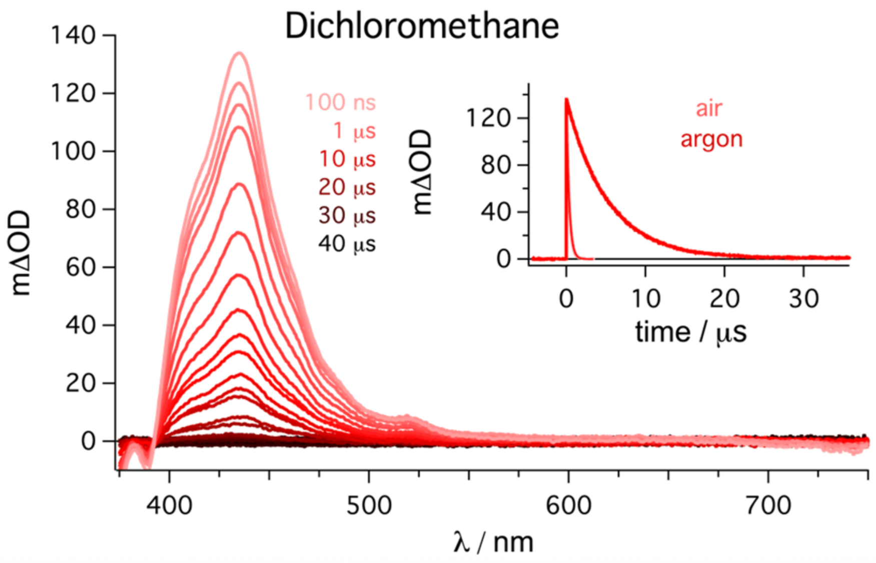

Figure S28. Time-resolved transient absorption spectroscopy of $[Fe(L^{PhAn})_2]^+$ (100 μM) in de-aerated dichloromethane measured with different time delays after the laser excitation at 500 nm, time-integrated over 200 ns. The inset presents the kinetic decay of $^{3*}[Fe(L^{PhAn})_2]^+$ monitored at 430 nm under aerated and deaerated conditions.

Table S9. Overview of the triplet excited-state lifetimes τ and population yields Φ of $^{3*}[Fe(L^{PhAn})_2]^+$ in different solvents.

solvent	$\tau_{3*[FePhAn],air}$ / ns	$\tau_{3*[FePhAn],Ar}$ / ns	k_{O2} / 10^9 $M^{-1}s^{-1}$	Φ ($^{3*}[Fe(L^{PhAn})_2]^+$)
Acetonitrile	165 [a]	11800 [a]	~3 [a]	0.05±0.01
Acetone	0.40	5400	~3	0.20
Butyronitrile	0.35	6600	~3	0.25
Tetrahydrofuran	0.25	5300	~2	0.50
Dichloromethane	0.30	5100	~1	0.75±0.02

Triplet state lifetimes are based on the kinetic traces in Figure S25 to Figure S28. Oxygen quenching rate constants were estimated based on an oxygen concentration of 1.9 mM (acetonitrile), 2.4 mM (acetone), 2.1 mM (tetrahydrofuran) and 2.2 mM (dichloromethane) under aerated conditions.[15] For butyronitrile a concentration of ~2 mM is assumed. [a] These values were reported in another study.[1]

3.4.2. ns-TA-Spectroscopy: $^{3*}[Fe(L^{PhAn})_2]^+$ Formation Quantum Yields

The triplet yield $\Phi_{3*FePhAn}$ of the dyad $^{3*}[Fe(L^{PhAn})_2]^+$ was determined by relative actinometry in different solvents against the bleach of a solution of $[Ru(bpy)_3]^{2+}$ at 455 nm ($\Delta\varepsilon = 10100\ M^{-1}cm^{-1}$)[13,14] on the nanosecond TA setup using equation 2.

$$\Phi_{3^*\text{FePhAn}} = \left(\frac{\frac{\Delta A_{3^*\text{PhAn},430nm}}{\Delta\varepsilon_{3^*\text{PhAn},430nm}}}{\frac{\Delta A_{ES_{ref}}}{\Delta\varepsilon_{ES_{ref}}}} \right) \left(\frac{1 - 10^{-Abs_{ref}(\lambda_{exc})}}{1 - 10^{-Abs_{[Fe(L^{PhAn})_2]}(\lambda_{exc})}} \right) \Phi_{\text{ref}} \qquad \text{Eq. 2}$$

The triplet formation yield of $[Ru(bpy)_3]^{2+}$ Φ_{ref} is set to unity and the relative concentration of $^{3*}[Fe(L^{PhAn})_2]^+$ and $[Ru(bpy)_3]^{2+}$ is determined and corrected for the differences in absorbance *Abs* at the excitation wavelength λ_{ex} of both solutions. The exctinction coefficient of the dyad is estimated based on the extincion coefficient of ^{3*}PhAn (see section 2.1.2 and Table S7).

In acetonitrile and dichloromethane, the two main solvents of this study, the triplet formation quantum yields were measured with different excitation laser power while in acetone, butyronitrile, and tetrahydrofuran the values were measured in a single point determination to obtain an estimation of the triplet formation yield. The determined values are summarised in **Table S8** and a comparison between excitation at 355 nm and ~480 nm is provided in **Table S10**.

Table S10. Comparison of population yields Φ of $^{3*}[Fe(L^{PhAn})_2]^+$ in different solvents.

solvent	Φ(^{3*}FePhAn) visble light excitation	Φ(^{3*}FePhAn) UV light excitation
Acetonitrile	0.05	0.18±0.01
Dichloromethane	0.75±0.01	0.72±0.02

ns-TA-Spectroscopy: $\Phi_{3*FePhAn}$ of $[Fe(L^{PhAn})_2]^+$ in Acetonitrile

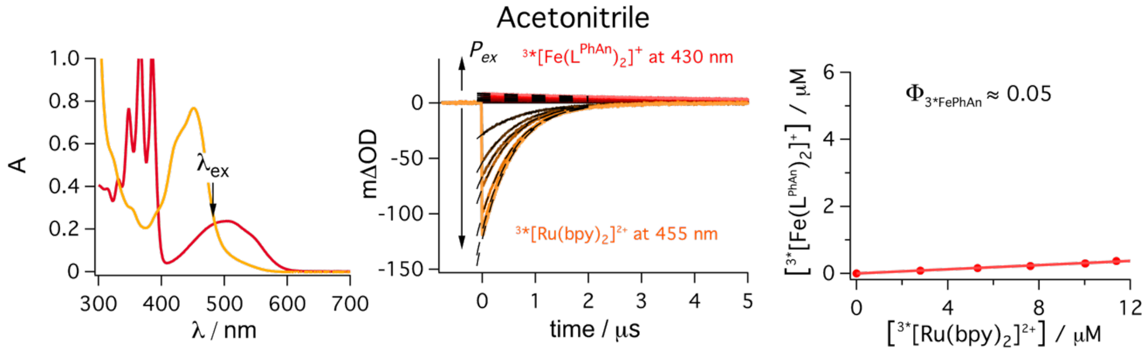

Figure S29. Triplet formation yield $F_{3*FePhAn}$ for $[Fe(L^{PhAn})_2]^+$ in acetonitrile after **visible** light excitation (l_{ex} = 485 nm). The values are determined against an isoabsorptive solution of $[Ru(bpy)_3]^{2+}$ in acetonitrile (left panel). Kinetic absorption traces of $[Fe(L^{PhAn})_2]^+$ (red traces) and $[Ru(bpy)_3]^{2+}$ (orange traces) in de-aerated solvent were detected at 430 nm and 455 nm respectively (middle panel). With the known exctinction coefficients the concentration of both solutions is determined and the triplet formation quantum yield is determined (right panel). The exact value calculates to a triplet yield $F_{3*FePhAn}$ of 0.03±0.01, but due to the very low signal intensity under these conditions a value of ~0.05 is estimated. Futher information is discussed in the text and the main manuscript.

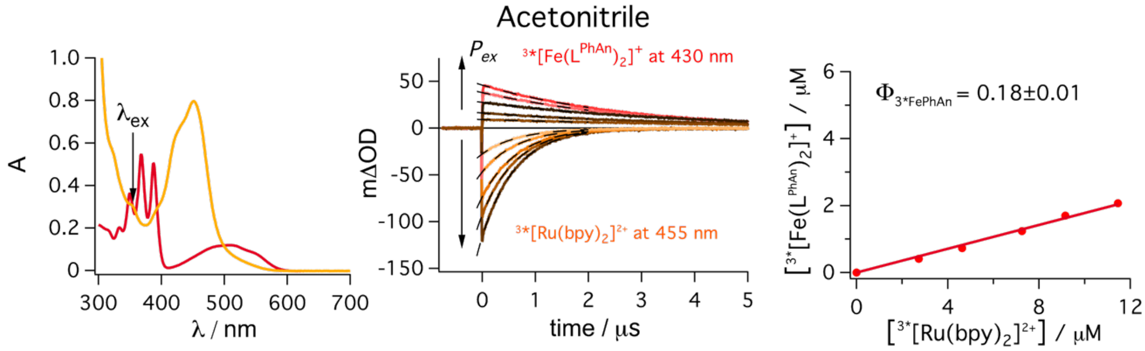

Figure S30. Triplet formation yield $F_{3*FePhAn}$ for $[Fe(L^{PhAn})_2]^+$ in acetonitrile after **UV** light excitation (l_{ex} = 355 nm). The values are determined against an isoabsorptive solution of $[Ru(bpy)_3]^{2+}$ in acetonitrile (left panel). Kinetic absorption traces of $[Fe(L^{PhAn})_2]^+$ (red traces) and $[Ru(bpy)_3]^{2+}$ (orange traces) in de-aerated solvent were detected at 430 nm and 455 nm respectively (middle panel). With the known exctinction coefficients the concentration of both solutions is determined and the triplet formation quantum yield is determined (right panel). Futher information is discussed in the text and the main manuscript.

ns-TA-Spectroscopy: $\Phi_{3*FePhAn}$ of $[Fe(L^{PhAn})_2]^+$ in Dichloromethane

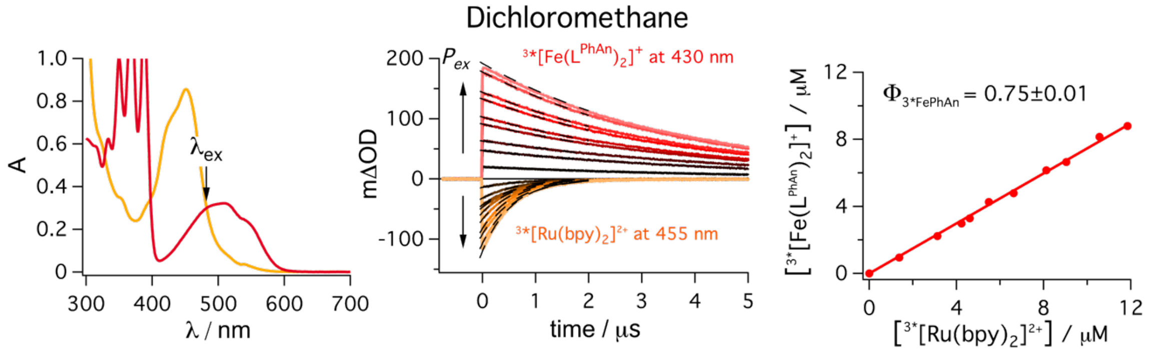

Figure S31. Triplet formation yield $F_{3*FePhAn}$ for $[Fe(L^{PhAn})_2]^+$ in dichloromethane after **visible** light excitation (l_{ex} = 482 nm). The values are determined against an isoabsorptive solution of $[Ru(bpy)_3]^{2+}$ in acetonitrile (left panel). Kinetic absorption traces of $[Fe(L^{PhAn})_2]^+$ (red traces) and $[Ru(bpy)_3]^{2+}$ (orange traces) in de-aerated solvent were detected at 430 nm and 455 nm respectively (middle panel). With the known exctinction coefficients the concentration of both solutions is determined and the triplet formation quantum yield is determined (right panel). Futher information is discussed in the text and the main manuscript.

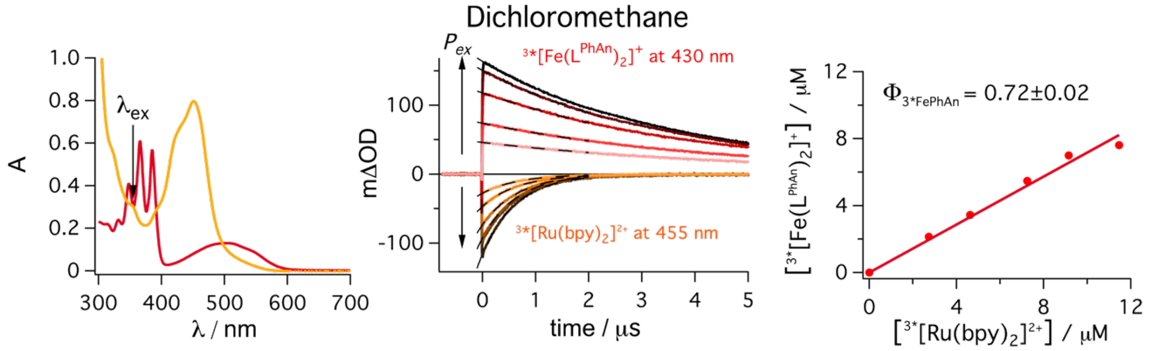

Figure S32. Triplet formation yield $F_{3*FePhAn}$ for $[Fe(L^{PhAn})_2]^+$ in dichloromethane after **UV** light excitation (l_{ex} = 355 nm). The values are determined against an isoabsorptive solution of $[Ru(bpy)_3]^{2+}$ in acetonitrile (left panel). Kinetic absorption traces of $[Fe(L^{PhAn})_2]^+$ (red traces) and $[Ru(bpy)_3]^{2+}$ (orange traces) in de-aerated solvent were detected at 430 nm and 455 nm respectively (middle panel). With the known exctinction coefficients the concentration of both solutions is determined and the triplet formation quantum yield is determined (right panel). Futher information is discussed in the text and the main manuscript.

ns-TA-Spectroscopy of [Fe(L^{PhAn})$_2$]$^+$ in Other Solvents

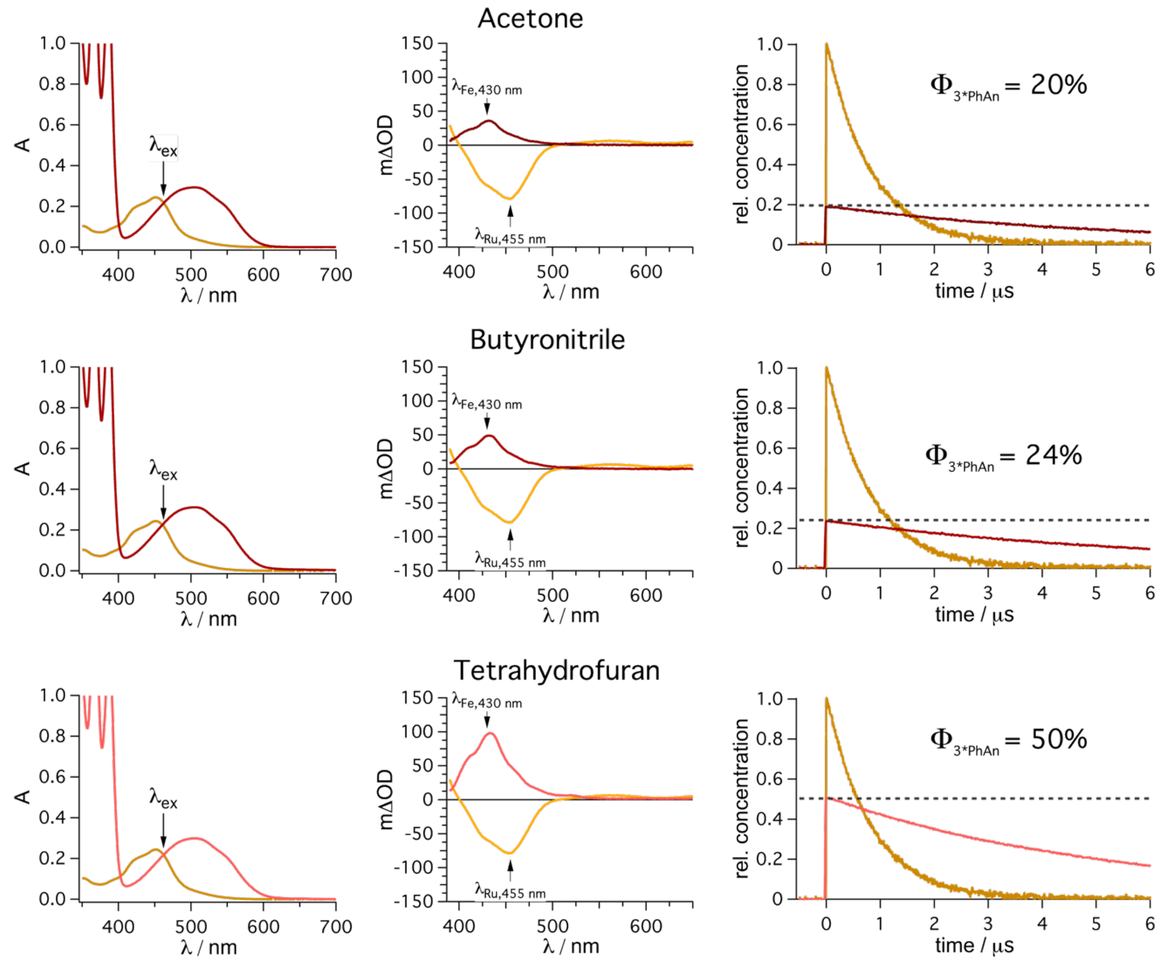

Figure S33. Triplet formation yield F_{3*PhAn} for [Fe(L^{PhAn})$_2$]$^+$ in acetone (top), butyronitrile (middle) and tetrahydrofuran (bottom). The values are determined against an isoabsorptive solution of [Ru(bpy)$_3$]$^{2+}$ in acetonitrile (left panels). Time-resolved transient absorption spectra of [Fe(L^{PhAn})$_2$]$^+$ and [Ru(bpy)$_3$]$^{2+}$ in de-aerated solvent measured 10 ns after the laser excitation at 470 nm (~10 mJ/pulse), time-integrated over 200 ns are presented in the middle panels and the relative concentrations based on the kinetic decay monitored at 430 nm for ^{3*}[Fe(L^{PhAn})$_2$]$^+$ (red traces) and at 455 nm for [Ru(bpy)$_3$]$^{2+}$ (orange traces) together with the estimated triplet formation quantum yield is provided in the right panel. Futher information is discussed in the text and the main manuscript.

3.4.3. ns-TA-Spectroscopy: Salt-Dependent ^{3*}PhAn for Bimolecular Quenching

As described in the main manuscript, a detection of a ^{2}CSS is prevented for the bimolecular quenching of $[Fe(L^{Ph})_2]^+$ by PhAn due to the presumably short-lived intermediate and insufficient quenching to allow its spectroscopic identification. Hence, an indirect method to provide futher insights was utilised, namely, the addition of *N*-tetrabutylammonium hexafluorophosphate ($TBAPF_6$) to change the dielectric constant in the solution. While a electron transfer cascade *via* two electron transfer steps should be dependent on the relative permittivity of the solution, a doublet-triplet energy transfer should be less sensitive to these changes. Indeed, upon additon of increasing concentrations of $TBAPF_6$ a decrase in the signal intensity of the transient absoprtion trace at 430 nm is visible (**Figure S34**), which results in a decrease of ~10% after correction for changes of absorbed photons and the change in emission quenching efficiency. This result is indeed in line with the expected change for the occurance of a ^{2}CSS also in the case of bimolecular quenching. This result has to be taken with care, as several parameters might not necessarily be constant upon salt addition (e.g. triplet molar absorption coefficients, preassociation equillibrium, ^{2}LMCT population and lifetime) and a direct spectroscopic detection of ^{2}CSS would be desirable.

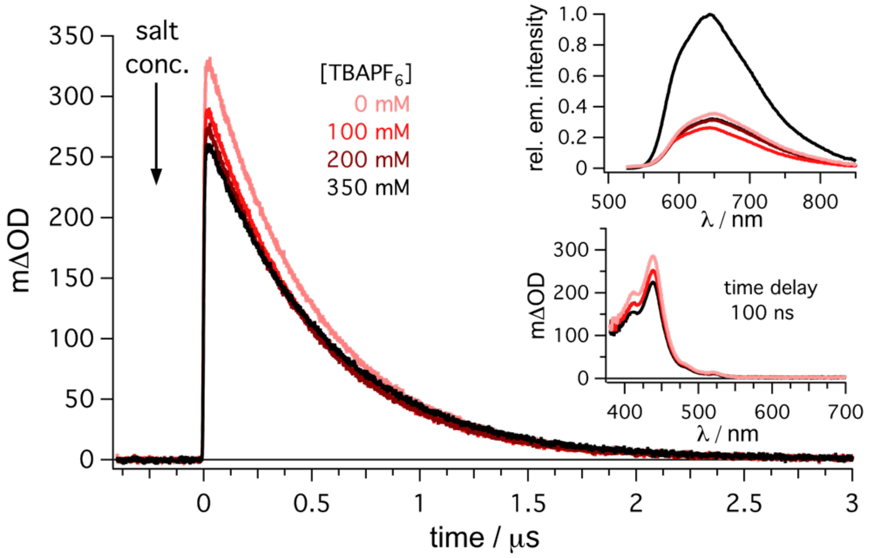

Figure S34. Relative triplet concentration monitored at 430 nm for $[Fe(L^{Ph})_2]^+$ (100 mM) in th presence of 100 mM of PhAn and various concentrations of *N*-tetrabutylammonium hexafluorophosphate ($TBAPF_6$) in aerated dichloromethane after laser excitation at 470 nm (~10 mJ/pulse). Relative emission intensities and time-resolved transient absorption spectra measured 100 ns after the laser excitation at 470 nm, time-integrated over 200 ns for the same solutions are presented as insets. The emission intensity is scaled to $[Fe(L^{Ph})_2]^+$ (100 mM, black trace) in the absence of salt and PhAn. All traces were corrected for small changes in the relative absorption at the excitation wavelength.

4. Additional Calculations

Reorganization energies based on Dielectric Continuum Theory

Reorganisation energies λ_0 are calculated based on equation 5 of the main manuscript, using the vacuum permettivity ε_0, the radii of the donor r_D and acceptor r_A and the gemometric distance R_{DA} (estimated as r_D+r_A), the solvent optical dielectric constant ε_{op} (= square of refractive index n_D of the solvent) and the solvent dielectric constant ε_r.[22] The respective values used for the different solvents and the results are summarised in **Table S11**. Equation 5 of the main manuscript is reprinted here as equation 3 for clarity.

$$\lambda_o = \frac{e^2}{4\pi\varepsilon_o}\left(\frac{1}{2r_D} + \frac{1}{2r_A} - \frac{1}{R_{DA}}\right)\left(\frac{1}{\varepsilon_{op}} - \frac{1}{\varepsilon_r}\right) \qquad \text{Eq. 3}$$

Table S11. Overview of the reorganization energies λ_0 calculated in different solvents.

solvent	n_D	ε_{op}	ε_r	λ_0 / eV [a]
Acetonitrile	1.334 [15]	1.780	35.9 [15]	1.03
Acetone	1.359 [15]	1.849	20.7 [15]	0.95
Butyronitrile	1.384 [23]	1.915	20.3 [24]	0.91
Tetrahydrofuran	1.407 [15]	1.980	7.58 [15]	0.72
Dichloromethane	1.424 [15]	2.028	8.93 [15]	0.73

[a] Calculated using equation 3 with e = 1.602 10^{-19} C, r_A= 3.2 Å, r_D = 5.4 Å, R_{DA} = 8.6 Å, ε_0 = 8.854 10^{-12} $CJ^{-1}m^{-1}$, and ε_{op} and ε_r for the individual solvents (see text for additional details).

5. Bibliography

(1) Glaser, F.; De Kreijger, S.; Troian-Gautier, L., Two Birds, One Stone: Microsecond Dark Excited-State Lifetime and Large Cage Escape Yield Afforded by an Iron–Anthracene Molecular Dyad. *J. Am. Chem. Soc.* **2025**, *147*, 8559–8567.

(2) Maubert, B.; McClenaghan, N. D.; Indelli, M. T.; Campagna, S., Absorption Spectra and Photophysical Properties of a Series of Polypyridine Ligands Containing Appended Pyrenyl and Anthryl Chromophores and of Their Ruthenium(II) and Osmium(II) Complexes. *J. Phys. Chem. A* **2003**, *107*, 447–455.

(3) Tyson, D. S.; Henbest, K. B.; Bialecki, J.; Castellano, F. N., Excited State Processes in Ruthenium(II)/Pyrenyl Complexes Displaying Extended Lifetimes. *J. Phys. Chem. A* **2001**, *105*, 8154–8161.

(4) Ford, W. E.; Rodgers, M. A. J., Reversible Triplet-Triplet Energy Transfer within a Covalently Linked Bichromophoric Molecule. *J. Phys. Chem.* **1992**, *96*, 2917–2920.

(5) Wellauer, J.; Pfund, B.; Becker, I.; Meyer, F.; Prescimone, A.; Wenger, O. S., Iron(III) Complexes with Luminescence Lifetimes of up to 100 Ns to Enhance Upconversion and Photocatalysis. *J. Am. Chem. Soc.* **2025**, *147*, 8760–8768.

(6) Campagna, S.; Genovese, S.; Arrigo, A., Fe(III) Complexes with Prolonged Luminescence Lifetimes and Symmetry-Breaking Charge Separation. *Res. Sq.* **2024**.

(7) Kim, D.; Rosko, M. C.; Castellano, F. N.; Gray, T. G.; Teets, T. S., Long Excited-State Lifetimes in Three-Coordinate Copper(I) Complexes via Triplet-Triplet Energy Transfer to Pyrene-Decorated Isocyanides. *J. Am. Chem. Soc.* **2024**, *146*, 19193–19204.

(8) Glaser, F.; Schmitz, M.; Kerzig, C., Coulomb Interactions for Mediator-Enhanced Sensitized Triplet–Triplet Annihilation Upconversion in Solution. *Nanoscale* **2024**, *16*, 123–137.

(9) Van Stokkum, I. H. M.; Larsen, D. S.; Van Grondelle, R., Global and Target Analysis of Time-Resolved Spectra. *Biochim. Biophys. Acta - Bioenerg.* **2004**, *1657*, 82–104.

(10) Mullen, K. M.; Stokkum, I. H. M. van., TIMP : An R Package for Modeling Multi-Way Spectroscopic Measurements. *J. Stat. Softw.* **2007**, *18*, 1–46.

(11) Snellenburg, J. J.; Laptenok, S. P.; Seger, R.; Mullen, K. M.; Stokkum, I. H. M. van., Glotaran : A Java -Based Graphical User Interface for the R Package TIMP. *J. Stat. Softw.* **2012**, *49*, 1–22.

(12) Suzuki, K.; Kobayashi, A.; Kaneko, S.; Takehira, K.; Yoshihara, T.; Ishida, H.; Shiina,

Y.; Oishi, S.; Tobita, S., Reevaluation of Absolute Luminescence Quantum Yields of Standard Solutions Using a Spectrometer with an Integrating Sphere and a Back-Thinned CCD Detector. *Phys. Chem. Chem. Phys.* **2009**, *11*, 9850–9860.

(13) Kerzig, C.; Goez, M., Combining Energy and Electron Transfer in a Supramolecular Environment for the "Green" Generation and Utilization of Hydrated Electrons through Photoredox Catalysis. *Chem. Sci.* **2016**, *7*, 3862–3868.

(14) Müller, P.; Brettel, K., $[Ru(bpy)_3]^{2+}$ as a Reference in Transient Absorption Spectroscopy: Differential Absorption Coefficients for Formation of the Long-Lived ^{3}MLCT Excited State. *Photochem. Photobiol. Sci.* **2012**, *11*, 632–636.

(15) Montalti, M.; Credi, A.; Prodi, L.; Gandolfi, M. T., *Handbook of Photochemistry*, 3rd ed.; CRC Press: Taylor & Francis Group: Boca Raton, FL, USA, 2006.

(16) Horrocks, A. R.; Medinger, T.; Wilkinson, F., Solvent Dependence of the Quantum Yield of Triplet State Production of 9-Phenylanthracene. *Photochem. Photobiol.* **1967**, *6*, 21–28.

(17) Horrocks, A. R.; Kearvell, A.; Tickle, K.; Wilkinson, F., Mechanism of Fluorescence Quenching in Solution. Part 2.—Quenching by Xenon and Intersystem Crossing Efficiencies. *Trans. Faraday Soc.* **1966**, *62*, 3393–3399.

(18) Birks, J. B., *Photophysics of Aromatic Molecules*; John Wiley & Sons Ltd, 1970.

(19) Medinger, T.; Wilkinson, F., Mechanism of Fluorescence Quenching in Solution. Part 1. - Quenching by Bromobenzene. *Trans. Faraday Soc.* **1965**, *61*, 620–630.

(20) Jin, P.; Xu, X.; Yan, Y.; Hammecke, H.; Wang, C., Luminescent Fe(III) Complex Sensitizes Aerobic Photon Upconversion and Initiates Photocatalytic Radical Polymerization. *J. Am. Chem. Soc.* **2024**, *146*, 35390–35401.

(21) Weller, A., Photoinduced Electron Transfer in Solution: Exciplex and Radical Ion Pair Formation Free Enthalpies and Their Solvent Dependence. *Zeitschrift fur Phys. Chemie* **1982**, *133*, 93–98.

(22) Piechota, E. J.; Meyer, G. J., Introduction to Electron Transfer: Theoretical Foundations and Pedagogical Examples. *J. Chem. Educ.* **2019**, *96*, 2450–2466.

(23) Daragan, B., Recherches Réfractométriques Dans La Série Des Nitriles Saturés Normaux. *Bull. des Sociétés Chim. Belges* **1935**, *44*, 597–624.

(24) Van Duyne, R. P.; Reilley, C. N., Low-Temperature Electrochemistry I. Characteristics of Electrode Reactions in the Absence of Coupled Chemical Kinetics. *Anal. Chem.* **1972**, *44*, 142–152.
