# Supplementary material for: Beyond Common Energy Transfer: Intramolecular Electron Transfer Cascade Controls Triplet Population of a Long-Lived Iron-Anthracene Molecular Dyad
Source: ACS Cent Sci. 2025 Aug 4;11(10):1870–81. doi: 10.1021/acscentsci.5c01040 (PMC12550628; doi:10.1021/acscentsci.5c01040)
Supplement: Supplementary file 2 [file oc5c01040_si_002.pdf]

oc-2025-010402.R1

Name: Peer Review Information for "Beyond Common Energy Transfer: Intramolecular Electron Trans-fer Cascade Controls Triplet Population of a Long-Lived Iron-Anthracene Molecular Dyad"

First Round of Reviewer Comments

Reviewer: 1

Comments to the Author

The present manuscript reports on the population of anthracene's triplet state in a dyad containing a Fe(III) complex, mediated through a cascade electron transfer mechanism. This mechanism enables control over the final triplet yield by tuning the solvent polarity.

The manuscript is clearly written and presented in a highly didactic manner, making it accessible even to readers who are not specialists in photochemistry, despite the complex spectroscopic characterization involved. As the authors point out, this work has the potential to significantly impact the field of first-row transition metal complexes by offering new pathways for the formation of long-lived excited states.

The paper is acceptable in its current form. I would only suggest that the authors carefully review the Supporting Information for minor errors. For example, in Figure S2, "THF" is incorrectly written as tetrahydrofurane, and on page S10, the first sentence has misspelled words: <Excitation spectra confimred that the remianing emission in our dyads are caused by the iron based emision from the 2LMCT>

Reviewer: 2

Comments to the Author

Glaser et al present a thorough study following up on their initial report of an iron-anthracene hybrid that exhibits an exceptionally long (microsecond), non-luminescent excited state lifetime. In that initial work, they made an intriguing observation that despite the long-lived triplet excited state, light-absorption generates a rather low triplet population yield. Here, through careful spectroscopic studies, they are able to delineate a novel decay cascade in which intramolecular electron transfer precedes formation of the ultimate triplet state. These insights are then exploited to improve triplet yields to 75% by judicious choice of solvent.

This is an important finding. As the authors note in their introduction, the combination of iron chromophores with organic triplet reservoirs has been gaining steam of late, thanks to the possibility to generate unprecedented excited state long lifetimes for iron-containing complexes. Starting from ferric d5 electronic configurations that can access spin-conserved 2LMCT excitation, long-lived, often luminescent 2LMCT excited states can be generated that have been proposed to populate triplet excited states through doublet-triplet energy transfer.

The identification of DTET relied on robust characterization of the luminescent 2LMCT state and the signature absorption of the triplet state of the organic moiety via transient absorption spectroscopy. But the issue required to overcome was the incomplete population of the triplet state. Improving this metric from the observed ~10% could greatly influence the potential utility of these new chromophore designs – precisely what is achieved here. I really enjoyed this paper and feel the findings and careful work will undoubtedly benefit the research community and interest the broad readership of ACS Central Science as the search for sustainable photoactive compounds continues to deepen. Nonetheless, I do feel some revision is warranted and provide some suggestions below. One major comment is that the observation of an (Fe-based) impurity noted in the SI could be addressed a bit more. The authors state that “Taking all spectroscopic data and analysis into account, the impurity is not expected to have a noticeable influence on the findings and conclusions of our study”; I wonder if (in the SI), the authors could elaborate a bit more on this to assuage any doubts.

Overall, this is an excellent, important study and I hope the comments below help to strengthen this already fine work.

Page 1, line 17 – “that a the”

Page 1, line 25 – “Although the final triplet energy level is important…” Do you mean to say the energy of the ‘final triplet state’?

Figure 2 – there almost appears to be two maxima to the DCM emission profiles for both species; in addition, the TCSPC decay for the dyad looks like it has two temporal regimes and rates. Can the authors comment, given the differences subsequently identified in DCM by fsTA? Table S2 clarifies this in a footnote “For solvents with significant intramolecular excited-state quenching and consequently short emission lifetimes a biexponential emission decay is observed, of which the shorter emission lifetimes is assigned to the [Fe(LPhAn)2]+ and the long-lived component with lifetime of ~2 ns is assigned to an iron-based impurity.” I wonder if this warrants noting in the main text. Do the authors have any idea of the nature of the impurity?

Figure 3b – Why would the 2LMCT have a nearly twice as long time constant at higher energy excitation compared with 500 nm excitation? I’m curious as to why the population of the 2CSS doesn’t seem to peak in the same sequential manner as at lower energy excitation.

Page 4, left column line 25 – “From an energetic viewpoint, this sequence is downhill in DCM as the 2CSS (~1.90 eV) is energetically in between the 2LMCT (2.15 eV) and 3*PhAn (~1.8 eV).” It would be informative for the reader to state at this point how these values were each determined/estimated, or at least to point the reader to subsequent sections.

Page 4, right column, line 2- “The contribution of PhAn ground-state absorptions between 350 and 390 nm (Figure 1)14 allowed an almost exclusive excitation (>90%) of the polyaromatic hydrocarbon” Figure 2b?

Page 5, left column, line 4 – “Unfortunately, electrochemical measurements were frustrated by irreversible redox chemistry in some solvents (SI section 2.11),” Section 2.1.1?

Page 5, left column, line 18 – “Qualitatively, a clear correlation between the remaining emission lifetime from the 2LMCT state” remaining?

Page 5, left column, line 44 – “Thus, to estimate ECSS,53–55 the oxidation potential of PhAn (Eox) and the reduction potential of the iron center (Ered) where used, in combination to DGS that was evaluated in different solvents using Eq. 2.56” were used… in combination with… that were…

Page 5, left column, line 52 – “With the reduction potential (Ered,Fe = –0.74 V vs SCE) and the oxidation potential (Eox,PhAn= 1.35 V vs SCE) the energy of the charge separated state and the Born correction DGS can be calculated.” In which solvent are these redox potentials referring to? Checking against Table S5…

Page 5, right column, line 9 – “While the absolute value of ECSS might not be perfectly accurate, the overall trend between shorter emission lifetimes of 2LMCT and larger driving force for the electron transfer to generate [(LPhAn)FeII(LPhAn•+)]+ is clearly visible (Table 1).” Is the sense here that the THF vs DCM comparison is anomalous because of the irreversibility of the PhAn oxidation in THF that hampers accurate determination of Eox(PhAn)?

THF vs DCM is again anomalous re: triplet yields - Page 6, left column, line 3 – “Triplet formation efficiency only reached 0.05 in a MeCN, whereas it increased to 0.20 in acetone, 0.25 in butyronitrile, 0.50 in tetrahydrofuran and reached an impressive value of 0.75 in DCM.”

Page 5, right column, line 52 – “Focusing again on the nanosecond timescale, several microseconds of excited-state lifetime are detectable for the dyad in all investigated solvents (SI section 3.3.1)." Just checking – this is just in DCM and ACN, correct? Despite

measuring the fluorescence lifetime of the dyad in all the solvents listed in Table 1, TA was only performed in the two solvents ACN and DCM and this is what is referred to here.

Page 6, left column, line 10 – "An interesting aspect of this analysis is the fact that, in DCM, essentially all of the ~80% 2LMCT excited-state quenching (2.45 ns vs 0.35 ns) results in a long-lived triplet state while in MeCN, the one third shorter excited-state lifetime is not reflected in the final triplet yield (0.05)." I'm intrigued by the fact that in DCM the oxidation of PhAc is the most reversible. While the timescales are clearly different, the stability of the oxidized 2PhAn radical in DCM may play a role and be worth a comment. See below re: the idea of providing normalized relative populations that reflect the actual comparable populations of the states observed by TA.

Page 6, left column – "Gratefully, from the fs-TA analysis in both solvents using 365 nm excitation, where the photons are largely absorbed by the anthracene moiety, the populated singlet state is only present for around 5 ps. This enables a different pathway for the formation of the CSS within the dyad to obtain further insights in both solvents." I'm not sure I follow what is being suggested here. Could the second sentence be amended for more clarity?

Page 6, right column – "In MeCN only 0.18 of triplet were formed using 360 nm irradiation, pointing towards a significant contribution of unproductive charge recombination in MeCN." I'm trying to reconcile this observation with the inset of figure 3a (365 nm excitation). If a large proportion of the CSS state was formed but then converting to the GS without forming the 3PhAc state, wouldn't this be reflected in the relative populations measured by TA? Or are these 'relative populations' not relative to each other but rather normalized each to itself?

Page 6, right column, line 26 – "or changes is geometry" in geometry?

Page 7, right column, line 34 – "dexter energy" Dexter energy transfer?

Page 7, right column, line 37 – “without the need for a formal spin change” without the need for formal overall spin change? Also, “thus decreasing potential energy loss channels.” Isn’t the argument rather that the productive path is more efficient? What are the missing energy loss channels?

SUPPORTING INFORMATION

Page S10 – “2.1.1. (Spectro)electrochemical Characteristization”

Figure S12 caption – “Small differnces”

Page S18 – “In addition to the comparsion”

Page S31 – “while in acetone, butyronitrile, and butyronitrile the values were measured”

Reviewer: 3

Comments to the Author

This manuscript describes the solvent dependence of the excited state dynamics of a long lived Fe(III)–anthracene dyad. The work is expertly done and the manuscript is well written. I suspect that this paper will be of great interest to the photochemical community.

I recommend acceptance of the manuscript in its present form. However, the authors might decide to include a brief paragraph (or a few sentences) that relates their discussion of the work term and the driving force for the formation of the CSS to the classical reorganization energy in Marcus theory. It is hard to imagine that the reorganization energy is the same in both solvents. I think such a description would improve the manuscript.

Reviewer: 4

Comments to the Author

This paper follows a recent publication in JACS on the same compound, where the focus was on the population of an anthracene-localized triplet excited state after excitation of the iron(III) complex into its lowest 2LMCT state (reference 14). From what I gather, the previous JACS article concentrated on the decay behavior, specifically the long lifetime, of this dark triplet state on anthracene. In contrast, the current manuscript now shifts focus to the formation of this triplet state.

The experimental evidence supporting the mechanistic proposal presented in Figure 5b is, in my view, convincing. The relevant spectral signatures are directly observable, and the kinetics clearly reflect state-to-state transitions: the disappearance of one species coincides with the appearance of the expected follow-up product.

To my knowledge, the mechanism proposed in Figure 5b has not been directly observed before. While the case of low-spin d6 systems shown in Figure 5a is well established and conceptually analogous in some respects, there is a notable conceptual difference. Specifically, the mechanism in Figure 5b provides a direct pathway from a doublet excited state to a triplet excited state without the involvement of an intersystem crossing (ISC) event. This was new to me, and I find it particularly intriguing.

In my opinion, the paper would benefit from a broader discussion of the relevance of this mechanism. Why has this type of transition not been observed previously, despite the existence of comparable systems in the literature, such as iron(III) photosensitizers combined with polyaromatic hydrocarbons, whether in bimolecular assemblies or covalently linked unimolecular systems? Furthermore, extrapolating from this somewhat greater context going beyond the author's own work, how might this mechanism impact the future of photochemistry? What is its particular significance for the photochemistry of first-row transition metals in other oxidation states or valence electron configurations?

Including this broader discussion would, in my view, help the editor better assess the importance and potential impact of the work presented here, especially in relation to the recently published JACS paper on the same compound.

Author's Response to Peer Review Comments:

Editorial comments : Formatting Needs:

- Abstract: Please make sure the word count of your Abstract does not exceed 200 words.

Response: The abstract was now reduced from 250 words to 200 words.

- Author Affiliations: Please move author affiliations to the first page of the manuscript under the author list. Please use separate labels for each affiliation next to the authors' names.

Response: The authors affiliations were moved to the first page of the manuscript.

- Author List: Please include the email address(es) of the corresponding author(s) on the first page of the manuscript.

Response: E-mail addresses of the corresponding authors were added on the first page.

- Major Objects: Please cite each major object (figures, tables, equations and schemes) in the manuscript text. Figure 4 does not appear to be cited.

Response: Figure 4 is now cited at the appropriate place.

- Supporting Information: Please add a full header to the top of the file designated "Supporting
  Information for Publication." Provide the title (in title case), authors' names, and affiliations on the top of the first page, matching those of the manuscript file exactly.

Response: The header "Supporting Information for Publication" was added and we verified that the title, authors' names and affiliations matched those of the manuscript. .

- Synopsis: ACS Central Science requires a brief synopsis. The synopsis should be no more than 200 characters (including spaces) and should reasonably correlate with the Table of Contents (TOC) graphic. The synopsis is intended to explain the

importance of the article to a broader readership across the sciences. Please place your synopsis in the manuscript file after the TOC graphic and label as “Synopsis.”

Response: The synopsis was added at the end of the manuscript after the TOC graphic.

Reviewer: 1

Recommendation: Publish in ACS Central Science without change.

Comments:
The present manuscript reports on the population of anthracene’s triplet state in a dyad containing a Fe(III) complex, mediated through a cascade electron transfer mechanism. This mechanism enables control over the final triplet yield by tuning the solvent polarity.

The manuscript is clearly written and presented in a highly didactic manner, making it accessible even to readers who are not specialists in photochemistry, despite the complex spectroscopic characterization involved. As the authors point out, this work has the potential to significantly impact the field of first-row transition metal complexes by offering new pathways for the formation of long-lived excited states.

The paper is acceptable in its current form. I would only suggest that the authors carefully review the Supporting Information for minor errors. For example, in Figure S2, “THF” is incorrectly written as tetrahydrofurane, and on page S10, the first sentence has misspelled words: <Excitation spectra confimred that the remianing emission in our dyads are caused by the iron based emision from the 2LMCT>

Response: We thank the reviewer for their feedback and kind words. We have now proofread the manuscript and the supporting information to catch the spelling mistakes that were still present.

Additional Questions:

Quality of experimental data, technical rigor: Top 1%
Significance to chemistry researchers in this and related fields: Top 10%
Broad interest to other researchers: Top 10%
Novelty: Top 1%
Is this research study suitable for media coverage or a First Reactions (a News & Views piece in the journal)?: Yes Reviewer: 2

Recommendation: Publish in ACS Central Science after minor revisions noted.

Comments:

Glaser et al present a thorough study following up on their initial report of an iron-anthracene hybrid that exhibits an exceptionally long (microsecond), non-luminescent excited state lifetime. In that initial work, they made an intriguing observation that despite the long-lived triplet excited state, light-absorption generates a rather low triplet population yield. Here, through careful spectroscopic studies, they are able to delineate a novel decay cascade in which intramolecular electron transfer precedes formation of the ultimate triplet state. These insights are then exploited to improve triplet yields to 75% by judicious choice of solvent.

This is an important finding. As the authors note in their introduction, the combination of iron chromophores with organic triplet reservoirs has been gaining steam of late, thanks to the possibility to generate unprecedented excited state long lifetimes for iron-containing complexes. Starting from ferric d5 electronic configurations that can access spin-conserved 2LMCT excitation, long-lived, often luminescent 2LMCT excited states can be generated that have been proposed to populate triplet excited states through doublet-triplet energy transfer.

The identification of DTET relied on robust characterization of the luminescent 2LMCT state and the signature absorption of the triplet state of the organic moiety via transient absorption spectroscopy. But the issue required to overcome was the incomplete population of the triplet state. Improving this metric from the observed ~10% could greatly influence the potential utility of these new chromophore designs – precisely what is achieved here. I really enjoyed this paper and feel the findings and careful work will undoubtedly benefit the research community and interest the broad readership of ACS Central Science as the search for sustainable photoactive compounds continues to deepen. Nonetheless, I do feel some revision is warranted and provide some suggestions below. One major comment is that the observation of an (Fe-based) impurity noted in the SI could be addressed a bit more. The authors state that "Taking all spectroscopic data and analysis into account, the impurity is not expected to have a noticeable influence on the findings and conclusions of our study"; I wonder if (in the SI), the authors could elaborate a bit more on this to assuage any doubts.

Overall, this is an excellent, important study and I hope the comments below help to strengthen this already fine work.

Response: We would like to thank the review for their feedback and kind words. We have now elaborated on the identity of the impurity in the manuscript as well as in the supporting information. Whereas we were unable to isolate this impurity, analytical HPLC analysis with UV-Visible detection seems to indicate that the

impurity is a complex bearing only one anthracene moiety, i.e. a heteroleptic complex. It is reasonable to assume that the LMCT excitation of that photosensitizer is split between 50% from the ligand bearing the anthracene moiety (probably leading to the desired ^{3}PhAn unit) and 50% from the unfunctionalized ligand, leading to the standard 2 ns photoluminescent lifetime of the photosensitizer. This observation is extremely interesting and we are targeting to isolate this impurity by preparative HPLC (when the technique becomes available in the lab hopefully by the end of the year) and investigate its excited-state properties and ^{3}PhAn population.

Page 1, line 17 – “that a the”

Response: This typo was now corrected.

Page 1, line 25 – “Although the final triplet energy level is important...” Do you mean to say the energy of the ‘final triplet state’?

Response: This typo was now corrected.

Figure 2 – there almost appears to be two maxima to the DCM emission profiles for both species; in addition, the TCSPC decay for the dyad looks like it has two temporal regimes and rates. Can the authors comment, given the differences subsequently identified in DCM by fsTA? Table S2 clarifies this in a footnote “For solvents with significant intramolecular excited-state quenching and consequently short emission lifetimes a biexponential emission decay is observed, of which the shorter emission lifetimes is assigned to the [Fe(LPhAn)2]+ and the long-lived component with lifetime of ~2 ns is assigned to an iron-based impurity.” I wonder if this warrants noting in the main text. Do the authors have any idea of the nature of the impurity?

Response: The emission profile recorded in DCM for both complexes exhibits two maxima, or a maxima and a shoulder. For the unsubstituted complex, this is in agreement with reports in the literature, both from our group as well as from the group of K. Wärnmark and co-workers. This is in line with the slight solvatochromism of this complex, previously studied by the two above-mentioned groups. However, for the dyad in dichloromethane, excited-state lifetime measurements show a very short-lived component (corresponding to the excited-state quenching via electron transfer) and a longer-lived component of ~2ns that has the same properties on the unsubstituted complex. At first, we thought that traces of unsubstituted complex were present, but extensive purifications and characterization by analytical HPLC analysis showed that the impurity is a complex bearing only one anthracene moiety, i.e. a heteroleptic

complex. In dichloromethane, we believe that the remaining ~2ns component originated from this complex. It is reasonable to assume that the LMCT excitation of that photosensitizer is split between 50% from the ligand bearing the anthracene moiety (probably leading to the desired ^{3}PhAn unit) and 50% from the unfunctionalized ligand, leading to the standard 2 ns photoluminescent lifetime of the photosensitizer. This is of course just a hypothesis and we would like to investigate this particular properties as soon as possible. Nevertheless, given the small fraction of impurity and the extensive characterization that we have performed, we believe that the impurity has no impact on the mechanism and analysis reported herein.

Figure 3b – Why would the 2LMCT have a nearly twice as long time constant at higher energy excitation compared with 500 nm excitation? I'm curious as to why the population of the 2CSS doesn't seem to peak in the same sequential manner as at lower energy excitation.

Response: The time constant for the ^{2}LMCT at 365 nm irradiation (170 ps) and at 500 nm (99 ps) are still in close agreement with each other considering experimental uncertainties. This slight difference might originate from the sequential model population the 2LMCT from the singlet PhAn populated upon 365 nm irradiation. The CSS has almost identical time constants between both irradiation wavelength, but the relative population appears slightly smaller. These differences are not uncommon in the modelling of ultrafast transient absorption spectroscopy data.

Page 4, left column line 25 – "From an energetic viewpoint, this sequence is downhill in DCM as the 2CSS (~1.90 eV) is energetically in between the 2LMCT (2.15 eV) and 3*PhAn (~1.8 eV)." It would be informative for the reader to state at this point how these values were each determined/estimated, or at least to point the reader to subsequent sections.

Response: We have now added a reference at the end of this sentence for the energy of the ^{2}LMCT state and the ^{3}PhAn state and directed the reader to a later section for the energy of the ^{2}CSS.

Page 4, right column, line 2- "The contribution of PhAn ground-state absorptions between 350 and 390 nm (Figure 1)14 allowed an almost exclusive excitation (>90%) of the polyaromatic hydrocarbon" Figure 2b?

Response: This was corrected and it is indeed Figure 2a and b.

Page 5, left column, line 4 – "Unfortunately, electrochemical measurements were frustrated by irreversible redox chemistry in some solvents (SI section 2.11)," Section 2.1.1?

Response: This was now corrected.

Page 5, left column, line 18 – "Qualitatively, a clear correlation between the remaining emission lifetime from the 2LMCT state" remaining?

Response: This word "remaining" was now deleted.

Page 5, left column, line 44 – "Thus, to estimate ECSS,53–55 the oxidation potential of PhAn (Eox) and the reduction potential of the iron center (Ered) where used, in combination to DGS that was evaluated in different solvents using Eq. 2.56" were used… in combination with… that were…

Response: This was now corrected.

Page 5, left column, line 52 – "With the reduction potential (Ered,Fe = –0.74 V vs SCE) and the oxidation potential (Eox,PhAn= 1.35 V vs SCE) the energy of the charge separated state and the Born correction DGS can be calculated." In which solvent are these redox potentials referring to? Checking against Table S5…

Response: These potentials were determined in acetonitrile, a detail that was added to the manuscript and they are indeed in line with table S5.

Page 5, right column, line 9 – "While the absolute value of ECSS might not be perfectly accurate, the overall trend between shorter emission lifetimes of 2LMCT and larger driving force for the electron transfer to generate [(LPhAn)FeII(LPhAn•+)]+ is clearly visible (Table 1)." Is the sense here that the THF vs DCM comparison is anomalous because of the irreversibility of the PhAn oxidation in THF that hampers accurate determination of Eox(PhAn)?

Response: Exactly. We prefer to be cautious rather than claiming "absolute" E_{CSS} and convey that irreversibility in electrochemistry creates a potential source of error. This was already described in the manuscript but we tried to make it even clearer and introduced aspects related to the triplet energy level that could also be affected by the change in solvent.

THF vs DCM is again anomalous re: triplet yields - Page 6, left column, line 3 – "Triplet formation efficiency only reached 0.05 in a MeCN, whereas it increased to 0.20 in acetone, 0.25 in butyronitrile, 0.50 in tetrahydrofuran and reached an impressive value of 0.75 in DCM."

Response: The reviewer is correct. Based on the E_{CSS}, we would expect the THF to provide larger ^{3}PhAn yields than DCM. Electrochemical irreversibility and inaccurate determination of E_{CSS} could be a reason. It would also be that the

ECSS level in THF is too close to the ^{3}PhAn energy, or slightly below, thereby decreasing the yield of ^{3}PhAn by making this process slightly uphill. Furthermore, our simplified assumption of the same triplet energy level in all solvents might not be fully accurate. These are aspects that we will continue to investigate in the future.

Page 5, right column, line 52 – "Focusing again on the nanosecond timescale, several microseconds of excitedstate lifetime are detectable for the dyad in all investigated solvents (SI section 3.3.1)." Just checking – this is just in DCM and ACN, correct? Despite measuring the fluorescence lifetime of the dyad in all the solvents listed in Table 1, TA was only performed in the two solvents ACN and DCM and this is what is referred to here.

Response: The excited-state lifetime by nanosecond transient absorption spectroscopy was measured for the dyad in the 5 solvents. These excited-state lifetimes are reported in the supporting information (Table S9).

Page 6, left column, line 10 – "An interesting aspect of this analysis is the fact that, in DCM, essentially all of the ~80% 2LMCT excited-state quenching (2.45 ns vs 0.35 ns) results in a long-lived triplet state while in MeCN, the one third shorter excited-state lifetime is not reflected in the final triplet yield (0.05)." I'm intrigued by the fact that in DCM the oxidation of PhAn is the most reversible. While the timescales are clearly different, the stability of the oxidized 2PhAn radical in DCM may play a role and be worth a comment. See below re: the idea of providing normalized relative populations that reflect the actual comparable populations of the states observed by TA.

Response: We did not observe any stability issue on the femtosecond timescale likely due to the short lifetime of the intermediate state, neither did we in our previous study that investigate $^1O_2$ formation and bimolecular excited-state electron transfer. Irreversibility or instability was only noted during our (spectro)electrochemical experiments, where oxidized phenyl-anthracene is present for significant longer timescales.

Page 6, left column – "Gratefully, from the fs-TA analysis in both solvents using 365 nm excitation, where the photons are largely absorbed by the anthracene moiety, the populated singlet state is only present for around 5 ps. This enables a different pathway for the formation of the CSS within the dyad to obtain further insights in both solvents." I'm not sure I follow what is being suggested here. Could the second sentence be amended for more clarity?

Response: We have modified the second sentence to read "Thermodynamically, population of 1*PhAn (Eox* = –1.85 V vs SCE) enables an oxidative quenching

pathway where 1*PhAn in oxidized by the FeIII center (E1/2,red = –0.74 V vs SCE), leading to the identical 2CSS."

Page 6, right column – "In MeCN only 0.18 of triplet were formed using 360 nm irradiation, pointing towards a significant contribution of unproductive charge recombination in MeCN." I'm trying to reconcile this observation with the inset of figure 3a (365 nm excitation). If a large proportion of the CSS state was formed but then converting to the GS without forming the 3PhAc state, wouldn't this be reflected in the relative populations measured by TA? Or are these 'relative populations' not relative to each other but rather normalized each to itself?

Response: We agree with the reviewer that the relative populations in our modelling did not reflect the triplet quantum yields. We have re-modelled the fs-TA data incorporating triplet quantum yields and electron transfer quantum yields when available (a 100% electron transfer efficiency was considered for 365 nm excitation). Importantly, differential spectral shapes and lifetimes after re-modelling are identical to those obtained before. The only change is an increased intensity for the differential spectra of triplet states, in line with the consideration of smaller populations. As we did not perform quantitative determination of the yield of CSS, we kept our initial presentation and added a clarifying sentence "Note that the relative populations does not account for unproductive return to the ground state from intermediates i.e. the overall sum of relative population always equals 1.***" in the SI to emphasize the fact that this is a relative population and not an absolute population.***

Page 6, right column, line 26 – "or changes is geometry" in geometry?

Response: This was now corrected.

Page 7, right column, line 34 – "dexter energy" Dexter energy transfer?

Response: This was now corrected.

Page 7, right column, line 37 – "without the need for a formal spin change" without the need for formal overall spin change? Also, "thus decreasing potential energy loss channels." Isn't the argument rather that the productive path is more efficient? What are the missing energy loss channels?

Response: We have corrected the first sentence to include the world "overall". Regarding the potential energy losses, we argued that ISC and related VR/IC processes can dissipate energy that could be prevented by this cascade of electron transfer. The electron cascade process renders this productive path

more efficient, but to some extend doublet-triplet energy transfer, if operative, could have been as efficient, if not more. We have modified the sentence to delete aspects related to energy losses.

SUPPORTING INFORMATION

Page S10 – "2.1.1. (Spectro)electrochemical Characteristization"
Figure S12 caption – "Small differnces"
Page S18 – "In addition to the comparsion"
Page S31 – "while in acetone, butyronitrile, and butyronitrile the values were measured"

Response: All of these typographical errors were now corrected and both the manuscript and SI were further proofread.

Additional Questions:
Quality of experimental data, technical rigor: Top 1%
Significance to chemistry researchers in this and related fields: Top 1%
Broad interest to other researchers: Top 1%
Novelty: Top 1%
Is this research study suitable for media coverage or a First Reactions (a News & Views piece in the journal)?: Yes

Reviewer: 3

Recommendation: Publish in ACS Central Science without change.

Comments:
This manuscript describes the solvent dependence of the excited state dynamics of a long lived Fe(III)–anthracene dyad. The work is expertly done and the manuscript is well written. I suspect that this paper will be of great interest to the photochemical community.

I recommend acceptance of the manuscript in its present form. However, the authors might decide to include a brief paragraph (or a few sentences) that relates their discussion of the work term and the driving force for the formation of the CSS to the classical reorganization energy in Marcus theory. It is hard to imagine that the reorganization energy is the same in both solvents. I think such a description would improve the manuscript.

Response: We appreciate this comment and have estimated the reorganization energy using the dielectric continuum model. Using this model, we estimated reorganization energies that were between 1.01 eV for acetonitrile, 0.95 for acetone, 0;91 for butyronitrile, 0.74 for DCM and 0.72 for THF. Thes values are in line with typical reorganization energy for electron transfer but point towards clear differences between the solvents, as suggested by the reviewer. Taking these values into consideration, and using classical Marcus theory with a preexponential factor of 10^{13} s^{-1}, we estimated electron transfer rate constant that were two orders of magnitude larger for DCM and THF (4-8 10^{11} s^{-1}) than in the other solvents (10^{9} to 10^{10} s^{-1}), in line with our experimental results. The preexponential factor could be different within the dyad, thereby proportionally influencing the respective electron transfer rate constants, but the orders of magnitude difference between dichloromethane, THF and the other solvents would still be present. Given the uncertainties in the pre-exponential factors, we have only qualitatively described the trend in the manuscript.

Additional Questions:
Quality of experimental data, technical rigor: Top 1%
Significance to chemistry researchers in this and related fields: Top 10%
Broad interest to other researchers: Top 10%
Novelty: Top 10%
Is this research study suitable for media coverage or a First Reactions (a News & Views piece in the journal)?: No Reviewer: 4

Recommendation: Publish in ACS Central Science after minor revisions noted.

Comments:
This paper follows a recent publication in JACS on the same compound, where the focus was on the population of an anthracene-localized triplet excited state after excitation of the iron(III) complex into its lowest 2LMCT state (reference 14). From what I gather, the previous JACS article concentrated on the decay behavior, specifically the long lifetime, of this dark triplet state on anthracene. In contrast, the current manuscript now shifts focus to the formation of this triplet state.

The experimental evidence supporting the mechanistic proposal presented in Figure 5b is, in my view, convincing. The relevant spectral signatures are directly observable, and the kinetics clearly reflect state-to-state transitions: the disappearance of one species coincides with the appearance of the expected follow-up product.

To my knowledge, the mechanism proposed in Figure 5b has not been directly observed before. While the case of low-spin d6 systems shown in Figure 5a is well established and conceptually analogous in some respects, there is a notable conceptual difference. Specifically, the mechanism in Figure 5b provides a direct pathway from a doublet excited state to a triplet excited state without the involvement of an intersystem crossing (ISC) event. This was new to me, and I find it particularly intriguing.

In my opinion, the paper would benefit from a broader discussion of the relevance of this mechanism. Why has this type of transition not been observed previously, despite the existence of comparable systems in the literature, such as iron(III) photosensitizers combined with polyaromatic hydrocarbons, whether in bimolecular assemblies or covalently linked unimolecular systems? Furthermore, extrapolating from this somewhat greater context going beyond the author's own work, how might this mechanism impact the future of photochemistry? What is its particular significance for the photochemistry of first-row transition metals in other oxidation states or valence electron configurations?

Including this broader discussion would, in my view, help the editor better assess the importance and potential impact of the work presented here, especially in relation to the recently published JACS paper on the same compound.

Response: We would like to thank the reviewer for their appreciation of our work. We have added a short paragraph (while keeping within the character limits of ACS Central Science) in the conclusion. The fact that it was not observed previously probably stems from the energy location of the charge separated state compared to the energy of the excited state. The excited iron photosensitizer is a potent photo-oxidant that favors excited-state electron transfer and the formation of the CSS. This in unlike other photosensitizers based on earth abundant elements. In the bimolecular case, we noted in the manuscript that we observed a small portion of the CSS, but mostly direct energy transfer. It therefore appears that the relative energy level of the different states is not the sole contributor to this mechanism, but probably the distance and orientation/geometry of the final energy acceptor compared to the iron center are also important. These are aspects that we seek to investigate in the near future and hopefully provide further useful insight.

Additional Questions:
Quality of experimental data, technical rigor: Top 10%
Significance to chemistry researchers in this and related fields: Moderate
Broad interest to other researchers: Moderate

Novelty: Top 10%
Is this research study suitable for media coverage or a First Reactions (a News & Views piece in the journal)?: No
